# Bony labyrinth morphology reveals deep population history and isolation among *Homo sapiens* in prehistoric northeastern Africa (44–3 ka)

**DOI:** 10.1038/s41467-026-76423-5

**Published:** 2026-08-12

**Authors:** Nicolas Martin, Petr Velemínský, Alessandro Urciuoli, Lenka Varadzinová, Matthieu Honegger, Stanley H. Ambrose, Daniel Antoine, Steven A. Brandt, Jaroslav Brůžek, Jessie Cauliez, Henri Duday, Frederick Grine, Petra Brukner Havelková, Joel D. Irish, Friederike Jesse, Laura Maréchal, Bruno Maureille, Isabelle Ribot, Rebeka Rmoutilova, Hélène Rougier, Frédéric Santos, Adrien Thibeault, Donatella Usai, Nicolas Vanderesse, Ladislav Varadzin, Rebecca J. Whiting, Isabelle Crevecoeur

**Affiliations:** 1 PACEA, UMR 5199, University of Bordeaux, CNRS, Ministère de la Culture, Pessac, France; 2 Department of Anthropology, Natural History Museum, National Museum, Prague, Czech Republic; 3 Department of Paleontology, University of Zurich, Zürich, Switzerland; 4 Institut Català de Paleontologia Miquel Crusafont (ICP-CERCA), Universitat Autònoma de Barcelona, Barcelona, Spain; 5 Departamento de Ciencias de la Vida, Universidad de Alcalá, Cátedra de Otoacústica Evolutiva y Paleoantropología (HM Hospitales-UAH), Madrid, Spain; 6 Division of Palaeoanthropology, Senckenberg Research Institute and Natural History Museum Frankfurt, Frankfurt am Main, Germany; 7 Czech Institute of Egyptology, Faculty of Arts, Charles University, Prague, Czech Republic; 8 Institute of Archaeology, University of Neuchâtel, Hauterive, Switzerland; 9 Department of Anthropology, University of Illinois, Urbana, IL USA; 10 Department of Egypt and Sudan, The British Museum, London, UK; 11 Anthropology Department, University of Florida, Gainesville, FL USA; 12 Department of Anthropology and Human Genetics, Faculty of Science, Charles University, Prague, Czech Republic; 13 CNRS, TRACES, UMR 5608, University Toulouse Jean Jaurès, Toulouse, France; 14 Department of Anthropology, Stony Brook University, Stony Brook, NY USA; 15 Department of Anatomical Sciences, Renaissance School of Medicine, Stony Brook University, Stony Brook, NY USA; 16 Research Centre in Evolutionary Anthropology and Paleoecology, School of Biological and Environmental Sciences, Liverpool John Moores University, Liverpool, UK; 17 University Archives, University of Wuppertal, Wuppertal, Germany; 18 Laboratory of Archaeology of Africa & Anthropology (ARCAN), Department of Anthropology, University of Geneva, Geneva, Switzerland; 19 Department of Anthropology, Laboratoire de Bioarchéologie Humaine, University of Montréal, Montréal, QC Canada; 20 Department of Anthropology, California State University Northridge, Northridge, CA USA; 21 Centro Studi Sudanesi e Sub-Sahariani ETS, Treviso, Italy; 22 Institute of Archaeology, Czech Academy of Sciences, Prague, Czech Republic

**Keywords:** Archaeology, Biological anthropology, Palaeontology

## Abstract

Understanding the population history of *Homo sapiens* in northeastern Africa has been hindered by the scarcity of human remains and ancient DNA data. Here, we analyse bony labyrinth morphology, a reliable proxy for population affinities, in 148 individuals dating from the Late Pleistocene to the Late Holocene from the Nile Valley region, the Horn of Africa, Central Africa, comparative Pleistocene fossils from Africa and Eurasia, and recent South Africans. Shape analyses reveal morphological continuity among Middle Nile Valley foragers and close affinities with earlier African *H. sapiens* fossils, suggesting deep regional ancestry. In contrast, local Neolithic food producers exhibit distinct morphologies, supporting a major demographic shift beginning 8000 years ago. A subgroup of Early Holocene Nile Valley foragers displays unusual bony labyrinth morphology and congenital inner ear-specific pathological changes consistent with possible syndromic conditions. This may result from several demographic scenarios, including prolonged endogamy and geographic isolation under environmental constraints. Comparative data from the Horn of Africa and Central Africa reveal high morphological diversity, suggesting strong population substructure and dynamic demographic trajectories during the Holocene. These findings provide rare insights into prehistoric population structure in the region, and demonstrate the usefulness of bony labyrinth morphology where ancient DNA is unavailable.

## Abstract (web summary)

Little is known of the *Homo sapiens* population history in late prehistoric northeastern Africa. Using inner ear morphology from 148 individuals, this study documents 40.000 years of human evolution, interactions and isolation in these groups.

## Introduction

As a geographic crossroads between sub-Saharan Africa, the Levant, and the Arabian Peninsula, northeastern Africa likely played a key role in population dispersals within and out of Africa during the Late Pleistocene and Holocene^1,2^. Crucially, the region's environments were profoundly influenced by climatic fluctuations during these periods. Alternating humid and arid phases transformed vast portions of the Sahara into habitable landscapes during so-called “green Sahara” periods, facilitating population movements, interregional contacts, and demographic expansions^2–4^. Conversely, arid intervals imposed constraints on mobility and settlement, possibly leading to population fragmentation into climatic refugia^5–7^.

Despite abundant archaeological and palaeoclimate evidence, the scarcity of ancient DNA (aDNA) data—and more generally of Late Pleistocene to Mid-Holocene human skeletal assemblages^8^—in northeastern Africa remains a significant obstacle to reconstructing its population history. Nearly all attempts to retrieve endogenous molecular information have been unsuccessful^9^. To date, the limited available aDNA data for the region derive from only a few individuals, all belonging to Mid-Holocene or later assemblages^10–13^.

The Middle Nile Valley (i.e., the area extending from the First to the Sixth Cataract of the Nile in southern Egypt and Sudan) is one of the few regions in northeastern Africa with large skeletal assemblages. Although aDNA is unavailable for the relevant time periods, extensive analyses of phylogenetically informative dental traits^14–19^ have revealed a complex demographic history, suggesting marked population discontinuity associated with the Neolithic transition (starting around 8 ka) in most parts of the region. Proposed exceptions of continuity—of forager-related groups in peripheral desert areas or isolated Nile contexts^14,16^—highlight the mosaic nature of population dynamics in the region and underscore the value of large morphological datasets for reconstructing population history where biomolecular evidence is limited.

Beyond the Nile Valley, broader northeastern Africa (including the Horn of Africa) and adjacent regions (e.g., Central Africa) present an equally intricate archaeological and environmental sequence for the first half of the Holocene, including major environmental fluctuations^20–26^, as well as complex and dynamic interaction networks^27–34^. However, from a biological perspective, individuals who yielded endogenous aDNA remain limited^10,12^, making morphological approaches a key alternative for investigating the population history of the region. While dental studies have provided valuable regional insights, comparative tooth samples for broader macro-regional analysis remain scarce in northeastern Africa. To further document the region's population history, this study examines another skeletal structure: the bony labyrinth. It comprises a system of cavities embedded in the petrosal part of the temporal bone that encloses the vestibular and auditory organs. Importantly, its morphology is recognised as a phylogenetically informative structure^35–40^.

Previous investigations have analysed bony labyrinth morphology in some of the earliest available Late Pleistocene African human fossils, including Nazlet Khater 2, Hofmeyr, and Ishango 37^8,40–42^. These specimens (among the few dated to Marine Isotope Stage (MIS) 3-2 in Africa^8^) exhibit a mix of features, which is not found in more recent African populations, and they align more closely with European Upper Palaeolithic (EUP) specimens than with recent African populations. Additional data, albeit limited and so far only used as comparative data for Late Pleistocene material, are also available for Early to Mid-Holocene individuals from the Horn of Africa and Central Africa^8,41^.

**Fig. 1:**
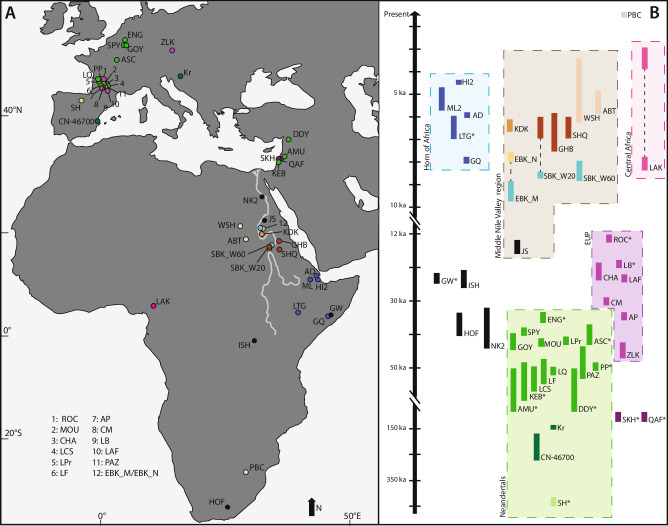
Location (A) and dating (B) of the samples included in this study. Coloured bars indicate site date ranges (Supplementary Data 1); dashed lines link multiple occupations at the same archaeological site. Abbreviations are detailed in Table 1. *Assemblages included in the 2D measurement analysis only.

Because detailed dental data are currently only available for the Middle Nile Valley, this region offers a unique opportunity to test whether the bony labyrinth reflects the same micro-evolutionary patterns previously observed in dental morphology. We therefore use the Middle Nile Valley region (with its well-documented population complexity^14–16^ and large sample of human remains available) as a core comparative framework for investigating the population dynamics across northeastern African and adjacent regions through bony labyrinth morphology. Our sample (Fig. 1, Table 1 and Supplementary Data 1) includes a total of 148 individuals (*n* = 194 bony labyrinths in total) spanning the Late Pleistocene to the Late Holocene from the Middle Nile Valley, the Horn of Africa, and Central Africa, supplemented by a recent southern African sample and Late Pleistocene comparative specimens from Africa, Europe, and the Levant. Crucially, these individuals were selected from key skeletal assemblages to maximise the resolution of regional population histories, with the European and Levantine *Homo sapiens* and Neandertal specimens included specifically to provide broader comparative context and to serve as outgroups where appropriate.

**Table 1:** Summary table of the samples included in this study

Sample	Dating	Assemblages included and abbreviations used	N	References
Late Palaeolithic Middle Nile Valley	17–13.3 ka	Jebel Sahaba (JS)	7	^117–119^
Mesolithic Middle Nile Valley	9.8–7.9 ka	El–Barga (EBK_M) Sphinx (SBK_W60) Fox Hill (SBK_W20)	20	^60–63,84,85,120,121^
Early Neolithic Middle Nile Valley (Nubia)	8–7.5 ka	El–Barga (EBK_N)	11	^60,84,85^
Early Neolithic Middle Nile Valley (Central Sudan)	7.6–6 ka	Fox Hill (SBK_W20) Ghaba (GHB)	9	^62,63,119,122–124^
Middle Neolithic Middle Nile Valley (Nubia)	6.5–6 ka	Kadruka 1 (KDK)	11	^125–127^
Middle Holocene Sudanese desert regions	7–3.3 ka	Shaqadud (SHQ) Abu Tabari (ABT) Wadi Shaw (WSH)	10	^50,128–131^
Central Africa	8.2–2.9 ka	Shum Laka (LAK)	7	^10,53^
Horn of Africa	8–4 ka	Gogoshiis Qabe (GQ) Mille–Logya 2 (ML2) Ali Daba (AD) Hara Idé 2 (HI2) Lothagam (LTG)*	10 (1)	^8,31,32,34,132–135^
**Comparative samples**				
Recent *Homo sapiens*	Present day	Pretoria Bone Collection (PBC)	30	^136^
Late Pleistocene African fossils	44–20 ka	Nazlet Khater 2 (NK2) Ishango 37 (ISH) Hofmeyr (HOF) Guli Waabayo (GW)	4	^1,8,40,133,137–140^
Qafzeh/Skhul	120–90 ka	Qafzeh (QAF)* Skhul (SKH)*	(11)	^141,142^
European Upper Palaeolithic (*Homo sapiens)*	45–13 ka	Cro–Magnon (CM) Pataud (AP) Chancelade (CHA) Lafaye (LAF) Zlatý kůň (ZLK) Rochereil (ROC)* Laugerie–Basse (LB)*	8 (2)	^54,143–147^
Pre–Neandertals	~430 ka	Sima de los Huesos (SH)	(11)	^148,149^
Marine Isotope Stages 6–5 Neandertals	273–120 ka	Krapina (Kr) Cova Negra (CN)	10	^57,150–152^
Late Neandertals	70–34 ka	Spy (SPY) Le Moustier (LM) Les Pradelles (LPr) Goyet (GOY) Pech de l’Azé (PAZ) La Quina (LQ)* La Ferrassie (LF)* La Chapelle–aux-Saints (LCS) Engis (ENG)* Arcy-sur-Cure (ASC)* Petit-Puymoyen (PP)* Dederiyeh (DDY)* Kebara (KEB)* Amud (AMU)*	11 (9)	^153–169^
		Total	148 (34)	

N: number of individuals included in the 3D analyses (number of additional individuals included in the 2D measurement analysis only); * assemblages with at least one individual included in the 2D analysis only (see Supplementary Data 1).

Given the interconnected nature of population history, subsistence and cultural changes, and regional climate dynamics in northeastern Africa, we adopt an integrated framework combining morphological, archaeological, and palaeoenvironmental evidence. Within this framework, we first test whether the biological discontinuity previously suggested for the Middle Nile Valley based on dental studies is also detectable when studying the bony labyrinth (i.e., whether significant morphological changes occurred during the Neolithic transition). Second, we examine affinities between Middle Nile Valley groups and populations from neighbouring regions, such as the Horn of Africa and Central Africa, using morphological variability in the former to interpret patterns of biological affinities in these regions. Finally, we discuss the observed morphological patterns in light of established palaeoclimatic trends, which alternately facilitated regional connectivity or promoted population differentiation. Rather than formally assigning samples to specific climatic phases, we assess whether periods characterised by increased aridity or climatic amelioration at the regional scale provide a plausible framework for understanding patterns of continuity, sub-structuring, or interregional affinity, where ‘similarity’ denotes morphological resemblance, ‘affinity’ potential biological relatedness, and ‘continuity’ persistence through time.

**Fig. 2:**
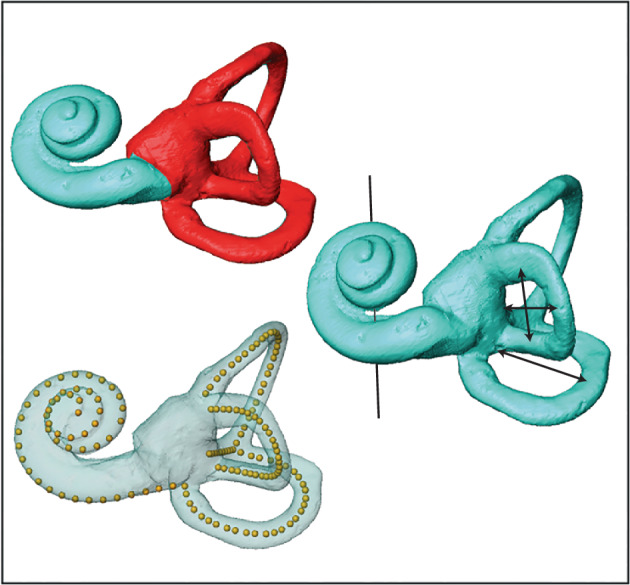
Representation of the shape comparison methods employed in this study. From top to bottom: part of the bony labyrinth (red) considered in the diffeomorphic surface matching analysis; selection of measurements and orientation included in the 2D analyses; and template used in the 3D landmark analyses.

Using three distinct bony labyrinth shape analyses (diffeomorphic surface matching (DSM), 2D analysis, and landmark-based analysis; Fig. 2) and by integrating these interconnected lines of evidence, our study provides a holistic and biologically grounded interpretation of human population history in northeastern African regions during the Late Pleistocene and Holocene.

## Results

**Fig. 3:**
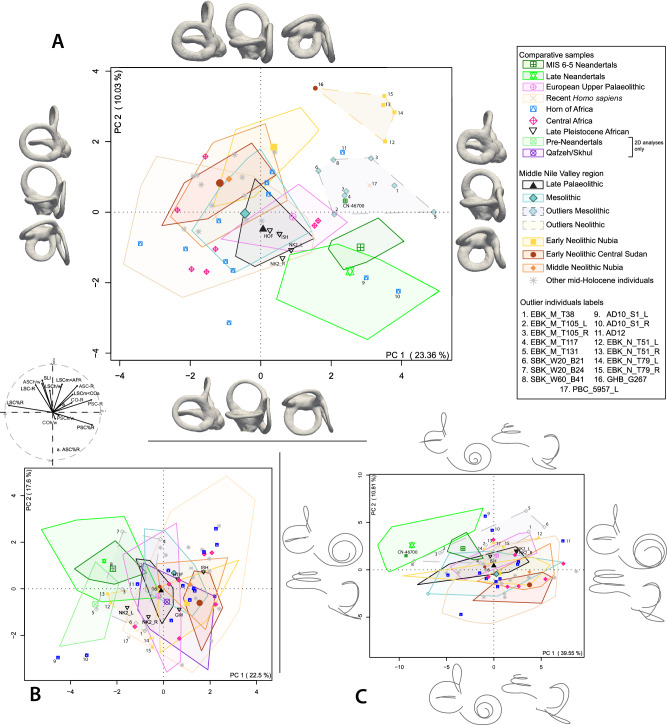
Bony labyrinth morphological comparisons. Principal component analyses computed on the deformation fields of the vestibule and semi-circular canals (SCCs) (**A**), on the whole bony labyrinth and based on 2D measurements (**B**), and on landmark Procrustes coordinates (**C**). Source data are provided as a Source Data file. In all panels, groups are represented by their convex hulls and centroids, with only relevant individual specimens shown separately. Figures displaying all individual data points are provided in the Supplementary Figs. 3,5,6 and Supplementary Data 3. Extreme morphologies (**A–C**) correspond to maximum and minimum scores along the principal component axes and are illustrated as simulated vestibule and SCC surfaces in posterior, lateral, and supero-posterior views (**A**), or as wireframes of semi-landmarks in posterior and infero-medial views (**C**). The biplot in (**B**) shows the principal component loadings. MIS = Marine Isotope Stage.

Across all three analytical approaches, we recorded similar general patterns of morphological variation in the bony labyrinth (Fig. 3; Supplementary Note S1 and Fig. S1, 2). All methods show: (1) marked Neandertal-*Homo sapiens* morphological differences; (2) broad similarities among Late Pleistocene African specimens and Early Holocene Middle Nile Valley groups; (3) discontinuity at the Neolithic transition in the Middle Nile Valley; (4) high diversity in the Mid- to Late Holocene Horn of Africa and Central Africa; and (5) a consistent subgroup of outliers from Holocene contexts in the Middle Nile Valley and Horn of Africa. Because all approaches converge on the same patterns and outlier individuals, the detailed results below focus on describing the results of the DSM analyses, with more detailed information on the 2D and landmark-based analyses in Supplementary Note S1.

The PCA performed on the deformation fields of the semi-circular canals (SCCs) and vestibule is presented in Fig. 3A. The first principal component (PC1; 23.36% of total variance) is primarily driven by morphological variation between the Neandertal samples (plotting towards the positive end of PC1) and both the African individuals and EUP *Homo sapiens* (rather found towards null and negative values on PC1). This mainly relates to a combination of characteristic morphological traits observed in Neandertals: a relatively smaller and more inferiorly positioned posterior SCC, a smaller and narrower anterior SCC, and a larger, wider lateral SCC (Fig. 3 and Supplementary Fig. S1, 2), consistent with previous findings^36,43–47^.

Within *Homo sapiens*, PC1 also captures variation between both African and European Late Pleistocene individuals, and most northeastern African Middle Holocene samples and recent South Africans. The latter groups are characterised by generally thinner SCCs, larger anterior and posterior SCCs, and a comparatively narrower lateral SCC (i.e., low and negative values along PC1; Supplementary Fig. S1). In the same way, the Middle Nile Valley Middle Holocene individuals plot at the positive end of PC2 (10.03% of variance) while those from the Late Pleistocene and Early Holocene tend to cluster closer to the null values of this component. Morphologically, this relates to the Mid-Holocene individuals having smaller and thicker anterior and lateral SCCs, as well as a larger, rounder, and thicker posterior SCC (higher positive PC2 scores; Supplementary Fig. S1).

Importantly, interpretation of this secondary axis does not rely on the DSM analysis alone. Although PC2 in the DSM framework does not reach statistical significance (see Methods section), the corresponding axes in the landmark-based and 2D analyses are statistically supported, and all three approaches display congruent patterns of differentiation among the groups under study (Supplementary Note S1﻿). PC1, therefore, captures the primary axis of variation across methods, whereas PC2 provides a secondary exploratory axis of variation within *Homo sapiens*, interpreted in light of this cross-method convergence rather than as an isolated exploratory dimension.

The Late Pleistocene Nazlet Khater 2, Ishango 37, Guli Waabayo, and Hofmeyr exhibit strong similarities in the morphology of their vestibule and SCCs (Fig. 3A). These consist of a relatively wider but shorter anterior SCC; medium to short common crus, resulting in an intermediate position of the posterior SCC relative to the lateral SCC (though still higher than in Neandertal individuals); and a relatively small width of the posterior SCC. These characteristics place them closer to the EUP *Homo sapiens* fossils than to the majority of the individuals in the recent South African comparative sample (Fig. 3A). Although these individuals exhibit overall morphological resemblance, notable individual differences are observed: Ishango 37 possesses a medio-laterally elongated posterior SCC relatively to the other canals, while Hofmeyr shows a narrower lateral SCC compared to the other canals (Supplementary Fig. S1, 2). Similar patterns emerge when cochlear parameters are considered (Fig. 3B, C, Supplementary Note S1, Fig. S2), with both strong similarities and marked differences among the four fossils, as highlighted in previous studies^8,40–42^.

**Fig. 4:**
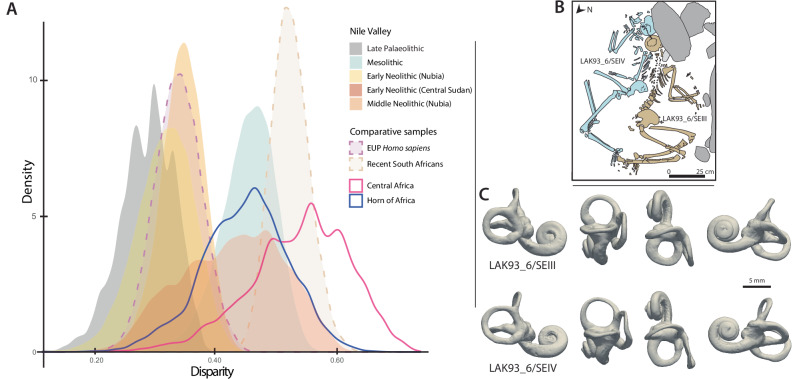
Intra-group morphological variation. **A** Density plot of morphological disparity for the *Homo sapiens* bootstrapped (20,000 replicates) samples included in the study. Illustration of the major biological variability in Central Africa with drawing of a double burial from Shum Laka (Cameroon, LAK93_6/SEIII and LAK93_6/SEIV; (**B**), and views of the segmented bony labyrinths of these individuals (**C**). **B** is derived from ref. ^53^ (original drawing by I. Ribot). Source data for (**A**) are provided as a Source Data file.

The Mid-to-Late Holocene individuals from the Horn of Africa and Central Africa exhibit major morphological diversity (Fig. 3 and Supplementary Fig. S2, 5, 6). This includes variation in the size, position and orientation of the SCCs, as well as in the dimensions and coiling of the cochlea. In both regions, this overall heterogeneity and high degree of variation are evident when considering intra-group morphological disparity (Fig. 4A). These results show high disparity values for both regions, findings which are higher than in the EUP *Homo sapiens*, and similarly high to that seen in the recent South African sample.

**Fig. 5:**
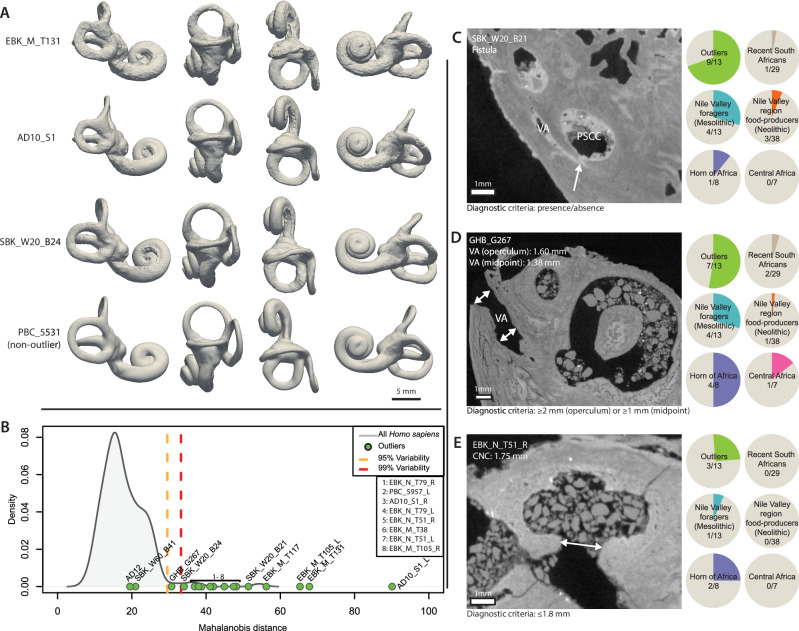
Outlier individuals. **A** Views of the segmented bony labyrinths with unusual morphologies, compared to a non-outlier recent individual. **B** Mahalanobis distances between all *Homo sapiens* individuals (*n* = 114) and the centroid, compared to the individuals with unusual morphologies. **C–E** Associated inner ear-specific pathological changes and respective frequencies within the samples studied (**C** Fistula between the vestibular aqueduct (VA) and posterior semi-circular canal (PSCC); **D** Enlarged VA; **E** Cochlear nerve canal (CNC) stenosis). Source data for (**B**) are provided as a Source Data file.

Finally, in the diffeomorphic surface matching (DSM) analysis, a subgroup of 13 Holocene individuals (represented by 17 bony labyrinths in total) appears as outliers—plotting towards high positive values along PC1 (Fig. 3A)—with a majority of them also consistently appearing as outliers in the other shape analyses (Fig. 3B, C and Supplementary Note S1), and also when focusing on the *Homo sapiens* (Supplementary Fig. S7) or archaeological samples (Supplementary Fig. S8) solely. These primarily originate from Mesolithic/Early Holocene assemblages in the Middle Nile Valley (*n* = 7), with some from Mid-Holocene sites in the Middle Nile Valley (*n* = 3) or the Horn of Africa (*n* = 2), while one is part of the recent South African sample (Table 2). They are characterised by 1) a markedly shorter posterior SCC, 2) a shorter and thicker common crus, 3) a slightly reduced anterior SCC, 4) a slightly more tightly coiled and posteriorly oriented cochlea, and 5) little to no variation on the lateral SCC compared to the rest of the *Homo sapiens* sample (Figs. 3 and 5A, Table 2, Supplementary Note S1 and Fig. S9–12).

**Table 2:** Bony labyrinth measurements of the outlier individuals and associated pathological changes

Individual	Group	Age	Sex	Bilateral	Pathological changes	SLI	ASCh	ASCw	PSCh	PSCw	LSCh	LSCw	COh	COw	V length	V width
EBK_M_38	EHNV	Ad	M	/	Fis.	55.81	4.94*	5.99*	5.53*	4.72*	3.80*	4.37*	5.21	4.39	6.15	2.32*
EBK_M_105_L	EHNV	Ad	M	yes	EVA, CNC	60.75^ǂ^	5.52	6.13*	5.84	4.84*	4.56	4.94	5.76^ǂ^	4.77^ǂ^	5.8	3.41^ǂ^
EBK_M_105_R					Fis., CNC	56.06	5.24*	6.24*	5.73*	5.40*	4.74	5.30	5.23	4.61^ǂ^	6.36	3.45^ǂ^
EBK_M_117	EHNV	Ad	F	/	Fis., EVA	64.85^ǂ^	6.27	6.93	5.58*	5.65	4.45	5.00	5.34	4.85^ǂ^	6.65^ǂ^	2.62
EBK_M_131	EHNV	Ad	F	/	Fis.	53.11	4.50*	6.06*	4.73*	4.45*	4.40	4.67	5.15	4.34	6.26	2.59
EBK_N_51_L	MHNV	Juv	?	yes	Fis., EVA, CNC	57.14	5.32	6.06*	5.21*	5.16*	4.37	5.01	4.89*	3.89*	6.03	2.85
EBK_N_51_R					Fis., EVA, CNC	57.19	5.43	6.09*	4.92*	5.00*	4.29	4.90	4.86*	3.90*	6.32	3.12^ǂ^
EBK_N_79_L	MHNV	Ad	F	yes	/	52.8	4.50*	6.82	4.89*	5.58	3.69*	5.00	5.13	4.00	7.21^ǂ^	3.11^ǂ^
EBK_N_79_R					Fis., EVA	54.55	4.47*	6.59	5.01*	5.55*	3.63*	4.78	4.98	4.22	6.68^ǂ^	3.33^ǂ^
SBK_W60_B41	EHNV	Ad	M	/	Fis., EVA	59.44	5.07*	6.43	5.08*	5.60	5.28	4.66	5.67^ǂ^	4.41	6.43	2.84
SBK_W20_B21	EHNV	Ad	M	/	Fis.	51.25	4.90*	6.39	5.96	5.34*	4.54	5.17	5.61^ǂ^	4.25	6.12	3.26^ǂ^
SBK_W20_B24	EHNV	Ad	M	/	-	65.31^ǂ^	6.22	6.86	4.94*	5.31*	4.65	5.28	5.37	4.37	6.3	3.08^ǂ^
GHB_G267	MHNV	Ad	M	/	EVA	63.28^ǂ^	5.51	6.54	4.97*	5.55*	3.66*	4.43*	**4.60**	4.16	6.14	2.38*
AD12	MHHA	?	?	/	CNC	59.96^ǂ^	5.80	6.23*	5.10*	5.68	4.10	4.70	5.01	3.68*	6.7^ǂ^	3.27^ǂ^
AD10_S1_L	MHHA	Juv	?	yes	EVA	39.26*	5.04*	6.28*	4.54*	3.93*	4.10	5.71^ǂ^	5.02	3.73*	6.38	3.04
AD10_S1_R					Fis., EVA	43.09*	5.01*	6.60	4.73*	4.40*	3.99	5.44^ǂ^	5.02	3.77*	6.31	3.13^ǂ^
PBC_5957_L	ModSA	Ad	F	no	Fis.	48.96	5.11*	6.46	5.25*	4.89*	3.92	5.15	5.26	4.09	6.56^ǂ^	3.49^ǂ^
Rest of the *H. sapiens* sample (n = 114)					Mean	53.01	5.93	6.86	6.39	6.12	4.38	4.96	5.27	4.23	6.08	2.79
					SD	6.63	0.67	0.49	0.63	0.55	0.48	0.44	0.31	0.31	0.43	0.29
					Min	36.28	4.50	5.26	4.93	4.78	3.32	4.00	4.33	3.54	4.94	1.88
					Max	69.44	8.18	8.28	8.88	7.54	6.07	6.03	6.15	6.14	7.28	3.74

* Values lower than the rest of the *Homo sapiens* sample mean − 1 SD; ^ǂ^ values higher than the rest of the *Homo sapiens* sample mean + 1 SD; bold values represent lower/higher measurements than any other individual from the rest of the *Homo sapiens* sample

*EHNV* Early Holocene Middle Nile Valley, *MHNV* Middle Holocene Middle Nile Valley, *MHHA* Middle Holocene Horn of Africa, *ModSA* recent South Africa, *Ad* Adult, *Juv* Juvenile, *Fis.* Fistula between the vestibular aqueduct and posterior SCC, *EVA* enlarged vestibular aqueduct, *CNC* cochlear nerve canal stenosis, measurements abbreviations were taken from ref. ^38^, *V* *length/width*: dimensions of the vestibule.

## Discussion

### The Middle Nile Valley and adjacent desert regions

Notable differences were identified across the different time periods and sites represented (Fig. 3, Supplementary Note S1 and Fig. S2–4). This is particularly relevant when comparing the Middle Nile Valley foragers (Late Pleistocene-Early Holocene) and food producers. These differences are also supported by the PERMANOVA results, mostly displaying significant values between forager and food producer groups (Supplementary Table S2). Considering the high phylogenetic value of the bony labyrinth morphology^35–40^, we suggest that the clear patterns highlighted by the PCAs (Fig. 3) and PERMANOVA results (Supplementary Table S2) highlight major biological discontinuity at the Neolithic transition in the region and, in other words, the emergence of food-producing practices in the Middle Nile Valley, contemporaneous to migrations starting ca. 8000 years ago^48,49^. This further confirms previous research on dental morphology^14–16^, and also recent aDNA data from Egypt suggesting some gene flow towards the region sometime before the Late Holocene^13^.

Neolithic individuals (*n* = 8) dating to 6–4 ka in the Sudanese Eastern Sahara margins (at the Abu Tabari and Wadi Shaw sites, respectively ca. 300 and 360 km west of the Nile) display considerable morphological variation. Some of them clearly align with the Middle Nile Valley Mesolithic/Late Palaeolithic variation, while others align more with the morphological variation of the Neolithic food producers (see grey asterisks in Fig. 3 and Supplementary Fig. S3, 4). This is further confirmed by typicality probabilities (Supplementary Table S3), highlighting individuals with strong affinities with either the Mesolithic population or the food producers from the Sudanese assemblages included. This suggests the persistence in the region of a forager-related population several millennia after the Neolithic transition, but also interaction between forager-related and food producer-related populations there. In this regard, these findings parallel previous dental studies that identified two biologically distinct populations cohabitating in this region and at those sites^16^. Of particular interest to the latter is individual 83.110–18.1 from Wadi Shaw, radiocarbon-dated to ca. 3.5 ka (3200 ± 120 BP^50^), showing an “in-between” morphology of the bony labyrinth, and affinities with both foragers and food producers from the Middle Nile Valley (Supplementary Fig. S3, 4 and Table S3). As the most recent individual from the region in our sample, this case may represent a late persistence of forager-related morphological affinities in this part of the Sudanese Western Desert, extending the presence of such groups in the region by roughly a millennium beyond what has been documented previously.

Similar results appear for the two 7–6 ka Neolithic individuals from Shaqadud, about 45 km southeast of the Nile and 115 km northeast of Khartoum in Central Sudan (Fig. 1). Although these individuals were buried close to each other in the same burial site and have similar dating, one of them shows strong affinities with the Middle Nile Valley Neolithic individuals (SHQ_Burial 2), while the other also shows similarities with the Mesolithic foragers from the region (SHQ_Burial 3). This heterogeneity may represent settlement processes east of the Nile that were to some extent similar to what was highlighted for the desert margins west of the Nile, and cohabitation of individuals with distinct biological affinities—one related to the Middle Nile Valley foragers, the other to the new food-producing groups—at the same time in this region. This would therefore imply a persistence of forager-related group(s) in the savannahs east of the Nile in Central Sudan, or at least the persistence of morphological traits associated with forager populations within later food-producing populations in the area. Additional investigation on larger samples from these eastern regions will, however, be needed to discuss this further.

A key aim of this study was to assess whether bony labyrinth morphology reflects the same micro-evolutionary population patterns previously observed in enamel-dentine junction (EDJ) and other dental characteristics for the Middle Nile Valley^14–16^. Our results show a close correspondence between the two approaches (based on the same populations and, in some cases, the same individuals; Supplementary Fig. S13), indicating that the bony labyrinth captures similar population signals despite its overall evolutionary stability. However, it is noteworthy that the morphological overlap among Middle Nile Valley groups appears more pronounced when assessed through bony labyrinth morphology than EDJ shape (Supplementary Note S3 and Fig. S13). This may be attributable to varying degrees of biological and morphological diversity captured by each structure, indicating that the bony labyrinth could be a more evolutionarily conserved structure and exhibit reduced inter-population variation in comparison to the EDJ.

The implications of these slight differences are significant: while both structures are valuable for reconstructing population history, their apparent differing sensitivity to population-level variation means that they may capture distinct evolutionary signals. The EDJ morphology, being more variable, might better reflect recent or finer-scale evolutionary processes, whereas the more stable bony labyrinth could preserve deeper or broader patterns of lineage continuity. This highlights the importance of combining multiple morphological systems to achieve a more nuanced understanding of population history and evolutionary dynamics. Importantly, it also validates the use of bony labyrinth morphology in regions or periods where dental data are limited or unavailable (e.g., Horn of Africa, Central Africa, or Late Pleistocene contexts), providing additional lines of evidence in situations with limited alternative data.

### Late Pleistocene African individuals

The set of unique characteristics found in the bony labyrinths of Nazlet Khater 2, Ishango 37, Guli Waabayo and Hofmeyr, highlighting both major similarities and marked differences, further supports the results from previous research suggesting these individuals may reflect deep population substructures among Late Pleistocene groups across Africa^8,40–42^. Similarly, the major similarities between the EUP *Homo sapiens* and these contemporaneous individuals from Africa may provide insights into the possible population background of these early European lineages, based on the morphological affinities observed in the present analyses. Indeed, this may suggest that the EUP *Homo sapiens* shared common ancestry with, or descended from, African populations like those of Nazlet Khater 2 and Hofmeyr, further supporting the idea of a complex and regionally diverse source population within Africa for the early peopling of Europe^35,51,52^.

Interestingly, despite the major variation reported among all four Late Pleistocene African individuals, they consistently plot within or near the range of morphological variation of the Late Pleistocene Middle Nile Valley population (i.e., from Jebel Sahaba). This is of particular interest, as it suggests potential biological continuity or close affinities between the Jebel Sahaba population and earlier African groups. Moreover, the morphological similarities with older individuals observed in the Sudanese Late Pleistocene individuals are further supported by their resemblance to the EUP *Homo sapiens* also included in this study. Altogether, this suggests deep and ancient roots of the Jebel Sahaba individuals in the African population history.

Furthermore, when considering the marked homogeneity and apparent continuity between Late Pleistocene and Early Holocene foragers in the Middle Nile Valley, both in this study and previous research^15,16^, these findings are consistent with longstanding regional ancestry for these forager populations. In this regard, and considering their limited or even absent contribution to later populations^14–16,19^, it is plausible that the Middle Nile Valley foragers represent part of deeply divergent population lineages, potentially comparable to the hypothesised “ghost Green Saharans”, which remain poorly characterised in palaeogenetic records^10,11^. The individuals described here may thus offer rare morphological insights into an ancient northeastern African population whose genetic legacy has yet to be fully resolved.

### The Horn of Africa and Central Africa in the light of the Middle Nile Valley framework

The Central African individuals included here originate from the Shum Laka assemblage in Cameroon (*n* = 7)^53^, and showed major morphological variation. Part of the latter appears to be reflected in the chronological differences between these individuals. Specifically, LAK93_2/SEI and LAK93_2/SEII (dated to 8.2–7.6 ka^10,53^) plot closer to/within the Middle Nile Valley Mid-Holocene morphological range of variation, and relatively distant from the other and later individuals from the same site (dated to 3.9–2.9 ka^10,53^; Fig. 3, and Supplementary Fig. S5). Nevertheless, even within these later individuals, significant heterogeneity is evident. Notably, two individuals from the same double burial (LAK93_6/SEIII and LAK93_6/SEIV) show markedly distinct morphologies (Fig. 4B, C) and therefore occupy contrasting positions in PCA morphospace, plotting in different regions of the overall morphological variation (Supplementary Fig. S5).

Similar results emerge from the Horn of Africa sample, where individuals (*n* = 10) from roughly the same time period and geographic area show markedly different bony labyrinth morphologies. Even within the same assemblage (i.e., Ali Daba in Djibouti), individuals show major differences in the relative size, shape and orientation of the SCCs as well as in the dimensions and coiling of the cochlea (Fig. 3 and Supplementary Figs. S1, 2, 6).

Interestingly, their disparity metrics are also much higher than the EUP *Homo sapiens* variation, though documented to include diverging human lineages (e.g., refs. ^54–56^) and closer to the high disparity recorded within the recent South African samples, including individuals from varied origins. In this regard, and despite the inclusion of only a few individuals in our analyses to date, the high disparity values and broad dispersion of individuals in morphospace (Fig. 3 and Fig. 4A) suggest high morphological and biological diversity within these limited time periods and geographic areas. This pattern may reflect underlying population substructure or heterogeneous demographic histories within these regions.

More broadly, the high morphological disparity documented in the PCA distributions and disparity analyses (Fig. 3 and Fig. 4A) for these few individuals from both the Horn of Africa and Central Africa more closely resemble the inter-group differences observed in the Middle Nile Valley, such as those between foragers and food producers, than the expected range of variation within a single, homogeneous population. These exploratory results suggest that both regions may have been home to multiple, biologically distinct groups with complex population histories, as documented for the Middle Nile Valley. Such disparity may reflect deep population substructure, diverse regional demographic trajectories, and could also be compatible with migration events during the Holocene, which is also consistent with the limited genetic evidence available^10,12^. Additionally, the local archaeological records (e.g., refs. ^27–32^) highlight complex cultural networks and suggest major mobility of the first food-producing populations from the Horn of Africa. More importantly, these studies suggest that early food-producing practices—marked by a combination of foraging activities such as hunting, fishing, and gathering, alongside the emerging development of livestock herding^31,33,34^—most likely spread into the region from either Sudan or the Arabian Peninsula^27–32^. While these biological and archaeological patterns hint at a rich and dynamic human history in both regions, the current sample size remains limited. More data will be essential to confirm these trends, clarify the extent and nature of population diversity across northeastern Africa during the Holocene, and infer macro-regional population affinities.

### Holocene outlier individuals and pathological evidence

As a result of their unique morphological characteristics, the identified subgroup of 13 outlier individuals plots outside the range of variation observed in the rest of the *Homo sapiens* sample (*n* = 115 individuals, Figs. 3 and 5B) and appear closer to Neandertals in the morphospace, even though they are obviously not phylogenetically related to the latter. Interestingly, among the Neandertal sample, only one individual is found within the range of variation of these outliers with respect to the vestibule and SCCs morphology: CN-46700. This individual was published as presenting inner ear-specific pathological changes—i.e., SCCs dysplasia and deformation associated with a syndromic condition^57^. A comparison of the height and width of each SCC reveals that almost all these Holocene individuals possess posterior SCC dimensions smaller than any other *Homo sapiens* in the sample (Table 2), which may indicate posterior SCC hypoplasia^58,59^. Similarly, the reduced dimensions of the anterior SCC may also suggest some level of hypoplasia. In contrast, the lateral SCC appears relatively unaffected.

Regarding cochlear dimensions, some individuals show a slightly enlarged cochlea, while in others it is slightly reduced. Vestibular measurements^58^, particularly width, indicate that nearly all these individuals present a slightly larger vestibule compared to the rest of the *Homo sapiens* sample (Table 2).

Osteological assessment shows that the majority of these individuals were adults (11 out of 13)^60–65^. Among the five individuals for whom both temporal bones were available, four displayed this unusual morphology bilaterally. Despite the fragmentary nature of the skeletal remains, no other skeletal anomalies or pathological changes were observed on the cranial or infra-cranial skeleton. However, microcomputed tomography (µCT) scans of the petrous bones revealed several inner ear-specific pathological changes in nearly all these individuals (Fig. 5C–E). These consist of an enlarged vestibular aqueduct (EVA)^66^, and/or a fistula between the vestibular aqueduct and posterior SCC^67^, and/or a stenotic cochlear nerve canal (CNC)^68^. These traits were recorded following standard medical procedures and diagnostic criteria (see Methods section). While fistulas may result from middle ear infection^69,70^, EVA and CNC stenosis are congenital conditions^68,71–73^. Almost all the outliers (12 out of 13) displayed at least one of these pathological changes, often in combination and either unilaterally or bilaterally (Table 2, Supplementary Table S4). In contrast, these conditions are either absent or rare in the remainder of the *Homo sapiens* sample (14 out of 115), with the few other cases occurring almost exclusively (8 out of 14) among individuals with no sign of particular bony labyrinth deformation from Mesolithic Middle Nile Valley and Mid-Holocene Horn of Africa assemblages (Fig. 5C–E, Supplementary Fig. S14 and Table S4).

Taken together, these consistent morphological and inner ear-specific pathological changes are compatible with the presence of a developmental alteration affecting the petrous bone and inner ear. Several known syndromes feature SCC hypoplasia, and/or EVA, and/or CNC stenosis^74–77^, though the available skeletal and imaging evidence does not allow attribution to a specific syndrome, nor does it exclude alternative developmental pathways. Such syndromes result from genetic mutations, mostly inherited in an autosomal dominant pattern, and may lead to varying degrees of sensorineural hearing loss or impairment. Several of these syndromes are also documented to be highly prevalent, or even appearing de novo, in consanguineous families (e.g., refs. ^78–83^).

Interestingly, the majority of the individuals displaying unusual bony labyrinth morphology—and more generally presenting congenital pathological changes—originate from Early Holocene Middle Nile Valley assemblages (52.3% of these individuals showed at least some deformation of the bony labyrinth or one of the inner ear-specific congenital pathological changes investigated; see Supplementary Table S4 for details on the individuals concerned). These individuals are present at the three sites investigated within the region and time period, including two assemblages located only a few kilometres apart (e.g., Sphinx and Fox Hill in the western Jebel Sabaloka region) as well as one separated from the former two by over 400 km (El-Barga in Nubia; Fig. 1). The associated contexts also share a relatively homogeneous chronological range, dating between 9.8 and 7.9 ka (see Table 1). In contrast, these traits are significantly less common in the 8 to 3.3 ka Neolithic population from the region, including the desert margins (9.8%; Fig. 5C–E and Supplementary Table S4) and the modern-day South African comparative sample (10.0%).

Previous dental^15,16^, archaeological^5,84–87^, and palaeoenvironmental^2,5,20^ data suggest that the Middle Nile Valley foragers belonged to a biologically homogeneous population, interconnected at the regional level during the Early Holocene, and which may have been constrained by environmental factors for several millennia during the Late Pleistocene. In this context, the high frequency of congenital, genetically encoded anomalies (i.e., rare and highly heritable traits) observed among these Early Holocene foragers is compatible with the hypothesis of long-term intra-regional interactions between these forager groups.

However, considering the palaeoenvironmental record and documented geographic and climatic constraints over these groups at the end of the Pleistocene, the relatively high frequency of congenital anomalies and unusual bony labyrinth morphologies documented in the Early Holocene Middle Nile Valley assemblages (Fig. 3; Table 1 and Supplementary Table S4) may also be compatible with a range of demographic scenarios, including reduced population connectivity, structured interaction networks, and local demographic processes such as drift or endogamy in small or subdivided populations. Over generations, such processes may, in principle, have increased the likelihood that rare mutations would rise in frequency within the population. Such scenarios could contribute to the observed distribution of congenital pathological changes and the potential syndrome documented in these populations. The presence of similar pathological conditions in Late Pleistocene individuals from the region—one individual from Jebel Sahaba and Nazlet Khater 2 (Supplementary Table S4)—could represent early occurrences of similar congenital conditions, which may have become more frequent in later, Early Holocene populations.

With the onset of the Holocene, improving climatic conditions also likely expanded opportunities for interaction. Archaeological^84–87^ and bioarchaeological^88^ evidence points to complex social behaviours among these forager groups—including probable inter-group marital systems and extensive exchange networks—which could have promoted regional connectedness while still allowing for localised population structuring, which may have influenced the transmission of these genetically encoded traits. It is plausible that climatic constraints and later social dynamics may have influenced patterns of population connectivity in the region. If so, such demographic processes could have affected the distribution of the rare congenital traits documented during the Early Holocene. Nevertheless, the present anatomical evidence alone cannot distinguish among demographic isolation, structured interaction networks, founder effects, or other evolutionary mechanisms.

Moreover, as mentioned earlier, a few other cases of individuals displaying unusual bony labyrinth morphologies and/or congenital pathological changes were found outside the Middle Nile Valley Early Holocene population. For the Mid-Holocene/Neolithic sample from the region, three individuals exhibited an ‘outlier’ bony labyrinth morphology, and another displayed EVA (Fig. 3 and Fig. 5, Table 2, Supplementary Fig. S11 and Table S4). Three of these individuals originate from El-Barga in Nubia, a site that yielded succeeding Mesolithic and Neolithic occupations^60,84,85^. In light of the low frequency of these traits among the remainder of the Neolithic population in the Middle Nile Valley (i.e., only found in one individual from Ghaba, Central Sudan), along with the significant biological discontinuity identified between the last foragers and the first food producers in the region^14–16,19^, these few individuals might indicate some isolated, limited biological interaction or gene flow between the previous foragers and new Neolithic populations.

Given the two individuals from the Horn of Africa who also exhibit similar unusual morphology and the four additional individuals from the region also displaying congenital pathological changes (Fig. 5C–E and Supplementary Table S4), establishing parallels between the Middle Nile Valley populations and these individuals is challenging. Considering the substantial distance (i.e., both geographically and temporally) between these populations, along with the distinct biological signal of AD10_S1 (consistently plotting further from the rest of the outlier individuals), and the few individuals available so far for the region, it would be hazardous to suggest direct biological relatedness between these individuals and the Sudanese foragers included in this study. However, archaeological and palaeoenvironmental data from the Horn of Africa^20–29,31–33^ indicate a narrative similar to that of the Middle Nile Valley, characterised by harsh environmental constraints, probably leading to long-standing geographic isolation and population substructure. In this context, the presence of similar bony labyrinth morphology and associated congenital pathological changes in this region could reflect demographic processes broadly comparable to those proposed for the Middle Nile Valley, even if independent regional dynamics cannot be excluded.

Although the patterns documented here are clear and internally consistent, several limitations should be acknowledged. Inner ear anomalies can arise through different genetic or developmental pathways^58,59,66–78^, and the available skeletal evidence does not allow us to distinguish among them with complete certainty. Nevertheless, the morphological consistencies, the elevated frequency of congenital inner ear anomalies in Early Holocene Middle Nile Valley and Middle Holocene Horn of Africa assemblages, and their broad geographic distribution indicate that these traits were not isolated occurrences but reflect a wider demographic context. Multiple evolutionary scenarios remain possible (such as reduced population connectivity, small effective population size, or localised founder effects) but the anatomical evidence indicates that these populations may have experienced a combination of demographic and environmental conditions that influenced their biological variability. These findings reveal an underexplored dimension of population history in northeastern Africa and beyond, highlighting the value of inner ear morphology and pathology as informative markers of past demographic structure.

### Concluding remarks

This study underscores the valuable insights that morphological analyses can offer, particularly in regions where aDNA is unavailable. Our findings demonstrate that, even in the absence of molecular data, bony labyrinth morphology can yield remarkable information about population history and micro-evolutionary processes—ranging from micro-regional patterns to broader chronological and geographic scales. Moreover, the results highlight the close parallel and complementary nature of dental and bony labyrinth data, reinforcing the strong phylogenetic signal inherent in both structures.

These results offer insights into population structure and evolutionary processes in northeastern African regions during the Late Pleistocene and Holocene. Although the present dataset constitutes one of the largest collections of bony labyrinths from the region, certain areas (e.g., the Lower Nile Valley) remain underrepresented. Our interpretations, therefore, primarily pertain to the Middle Nile Valley and the specific regions sampled here. Nevertheless, our findings reveal significant morphological differences between Middle Nile Valley foragers and food-producing populations, pointing to major biological discontinuity at the Neolithic transition. However, despite this overall pattern of discontinuity, we highlight evidence of rare and localised instances of potential admixture or continuity between the last foragers and the earliest food-producing groups in the region. Preliminary analyses show morphological parallels with individuals from the Horn of Africa and Central Africa, despite the geographical and chronological distances, suggesting similarly complex and dynamic demographic trajectories across northeastern African regions and adjacent areas.

Importantly, the identification of a group of individuals, mostly Early Holocene Middle Nile Valley foragers and Mid-Holocene individuals from the Horn of Africa, exhibiting unusual (syndromic) inner ear morphologies and rare congenital pathological changes, is compatible with multiple demographic scenarios, including but not limited to periods of reduced connectivity and/or population structuring under climatic constraints. These patterns are not only consistent with archaeological and environmental evidence from both regions but may also reflect broader processes of population sub-structuring across parts of prehistoric northeastern Africa in relation to past climatic constraints.

## Methods

### Skeletal material

The bony labyrinths of 148 individuals were included in this study (with 46 individuals presenting both left and right antimeres, i.e., *n* = 194 bony labyrinths in total). These originate from various archaeological skeletal assemblages from Eurasia and Africa, as well as present-day South Africa (Fig. 1, Table 1).

Many of the individuals included in this study (*n* = 68) were sampled from Late Pleistocene to Mid-Holocene sites in the Middle Nile Valley region (i.e., present-day Sudan and southern Egypt). Petrous bones from contemporaneous assemblages in the Horn of Africa (*n* = 10) and from Central Africa (*n* = 7) were also included in this work to evaluate regional morphological affinities and contextualise Middle Nile Valley variation within a broader (northeastern) African framework. These individuals were also selected and considered to provide preliminary insights into the population history of both regions, also depending on the availability and/or accessibility of skeletal material for each region.

Other comparative samples from Africa include the Late Pleistocene remains from Nazlet Khater 2 (Egypt), Ishango 37 (Democratic Republic of the Congo), Guli Waabayo (Somalia), and Hofmeyr (South Africa). These were selected to represent key temporal and geographic points across the continent, providing a broader context for interpreting variation in northeastern Africa. We also included a recent South African sample (Pretoria Bone Collection, *n* = 30 individuals) to provide a baseline of recent *Homo sapiens*, population-specific range of variation.

Comparative samples, contemporaneous to some of the oldest African specimens in our sample, include French and Belgian late Neandertals (*n* = 11) and European Upper Palaeolithic *Homo sapiens* (*n* = 8), as well as older Neandertal specimens (i.e., dated to MIS 6-5; *n* = 10). Additional comparative data from Pleistocene Eurasian fossils were also gathered from the literature^8,40–44,89–91^ and included in the analyses of 2D measurements solely. These were incorporated for broader comparison and (for Neandertals) as an outgroup, helping to polarise variation and identify traits specific to *Homo sapiens*, as well as particular morphologies.

These individuals were included in all protocols detailed in this work, except for two individuals for whom the cochlea was not preserved or was too damaged. These were removed from the 2D and/or landmark-based analyses (Supplementary Data 1). See Supplementary Data 1 for further details on the material included in this study.

### Ethics statement

Part of the material included in this study was excavated in the framework of several international archaeological missions in Africa, with the authorisation and support of the responsible authorities and governments. The collections analysed here derive from diverse excavation and curatorial histories, which are summarised in Supplementary Note ﻿S2, including details on the circumstances of recovery, exportation, study, and current curation. Some of the material recovered from these excavations, including human remains studied in this work, is currently on temporary loan or permanent deposit in various European and American institutions for study, documentation and restoration, with all necessary licences and permits issued by the responsible authorities and governments. Access to the collections was granted by the institutions responsible for the remains, and all inquiries regarding access to the original material should be directed to them.

We recognise that archaeological collections from Africa are embedded within broader historical and structural contexts that have shaped patterns of excavation, curation, access, and participation in research. While the present study was conducted under formal authorisations and institutional approvals, and in accordance with applicable regulations, access to heritage collections and research infrastructure remains uneven globally. By documenting the provenance, excavation histories, and current status of the collections, we aim to contribute to clear reporting of the governance and stewardship contexts in which this research was conducted, and to support transparent and responsible research practices that acknowledge these broader structural considerations.

Access to the µCT acquisitions from the Pretoria Bone Collection was granted by the University of Pretoria, with approval of the Research Ethics Committee of the Faculty of Health Sciences (HUM017/1123).

### Scanning procedure and segmentation

Temporal bones and crania with preserved bony labyrinths were selected for µCT acquisitions with the following parameter ranges: 83–280 µA intensity; 100–180 kV voltage; a 0.1–1.6 mm copper, or 1.0 mm aluminium, or copper + aluminium, or 0.5 mm titanium filter (see Supplementary Data 2 for sample-specific parameters). The final volumes were reconstructed with an isotropic voxel size ranging from 17.0 µm to 127.7 µm, depending on the acquisition parameters and size of the skeletal piece scanned. While this represents some variability, it falls within the range commonly reported in studies of bony labyrinth morphology^36,38,95,96^. Previous work^36^ has shown that variation in scan resolution within this range does not significantly affect the characterisation of labyrinthine shape, even when comparing substantially coarser resolutions. See Supplementary Data 2 for more details on each sample acquisition parameter, scan resolution, device and reconstruction software for the µCT acquisitions carried out for this work.

Given the differential state of preservation of the remains included in this study (e.g., bony labyrinth often filled with sediment; see Supplementary Table S5), segmentation of the reconstructed images was performed using Avizo 9.5.0 (Thermo Fisher Scientific) and Biomedisa software^92,93^. For each specimen, approximately a dozen slices were pre-segmented in two perpendicular orientations within Avizo. The *Smart interpolation* function in Biomedisa was then used to reconstruct the complete 3D volume of the bony labyrinth. Subsequently, all segmentations were manually inspected in Avizo to verify the accuracy of the semi-automatic segmentation process, with manual corrections applied as needed (e.g., to segment missing parts). All bony labyrinth surfaces were then extracted and computed using a constrained smoothing and a 0.4 padding value^94^. All segmentation and subsequent measurements were performed by N.M.

To assess segmentation reliability, four individuals representing varied taphonomic preservation (Supplementary Table S5) were segmented twice more by N.M., with a week between each digitisation to ensure independence. Analysis of the resulting 3D models revealed no significant differences between segmentation iterations, confirming the consistency of the procedure (Supplementary Fig. S15).

### 2D measurement protocol

A set of linear and angular measurements was taken to account for the overall shape of the bony labyrinth^38,89^. Prior to this, each stack of images was reoriented into the transverse and sagittal planes using the *Resample Transformed Image* function in Avizo 9.5.0, with the “standard” and “extended” interpolation options. The “standard” interpolation performs linear interpolation between neighbouring voxels (i.e., trilinear interpolation in 3D), providing a smooth transformation of the image stack without introducing significant artefacts. For this, a slice was manually oriented in the transverse plane following the orientations described in refs. ^38,89^, roughly aligning with the lateral semi-circular canal (SCC). The entire image stack was then reoriented according to this reference slice. Next, another slice was generated perpendicular to the defined transverse plane and manually aligned in the sagittal orientation described in refs. ^38,89^, with particular attention to correctly positioning it at the level of the common crus (Supplementary Fig. S16). Finally, the image stack was resampled based on this orientation. All subsequent measurements were performed on these two reoriented planes.

The linear measurements recorded include the height and width of each SCC (e.g., ASCh; ASCw) and of the cochlea (i.e., COh; COw). Based on these, we calculated the radius of curvature of each SCC and of the cochlear basal turn (e.g., ASC-R), as well as the relative radius of curvature of the canals (e.g., ASCh-%R). We also computed the sagittal labyrinthine index (SLI) and two angles (LSCm<APA and LSCm<COs). These measurements were chosen to best describe the overall morphology of the bony labyrinth and to allow comparisons with the largest comparative dataset possible of previously published inner ear measurements from Africa, Europe and the Levant^8,40–44,89–91^.

### Landmark-based analysis

All right bony labyrinth surfaces were mirrored to allow comparison between left and right antimeres. Using the *B-spline* function in Avizo 9.5.0, a total of 8 landmarks and 180 semi-landmarks were manually digitised by N.M. on these meshes (Supplementary Fig. S17 and Table S5). This template is inspired by Gunz et al.’s^95^ landmarking procedure for the SCCs and by Beaudet’s^96^ approach for the cochlea. For the SCCs, this consists of computing the midline skeleton of the segmented volumes, using the *Auto Skeleton* function in Avizo 9.5.0 and the protocol described by Gunz et al^95^. Landmarks were placed at the entry point of each canal and of the common crus into the vestibule, and at the Y-junction of the common crus. Along the midlines, 38 equidistant semi-landmarks were resampled on each SCC and 8 on the common crus. For the cochlea, we followed Beaudet^96^ and digitised 48 equidistant semi-landmarks along the outer midline of the structure, and two fixed landmarks at the apex of the cupula and at the entry point of the cochlea into the vestibule.

Landmarks and semi-landmarks coordinates were then imported in R (v4.2.2; R Core Team 2022)^97^ and the *shapes* v.1.2.7^98^ and *Morpho* v2.1^99^ were used for data pre-processing (i.e., Generalised Procrustes Analysis). Semi-landmarks were not slid. Instead, the *B-spline* curves generated in Avizo during landmark digitisation were exported to R, where all curve points were resampled at equal arc-length spacing using the *resampleCurve()* function in *Morpho*, providing consistent point ordering across individuals. Sliding is typically recommended to reduce visualisation artefacts in thin-plate spline (TPS) warping and to accommodate specimens with missing or incomplete curves^95^. In the present study, however, no TPS deformation grids were used, and all specimens preserved complete and uninterrupted canal and cochlear curves, for which Gunz et al. ^95^ note that the differences between analysing unslid equidistant points and slid semi-landmarks are “negligible”. Given the fully preserved curves and the absence of missing data, we follow previous inner ear studies that rely on equidistant curve resampling without sliding^96,100^.

To account for potential intra-observer error, eight bony labyrinths were randomly selected, and the landmarks and semi-landmarks were digitised again after several months. Comparison of the original and control Procrustes coordinates (Supplementary Fig. S18) shows good repeatability of the protocol.

### Diffeomorphic surface matching (DSM)

Contrary to the landmark-based approach, which relies on the 3D coordinates of a set of points, the DSM method is based on the mathematical principle of diffeomorphism and relies on the computation of a sample-average surface model and its deformations to each of the investigated surfaces^101–103^. In other words, it does not focus on a point-to-point correspondence, as in landmark-based methods, but on the distances and local orientations between two meshes, which allows comparison of entire surfaces rather than a pre-defined number and ordering of points.

Prior to the DSM analyses, all bony labyrinth surfaces were pre-processed. This consisted of separating the vestibule and SCCs from the cochlea, following Urciuoli et al.’s protocol^37,104,105^. After this, all individual meshes were decimated to 30,000 triangles^37,104,105^, manually oriented and then aligned and rigidly scaled using an Iterative Closest Points (ICP) algorithm through the *Align Surface* function on Avizo. Finally, the meshes were imported into Deformetrica v.4.3.0 (https://www.deformetrica.org/)^101,106^ to compute the sample average shape, called global mean shape (GMS). With this software, we also computed the deformations between the GMS and each individual mesh, summarised by sets of control points associated with vector fields (called momenta). These shape parameters (*n*_*parameters*_ = 2800) were then imported into R v.4.2.2, using the *RToolsForDeformetrica* package^107^.

To assess the potential effect of including a much larger, more recent sample in such analyses, especially on the computation of momenta, we reran the calculation of the GMS and the subsequent GMS-to-individuals deformation by excluding recent South African individuals from the dataset. The obtained PCA (Supplementary Fig. S19) showed little to no differences with that obtained from the original momenta. A Mantel test comparing the Euclidean inter-individual distance matrices derived from the two PCAs revealed an almost perfect correlation (*r* = 0.990, *p* = 0.0001; 9,999 permutations), indicating that the overall morphometric structure is nearly identical between the two computational approaches.

### Statistical analyses

All statistical analyses were computed in R. With the *ade4* v.1.7.22^108–110^ packages, we computed principal component analyses (PCA): for the GMS-to-individual diffeomorphisms and Procrustes coordinates, we used the covariance matrices, and for the 2D measurements, we used the correlation matrix. For readability issues, the PCA plots displayed in Fig. 3 only show the convex hulls and centroid points of the different groups considered in this study, with only individual values shown for the relevant specimen. Figures showing the full individual distribution are shown in the Supplementary Fig. S3. Interactive HTML versions of all PCA plots presented in this work are also available in Supplementary Data 3. These were generated using the *plotly* v4.11.0^111^ package in R. To assess the significance of principal components in the high-dimensional DSM dataset (2800 variables, *n* = 194), we applied the *getMeaningfulPCs()* function from *Morpho* v2.1. Only PC1 exceeded the eigenvalue noise floor, as expected in high-dimensional morphometrics. PC2 was retained for visualisation and ordination because it captures biologically coherent variation, reflects trends observed in landmark and 2D datasets, and highlights secondary axes of morphospace variation relevant to geographic and taxonomic patterns. PC1 is treated as the dominant statistical signal, while PC2 is interpreted heuristically. Similarly, PCs 3–4, presented in the Supplementary Fig. S2, are reported for reference.

Allometry was tested using linear regressions of PC scores against the natural logarithm of either the centroid size of the SCCs and vestibule for the DSM analysis or the whole bony labyrinth for the landmark-based analyses (Supplementary Table S1). To test the impact of including Neandertal individuals in the analyses, this was carried out for both the whole sample and a *Homo sapiens*-only sample. Centroid size of the SCCs and vestibule was estimated based on the decimated meshes, prior to their scaling and alignment, using the *cSize* function of *Morpho*. Linear regressions indicate that size has a generally little to no effect on PC scores. While some regressions are statistically significant, the associated R² values are low, indicating that size explains only a minor proportion of the morphological variation captured by the PCs.

With the *ade4* package, we computed between-group principal component analyses (bgPCA) on the Middle Nile Valley sample, using all PCs and the individuals from five a priori defined groups: Late Palaeolithic (LP), Mesolithic (Meso), Nubian Early Neolithic (ENeoN), Central Sudanese Early Neolithic (ENeoCS), and Nubian Middle Neolithic (MNeoN). This was done to classify the individuals from the Western Desert and Shaqadud, which were projected *a posteriori* onto the bgPCA morphospace and for which typicality probabilities were computed (Supplementary Fig. S4 and Table S3). Cross-validated bgPCA computed on the same dataset showed a consistent distribution pattern, suggesting no spurious group separation effects^112^ (Supplementary Fig. S20).

We also performed permutational multivariate analyses of variance (PERMANOVA), using the *pairwiseAdonis* v.0.4.1 package^113^ (1000 permutations; based on Euclidean distance and with no correction^114^), to assess the structure of data and results, and to estimate the morphological distances between the a priori defined groups from the Middle Nile Valley and their significance (Supplementary Table S2).

Mahalanobis distances and disparity metrics were calculated using the first 18 principal components of the DSM analysis (of the *Homo sapiens*-only sample), which each explained more than 1% of the total variance and together accounted for 79.98% of the total variance^115^. Disparity was estimated using the *dispRity* function from the *dispRity* v.1.8 package^116^, applied to bootstrapped group distributions (20,000 replicates) to account for small sample sizes.

As detailed in Supplementary Data 1, both left and right bony labyrinths were included, when available in the dataset, and considered as separate observations in the analyses. This applies to all present-day South African individuals and several archaeological specimens. Including both antimeres allowed the capture of biologically relevant asymmetry in labyrinth morphology and preserved unilateral morphological features, including atypical/outlier morphologies. To document the extent of bilateral variation, a supplementary PCA figure (Supplementary Fig. S21) connects left and right labyrinths from the same individual, allowing assessment of within-individual variation relative to between-individual variation. Although directionality is not strictly uniform, many left-right displacements show a general trend along the lower-left to upper-right quadrant of the PCA, which can be consistent with variation in the thickness of the semi-circular canals. More generally, these displacements appear small relative to general morphospace variation and are consistent with overall limited asymmetry.

### Recording of pathological changes

The re-oriented scans (i.e., transverse and sagittal views produced for the 2D measurement analysis) of all individuals were assessed for pathological evidence. Fistulas were recorded as present or absent by visual examination of both transverse and sagittal images for every individual. Suspected fistulas were required to be consistently visible across consecutive slices and in both anatomical planes, and particular attention was paid to edge morphology, with genuine features expected to present smooth and well-defined boundaries. The size of the vestibular aqueduct was measured following medical standard protocol^66^. This consisted of measuring the width of the aqueduct at the operculum and at the mid-point (halfway between the operculum and the vestibule), in a transverse (or ‘axial’) view. Standard clinical thresholds of ≥2 mm (at the operculum) and ≥1 mm (at the mid-point) were taken to describe enlarged vestibular aqueducts^66^. The cochlear nerve canal (CNC) diameter was measured in a transverse/axial view as the width of the bony canal at the entry of the cochlea, with a standard clinical threshold of ≤1.8 mm used to describe a stenotic CNC^68^. To account for taphonomic variation, the preservation state of each specimen was evaluated before scoring (see Supplementary Table S5 for detailed taphonomic information and Supplementary Fig. S22 for representative examples). When taphonomic alteration prevented reliable measurement or observation of a feature, the individual was scored as “non-observable” (occurring rarely, primarily affecting VA measurements at the operculum). Finally, as the identification of these pathological traits is based on criteria derived from clinical imaging protocols, which are typically established on medical CT data with coarser resolution than the µCT scans used here, the level of variation in voxel size in our dataset is unlikely to affect the identification of the anatomical features considered.

### Reporting summary

Further information on research design is available in the Nature Portfolio Reporting Summary linked to this article.

## Supplementary information

Supplementary Information

Descriptions of Additional Supplementary Files

Supplementary Dataset 1-2,4-5

Supplementary Dataset 3

Supplementary Code 1

Reporting Summary

Transparent Peer Review file

## Source data

Source Data

## Data Availability

Access to the original material should be addressed to the curators of the relevant curating institutions (see Supplementary Note S2). Access is subject to institutional approval and curation policies governing the human remains. The µCT scan data and derived 3D models are available from the corresponding authors only with permission from the relevant curating institutions. Raw µCT data are also held by the curating institutions and may be accessed under their data access procedures for scientific research purposes. Requests must be directed to the curatorial contacts listed in Supplementary Note S2 and are subject to review and approval by the relevant institution. Linear measurements and landmark coordinate data for the Middle Nile Valley and present-day South African individuals are provided in Supplementary Data 4. Equivalent datasets for other individuals are available from the corresponding publications cited in the text (i.e., refs. ^8,40–44,89–91^) or from the corresponding author subject to institutional approval. Source data are provided with this paper.

## Code availability

The R scripts used in this study, adapted from previously published sources cited in the manuscript, are available as Supplementary Code 1. The scripts were developed for the specific datasets analysed here and may require adaptation to be reused. Some of the raw data required to run the scripts are not yet publicly available (i.e., data with restricted curatorial access mentioned in the Data Availability statement).

## References

[1] Grine, F. E. The Late Quaternary hominins of Africa: The skeletal evidence from MIS 6-2. in *Africa from MIS 6-2* (eds. Jones, S. C. & Stewart, B. A.), 323–381 (Springer, 2016).

[2] Kuper, R. & Kröpelin, S. Climate-controlled Holocene occupation in the Sahara: motor of Africa’s evolution. *Science* **313**, 803–807 (2006). 16857900 10.1126/science.1130989

[3] Pausata, F. S. R. et al. The greening of the Sahara: past changes and future implications. *One Earth* **2**, 235–250 (2020).

[4] Hoelzmann, P., Keding, B., Berke, H., Kröpelin, S. & Kruse, H.-J. Environmental change and archaeology: lake evolution and human occupation in the Eastern Sahara during the Holocene. *Palaeogeogr. Palaeoclimatol. Palaeoecol.* **169**, 193–217 (2001).

[5] Leplongeon, A. The main Nile Valley at the end of the Pleistocene (28–15 ka): Dispersal corridor or environmental refugium? *Front. Earth Sci.* **8**, 607183 (2021).

[6] Gasse, F. Hydrological changes in the African tropics since the last glacial maximum. *Quat. Sci. Rev.* **19**, 189–211 (2000).

[7] Mirazón Lahr, M. & Foley, R. A. Human evolution in Late Quaternary Eastern Africa. in *Africa from MIS 6-2* (eds. Jones, S. C. & Stewart, B. A.), 215–231 (Springer, 2016).

[8] Crevecoeur, I. et al. Human evolution and population dynamics in Northeast Africa at the end of the Pleistocene and the beginning of the Holocene. In *From the Big Dry to the Holocene in Eastern Africa and beyond*, Actes de la séance de la Société préhistorique française de Toulouse (septembre 2019) (eds. Lesur, J. et al.), 13–38 (Société 746 Préhistorique française, Paris, 2023).

[9] Sirak, K., Sawchuk, E. & Prendergast, M. Ancient Human DNA and African Population History. (Oxford Research Encyclopedia of Anthropology, 2022)

[10] Lipson, M. et al. Ancient West African foragers in the context of African population history. *Nature* **577**, 665–670 (2020). 31969706 10.1038/s41586-020-1929-1 PMC8386425

[11] Salem, N. et al. Ancient DNA from the Green Sahara reveals ancestral North African lineage. *Nature* **641**, 144–150 (2025). 40175549 10.1038/s41586-025-08793-7 PMC12043513

[12] Prendergast, M. E. et al. Ancient DNA reveals a multistep spread of the first herders into sub-Saharan Africa. *Science* **365**, eaaw6275 (2019). 31147405 10.1126/science.aaw6275 PMC6827346

[13] Morez Jacobs, A. et al. Whole-genome ancestry of an Old Kingdom Egyptian. *Nature* **644**, 714–721 (2025). 10.1038/s41586-025-09195-5 PMC12367555 40604286

[14] Irish, J. D. & Usai, D. The transition from hunting–gathering to agriculture in Nubia: dental evidence for and against selection, population continuity and discontinuity. *Proc. R. Soc. B Biol. Sci.* **288**, 20210969 (2021). 10.1098/rspb.2021.0969 PMC8187995 34102887

[15] Martin, N. et al. From hunter-gatherers to food producers: new dental insights into the Nile Valley population history (Late Paleolithic–Neolithic). *Am. J. Biol. Anthropol.* **184**, e24948 (2024). 38733278 10.1002/ajpa.24948

[16] Martin, N. et al. Enamel–dentine junction morphology reveals population replacement and mobility in the late prehistoric Middle Nile Valley. *Proc. Natl. Acad. Sci. USA* **122**, e2419122122 (2025). 40163762 10.1073/pnas.2419122122 PMC12012513

[17] Irish, J. D. Population continuity vs. discontinuity revisited: dental affinities among late Paleolithic through Christian-era Nubians. *Am. J. Phys. Anthropol.* **128**, 520–535 (2005). 15895433 10.1002/ajpa.20109

[18] Irish, J. D. The mean measure of divergence: its utility in model-free and model-bound analyses relative to the Mahalanobis *D*^2^ distance for nonmetric traits. *Am. J. Hum. Biol.* **22**, 378–395 (2010). 19918917 10.1002/ajhb.21010

[19] Benoiston, A.-S., Bayle, P. & Crevecoeur, I. Biological affinity of the Mesolithic and Neolithic populations from El-Barga, Sudan: The dental remains. in *Nubian archaeology in the XXIst century (1st-6th September 2014)* (ed. Honegger, M.), 805–817 (Peeters publishers, 2018).

[20] Gatto, M. C. & Zerboni, A. Holocene supra-regional environmental changes as trigger for major socio-cultural processes in Northeastern Africa and the Sahara. *Afr. Archaeol. Rev.* **32**, 301–333 (2015).

[21] Mologni, C. et al. Holocene East African monsoonal variations recorded in wave-dominated clastic paleo-shorelines of Lake Abhe, Central Afar region (Ethiopia & Djibouti). *Geomorphology* **391**, 107896 (2021).

[22] Khalidi, L. et al. 9000 years of human lakeside adaptation in the Ethiopian Afar: Fisher-foragers and the first pastoralists in the Lake Abhe basin during the African Humid Period. *Quat. Sci. Rev*. **243**, 106459 (2020).

[23] Marshall, M. H. et al. Late Pleistocene and Holocene drought events at Lake Tana, the source of the Blue Nile. *Glob. Planet. Change* **78**, 147–161 (2011).

[24] Marshall, M. H. et al. Climatic change in northern Ethiopia during the past 17,000 years: a diatom and stable isotope record from Lake Ashenge. *Palaeogeogr. Palaeoclimatol. Palaeoecol.* **279**, 114–127 (2009).

[25] Khalidi, L. et al. Late prehistoric oasitic niches along the Southern Red Sea (Yemen and Horn of Africa). in *From refugia to oases: living in arid environments from prehistoric times to the present day* (eds. Purdue, L., Charbonnier, J. & Khalidi, L.), 71–99 (Éditions APDCA, 2018).

[26] Beyin, A., Prendergast, M. E., Grillo, K. M. & Wang, H. New radiocarbon dates for terminal Pleistocene and early Holocene settlements in West Turkana, northern Kenya. *Quat. Sci. Rev.* **168**, 208–215 (2017).

[27] Le Meillour, L. et al. The name of the game: palaeoproteomics and radiocarbon dates further refine the presence and dispersal of caprines in eastern and southern Africa. *R. Soc. Open Sci.* **10**, 231002 (2023). 38026023 10.1098/rsos.231002 PMC10663795

[28] Grillo, K. M., McKeeby, Z. & Hildebrand, E. A. “Nderit Ware” and the origins of pastoralist pottery in eastern Africa. *Quat. Int.* **608**, 226–242 (2022).

[29] Marshall, F. & Hildebrand, E. A. Cattle before crops: the beginnings of food production in Africa. *J. World Prehist.* **16**, 99–143 (2002).

[30] Sawchuk, E. A., Goldstein, S. T., Grillo, K. M. & Hildebrand, E. A. Cemeteries on a moving frontier: Mortuary practices and the spread of pastoralism from the Sahara into eastern Africa. *J. Anthropol. Archaeol.* **51**, 187–205 (2018).

[31] Gutherz, X. et al. New insights on the first Neolithic societies in the Horn of Africa: The site of Wakrita, Djibouti. *J. Field Archaeol.* **40**, 55–68 (2015).

[32] Cauliez, J., Dachy, T. & Gutherz, X. Les premières sociétés de production en Afrique. Xe millénaire avant notre ère - Ier millénaire de notre ère. in *De l’Acacus au Zimbabwe: 20,000 avant notre ère – XVIIe siècle* (ed. Fauvelle, F. X.), 469–498 (Belin, 2018).

[33] Coudert, L., Lesur, J., Bruxelles, L., Gutherz, X. & Cauliez, J. New archaeozoological results from Asa Koma (Djibouti): Contributing to the understanding of faunal exploitation during the 3rd millennium BC in the Horn of Africa. *Quat. Int.* **471**, 219–228 (2018).

[34] Gutherz, X. Quel Néolithique dans la Corne de l’Afrique? *Archéo-Nil.* **23**, 73–90 (2013).

[35] Ponce de León, M. S. et al. Human bony labyrinth is an indicator of population history and dispersal from Africa. *Proc. Natl. Acad. Sci. USA* **115**, 4128–4133 (2018). 29610337 10.1073/pnas.1717873115 PMC5910833

[36] Urciuoli, A. et al. Semicircular canals shed light on bottleneck events in the evolution of the Neanderthal clade. *Nat. Commun.* **16**, 972 (2025). 39979299 10.1038/s41467-025-56155-8 PMC11842635

[37] Urciuoli, A. et al. The evolution of the vestibular apparatus in apes and humans. *eLife* **9**, e51261 (2020). 32122463 10.7554/eLife.51261 PMC7054002

[38] Spoor, F. & Zonneveld, F. Morphometry of the primate bony labyrinth: a new method based on high-resolution computed tomography. *J. Anat.* **186**, 271–286 (1995). 7649826 PMC1167185

[39] Mennecart, B. et al. Ruminant inner ear shape records 35 million years of neutral evolution. *Nat. Commun.* **13**, 7222 (2022). 36473836 10.1038/s41467-022-34656-0 PMC9726890

[40] Crevecoeur, I., Brooks, A., Ribot, I., Cornelissen, E. & Semal, P. Late Stone Age human remains from Ishango (Democratic Republic of Congo): new insights on Late Pleistocene modern human diversity in Africa. *J. Hum. Evol.* **96**, 35–57 (2016). 27343771 10.1016/j.jhevol.2016.04.003

[41] Crevecoeur, I. et al. The Hofmeyr Bony Labyrinth: Morphological Description and Affinity. in *Hofmeyr* (ed. Grine, F. E.), 165–178 (Springer International Publishing, 2022).

[42] Bouchneb, L. & Crevecoeur, I. The inner ear of Nazlet Khater 2 (Upper Paleolithic, Egypt). *J. Hum. Evol.* **56**, 257–262 (2009). 19144388 10.1016/j.jhevol.2008.12.003

[43] Quam, R., Lorenzo, C., Martínez, I., Gracia-Téllez, A. & Arsuaga, J. L. The bony labyrinth of the middle Pleistocene Sima de los Huesos hominins (Sierra de Atapuerca, Spain). *J. Hum. Evol.* **90**, 1–15 (2016). 26767955 10.1016/j.jhevol.2015.09.007

[44] Hublin, J.-J., Spoor, F., Braun, M., Zonneveld, F. & Condemi, S. A late Neanderthal associated with Upper Palaeolithic artefacts. *Nature* **381**, 224–226 (1996). 8622762 10.1038/381224a0

[45] Velez, A. D. et al. Geometric morphometric analysis of the bony labyrinth of the Sima de los Huesos hominins. *J. Hum. Evol.* **174**, 103280 (2023). 36455404 10.1016/j.jhevol.2022.103280

[46] Hill, C. A., Radovčić, J. & Frayer, D. W. Brief communication: investigation of the semicircular canal variation in the Krapina Neandertals. *Am. J. Phys. Anthropol.* **154**, 302–306 (2014). 24610299 10.1002/ajpa.22506

[47] Zhang, Y., Urciuoli, A., Zanolli, C., Kullmer, O. & Wu, X. Three-dimensional geometric morphometric analysis of the bony labyrinth of Xujiayao 6. *J. Hum. Evol.* **189**, 103514 (2024). 38547737 10.1016/j.jhevol.2024.103514

[48] Vermeersch, P. M. et al. Early and Middle Holocene human occupation of the Egyptian Eastern Desert: Sodmein Cave. *Afr. Archaeol. Rev.* **32**, 465–503 (2015).

[49] Xia, X. et al. Global dispersal and adaptive evolution of domestic cattle: a genomic perspective. *Stress Biol.* **3**, 8 (2023). 37676580 10.1007/s44154-023-00085-2 PMC10441868

[50] Schuck, W. Steinzeitliche Gräber im Wadi Shaw (Nordsudan). in *Tides of the Desert: Contributions To The Archaeology And Environmental History of Africa in Honour of Rudolph Kuper* (ed. Jennerstrasse 8), 239–255 (Heinrich-Barth-Institut, 2002).

[51] Hublin, J.-J. et al. New fossils from Jebel Irhoud, Morocco and the pan-African origin of Homo sapiens. *Nature* **546**, 289–292 (2017). 28593953 10.1038/nature22336

[52] Gunz, P. et al. Early modern human diversity suggests subdivided population structure and a complex out-of-Africa scenario. *Proc. Natl. Acad. Sci. USA* **106**, 6094–6098 (2009). 19307568 10.1073/pnas.0808160106 PMC2669363

[53] Ribot, I., Orban, R. & Maret, P. de. *The Prehistoric Burials of Shum Laka Rockshelter (North-West Cameroon)*. 164, 218p. (Annales Sciences Humaines/Annalen Menswetenschappen, Musée Royal de l’Afrique Centrale/Koninklijk Museum voor Midden-Afrika, 2001).

[54] Prüfer, K. et al. A genome sequence from a modern human skull over 45,000 years old from Zlatý kůň in Czechia. *Nat. Ecol. Evol.* **5**, 820–825 (2021). 33828249 10.1038/s41559-021-01443-x PMC8175239

[55] Sümer, A. P. et al. Earliest modern human genomes constrain timing of Neanderthal admixture. *Nature* **638**, 711–717 (2025). 39667410 10.1038/s41586-024-08420-x PMC11839475

[56] Rathmann, H. et al. Human population dynamics in Upper Paleolithic Europe inferred from fossil dental phenotypes. *Sci. Adv.* **10**, eadn8129 (2024). 39151011 10.1126/sciadv.adn8129 PMC11328903

[57] Conde-Valverde, M. et al. The child who lived: Down syndrome among Neanderthals? *Sci. Adv.* **10**, eadn9310 (2024). 38924400 10.1126/sciadv.adn9310 PMC11204207

[58] Purcell, D., Johnson, J., Fischbein, N. & Lalwani, A. K. Establishment of normative cochlear and vestibular measurements to aid in the diagnosis of inner ear malformations. *Otolaryngol. Neck Surg.* **128**, 78–87 (2003). 10.1067/mhn.2003.51 12574763

[59] Lan, M.-Y., Shiao, J.-Y., Ho, C.-Y. & Hung, H.-C. Measurements of normal inner ear on computed tomography in children with congenital sensorineural hearing loss. *Eur. Arch. Otorhinolaryngol.* **266**, 1361–1364 (2009). 19238416 10.1007/s00405-009-0923-x

[60] Crevecoeur, I. First anthropological insights on the Early Holocene funerary assemblages from El-Barga. in *Kerma, Documents de la mission archéologique suisse au Soudan*, vol. 4, 19–28 (Université de Neuchâtel, 2012).

[61] Brukner et al. Palaeodemography and palaeopathology of Khartoum Mesolithic skeletal remains from Jebel Sabaloka in central Sudan: first insights from the site of Sphinx. *J. Natl. Mus. Prague Nat. Hist. Ser*. **191**, 65–82 (2022).

[62] Varadzinová, L., Varadzin, L., Crevecoeur, I., Kapustka, K. & McCool, J.-P. Excavations at the prehistoric site of Fox Hill in the western part of Jebel Sabaloka (2017–2018). *Sudan & Nubia* **26**, 160–181 (2022).

[63] Varadzinová, L., Varadzin, L., Crevecoeur, I., Martin, N. & Hassan, S. Exploration of the late prehistoric occupation in the western part of Jebel Sabaloka in central Sudan: findings of the 2022 field campaign. *Prague Egyptol. Stud.* **33**, 28–53 (2024).

[64] Duday, H. et al. *As Ela (District de Dikhil) - Sites de Wakrita*, *Ali Daba 10 et Hara Ide 3. Rapport de Mission*. (2006).

[65] Duday, H. et al. *As Ela (District de Dikhil) - Sites d’Hara Ide 2 et 3 et d’Ali Daba 2 à 12*. *Rapport de Mission*. (2004).

[66] Boston, M. et al. The large vestibular aqueduct: a new definition based on audiologic and computed tomography correlation. *Otolaryngol. Neck Surg.* **136**, 972–977 (2007). 10.1016/j.otohns.2006.12.011 17547990

[67] Suzuki, M., Ota, Y., Tanaka, T. & Ota, Y. Lateral semicircular canal–enlarged vestibular aqueduct fistula associated with paroxysmal positional nystagmus. *Otol. Neurotol.* **37**, e192–e193 (2016). 25973841 10.1097/MAO.0000000000000778

[68] Teissier, N., Van Den Abbeele, T., Sebag, G. & Elmaleh-Berges, M. Computed tomography measurements of the normal and the pathologic cochlea in children. *Pediatr. Radiol.* **40**, 275–283 (2010). 20012953 10.1007/s00247-009-1423-2

[69] Rosito, L. P. S. et al. Cholesteatoma labyrinthine fistula: prevalence and impact. *Braz. J. Otorhinolaryngol.* **85**, 222–227 (2019). 29599061 10.1016/j.bjorl.2018.01.005 PMC9452264

[70] Sheehy, J. L. & Brackmann, D. E. Cholesteatoma surgery: management of the labyrinthine fistula—A report of 97 cases. * Laryngoscope* **89**, 78–87 (1979). 423655 10.1288/00005537-197901000-00008

[71] Jackler, R. K. & De La Cruz, A. The large vestibular aqueduct syndrome. * Laryngoscope* **99**, 1238–1243 (1989). 2601537 10.1288/00005537-198912000-00006

[72] Valvassori, G. E. & Clemis, J. D. The large vestibular aqueduct syndrome. * Laryngoscope* **88**, 723–728 (1978). 306012 10.1002/lary.1978.88.5.723

[73] Berrettini, S. et al. Large vestibular aqueduct syndrome: audiological, radiological, clinical, and genetic features. *Am. J. Otolaryngol.* **26**, 363–371 (2005). 16275403 10.1016/j.amjoto.2005.02.013

[74] Elmaleh-Bergès, M. et al. Spectrum of temporal bone abnormalities in patients with Waardenburg syndrome and *SOX10* mutations. *Am. J. Neuroradiol.* **34**, 1257–1263 (2013). 23237859 10.3174/ajnr.A3367 PMC7964579

[75] Feraco, P., Piccinini, S. & Gagliardo, C. Imaging of inner ear malformations: a primer for radiologists. *Radiol. Med. (Torino)* **126**, 1282–1295 (2021). 34196909 10.1007/s11547-021-01387-z PMC8520521

[76] Traylor, K. S., Dodson, S., Kralik, S. F., Ho, C. & Radhakrishnan, R. Inner ear anomalies in congenital hearing loss: Imaging, genetics, and associated syndromes. *Neurographics* **9**, 358–372 (2019).

[77] Koch, B., Goold, A., Egelhoff, J. & Benton, C. Partial absence of the posterior semicircular canal in Alagille syndrome: CT findings. *Pediatr. Radiol.* **36**, 977–979 (2006). 16761118 10.1007/s00247-006-0230-2

[78] Haj Kassem, L., Ahmado, M. F. & Sheikh Alganameh, M. A rare case of seven siblings with Waardenburg syndrome: a case report. *J. Med. Case Rep.* **12**, 192 (2018). 29973257 10.1186/s13256-018-1704-1 PMC6032534

[79] Wollnik, B. et al. Homozygous and heterozygous inheritance of *PAX3* mutations causes different types of Waardenburg syndrome. *Am. J. Med. Genet. A.* **122A**, 42–45 (2003). 12949970 10.1002/ajmg.a.20260

[80] Alfi, O. S., Chang, R. & Azen, S. P. Evidence for genetic control of nondisjunction in man. *Am. J. Hum. Genet.* **32**, 477–483 (1980). 6446853 PMC1686121

[81] Ray, A. et al. Risk of Down syndrome birth: consanguineous marriage is associated with maternal meiosis-II nondisjunction at younger age and without any detectable recombination error. *Am. J. Med. Genet. A.* **176**, 2342–2349 (2018). 30240118 10.1002/ajmg.a.40511

[82] Charfeddine, I. et al. Pendred syndrome in Tunisia. *Eur. Ann. Otorhinolaryngol. Head. Neck Dis.* **127**, 7–10 (2010). 20822748 10.1016/j.anorl.2010.02.002

[83] Lofrano-Porto, A. et al. Pendred Syndrome in a large consanguineous Brazilian family caused by a homozygous mutation in the SLC26A4 gene. *Arq. Bras. Endocrinol. Metabol.* **52**, 1296–1303 (2008). 19169484 10.1590/s0004-27302008000800015

[84] Honegger, M. El-Barga: un site clé pour la compréhension du Mésolithique et du début du Néolithique en Nubie. *Rev. Paléobiol. S* **10**, 95–104 (2005).

[85] Honegger, M. & Williams, M. Human occupations and environmental changes in the Nile valley during the Holocene: the case of Kerma in Upper Nubia (northern Sudan). *Quat. Sci. Rev.* **130**, 141–154 (2015).

[86] Osypiński, P. et al. Unearthing a Middle Nile crossroads – exploring the prehistory of the Letti Basin (Sudan). *Pol. Archaeol. Mediterr.* **31**, 25–60 (2022).

[87] Salvatori, S. & Usai, D. The Mesolithic and Neolithic in Sudan. in *Handbook of Ancient Nubia* (ed. Raue, D.), 171–194 (De Gruyter, 2019).

[88] Brukner Havelková, P. et al. Patterns of Violence in the Pre-Neolithic Nile Valley. *Afr. Archaeol. Rev.* **40**, 597–619 (2023).

[89] Spoor, F., Hublin, J.-J., Braun, M. & Zonneveld, F. The bony labyrinth of Neanderthals. *J. Hum. Evol.* **44**, 141–165 (2003). 12662940 10.1016/s0047-2484(02)00166-5

[90] Conde-Valverde, M. et al. The bony labyrinth in the Aroeira 3 Middle Pleistocene cranium. *J. Hum. Evol.* **124**, 105–116 (2018). 30201119 10.1016/j.jhevol.2018.08.003

[91] Quam, R., Martinez, I. & Arsuaga, J. L. New observations on the human fossils from Petit-Puymoyen (Charente). *PaleoAnthropology* **96**, 95–105 (2011).

[92] Lösel, P. et al. Introducing Biomedisa as an open-source online platform for biomedical image segmentation. *Nat. Commun.* **11**, 5577 (2020). 33149150 10.1038/s41467-020-19303-w PMC7642381

[93] Lösel, P. & Heuveline, V. Enhancing a diffusion algorithm for 4D image segmentation using local information. In (eds. Styner, M. A. & Angelini, E. D.) *Medical Imaging 2016: Image Processing*, 97842L (SPIE, 2016).

[94] Kupczik, K. & Hublin, J.-J. Mandibular molar root morphology in Neanderthals and Late Pleistocene and recent *Homo sapiens*. *J. Hum. Evol.* **5**, 525–541 (2010). 10.1016/j.jhevol.2010.05.009 20719359

[95] Gunz, P., Ramsier, M., Kuhrig, M., Hublin, J. & Spoor, F. The mammalian bony labyrinth reconsidered, introducing a comprehensive geometric morphometric approach. *J. Anat.* **220**, 529–543 (2012). 22404255 10.1111/j.1469-7580.2012.01493.x PMC3390507

[96] Beaudet, A. The inner ear of the *Paranthropus* specimen DNH 22 from Drimolen, South Africa. *Am. J. Phys. Anthropol.* **170**, 439–446 (2019). 31290572 10.1002/ajpa.23901

[97] R. Core Team. R: a language and environment for statistical computing. R Foundation for Statistical Computing (R CoreTeam, 2022).

[98] Dryden, I. L. *Shapes: Statistical Shape Analysis* (Academic Press, 2003).

[99] Schlager, S. Morpho and Rvcg - Shape analysis in R. in *Statistical shape and deformation analysis* (eds. Zheng, G., Li, S. & Szekely, G.), 217–256 (Academic Press, 2017).

[100] Beaudet, A. et al. Upper third molar internal structural organization and semicircular canal morphology in Plio-Pleistocene South African cercopithecoids. *J. Hum. Evol.* **95**, 104–120 (2016). 27260177 10.1016/j.jhevol.2016.04.004

[101] Durrleman, S. et al. Morphometry of anatomical shape complexes with dense deformations and sparse parameters. *NeuroImage* **101**, 35–49 (2014). 24973601 10.1016/j.neuroimage.2014.06.043 PMC4871626

[102] Durrleman, S., Pennec, X., Trouvé, A., Ayache, N. & Braga, J. Comparison of the endocranial ontogenies between chimpanzees and bonobos via temporal regression and spatiotemporal registration. *J. Hum. Evol.* **62**, 74–88 (2012). 22137587 10.1016/j.jhevol.2011.10.004

[103] Beaudet, A. et al. Morphoarchitectural variation in South African fossil cercopithecoid endocasts. *J. Hum. Evol.* **101**, 65–78 (2016). 27886811 10.1016/j.jhevol.2016.09.003

[104] Urciuoli, A. et al. A comparative analysis of the vestibular apparatus in Epipliopithecus vindobonensis: Phylogenetic implications. *J. Hum. Evol.* **151**, 102930 (2021). 33422741 10.1016/j.jhevol.2020.102930

[105] Urciuoli, A. et al. Reassessment of the phylogenetic relationships of the late Miocene apes *Hispanopithecus* and *Rudapithecus* based on vestibular morphology. *Proc. Natl. Acad. Sci. USA* **118**, e2015215118 (2021). 33495351 10.1073/pnas.2015215118 PMC7865142

[106] Bône, A., Louis, M., Martin, B. & Durrleman, S. Deformetrica 4: An open-source software for statistical shape analysis. In *Shape in medical imaging. Shape MI 2018* (eds. Reuter, M. et al.), 3–13 (Springer International Publishing, Cham, 2018).

[107] Dumoncel, J. *RToolsForDeformetrica: R script for Deformetrica ouput*. (Gitlab, 2020).

[108] Bougeard, S. & Dray, S. Supervised multiblock analysis in *R* with the ade4 package. *J. Stat. Softw.* **86**, 1–7 (2018).

[109] Dray, S. & Dufour, A.-B. The ade4 package: implementing the duality diagram for ecologists. *J. Stat. Softw.* **22**, 1–20 (2007).

[110] Thioulouse, J. et al. *Multivariate Analysis of Ecological Data with Ade4*. (Springer, 2018).

[111] Sievert, C. *Interactive web-based data visualization with R, plotly, and shiny*. (CRC Press, 2020).

[112] Cardini, A. & Polly, D. Cross-validated between group PCA scatterplots: a solution to spurious group separation? *Evol. Biologigy* **47**, 85–95 (2020).

[113] Martinez Arbizu, P. *pairwiseAdonis: Pairwise multilevel comparison using Adonis*. (2017).

[114] Rothman, K. J. No adjustments are needed for multiple comparisons. *Epidemiology* **1**, 43–46 (1990). 2081237

[115] Reyes-Centeno, H., Harvati, K. & Jäger, G. Tracking modern human population history from linguistic and cranial phenotype. *Sci. Rep.* **6**, 36645 (2016). 27833101 10.1038/srep36645 PMC5105118

[116] Guillerme, T. DispRity: a modular R package for measuring disparity. *Methods Ecol. Evol.* **9**, 1755–1763 (2018).

[117] Anderson, J. E. Late Paleolithic skeletal remains from Nubia. in *Prehistory of Nubia* (ed. Wendorf, F.), 996–1040 (Southern Methodist University Press, 1968).

[118] Wendorf, F. Site 117: a Nubian final Paleolithic graveyard near Jebel Sahaba, Sudan. In *Prehistory of Nubia* (ed. Wendorf, F.) 954–995 (Southern Methodist University Press, 1968).

[119] Zazzo, A. Bone and enamel carbonate diagenesis: a radiocarbon prospective. *Palaeogeogr. Palaeoclimatol. Palaeoecol.* **416**, 168–178 (2014).

[120] Varadzinová, L., Varadzin, L., Brukner Havelková, P., Crevecoeur, I. & Garcea, E. A. A. Archaeology of Holocene hunter-gatherers at the sixth Nile cataract, central Sudan. *Bull. d'Archéologie Maroc.* **27**, 103–120 (2022).

[121] Varadzinová, L., Varadzin, L. & Ambrose, S. H. New radiocarbon dates for postglacial reoccupation of the Sudanese Nile. *Quat. Sci. Rev.* **303**, 107953 (2023).

[122] Irish, J. D. & De Groote, I. The human skeletal remains from Ghaba: a physical anthropological assessment. in *Ghaba: an early Neolithic cemetery in Central Sudan* (eds. Salvatori, S., Usai, D. & Lecointe, Y.), vol. 1, 85–108 (Afrika Magna, 2016).

[123] Salvatori, S., Usai, D. & Lecointe, Y. (eds.) *Ghaba: an early Neolithic cemetery in Central Sudan*. (Africa Magna, 2016).

[124] Salvatori, S., Usai, D. & Lecointe, Y. The discovery and excavation of the cemetery. In *Ghaba: an early Neolithic cemetery in Central Sudan* (eds. Salvatori, S., Usai, D. & Lecointe, Y.), vol. 1, 3–9 (Africa Magna, 2016).

[125] Reinold, J. Kadruka and the Neolithic in the Northern Dongola Reach. *Sudan & Nubia* **5**, 2–10 (2001).

[126] Reinold, J. Le cimetière néolithique KDK.1 de Kadruka (Nubie soudanaise): Premiers résultats et essai de corrélation avec les sites du Soudan central. in *Actes du VIIe Congrès international d’études nubiennes* (ed. Bonnet, C.), vol. 2, 93–100 (Etudes Nubiennes, 2000).

[127] Reinold, J. Les cimetières préhistoriques au Soudan: Coutumes funéraires et systèmes sociaux. in *Acta Nubica: Proceedings of the 10th International Conference of Nubian Studies* (eds. Caneva, I. & Roccati, A.), 13–162 (Istituto Poligrafico e Zecca dello Stato, 2006).

[128] Jesse, F. Cattle, sherds and mighty walls – The Wadi Howar from Neolithic to Kushite times. *Sudan & Nubia* **10**, 43–54 (2006).

[129] Jesse, F. & Keding, B. Death in the desert - Burials in the Wadi Howar region (Eastern Sahara). In *Tides of the desert: contributions to the archaeology and environmental history of Africa in honour to Rudolph Kuper* (ed. Jennerstrasse 8), 277–293 (Heinrich-Barth-Institut, 2002).

[130] Becker, E. The prehistoric inhabitants of the Wadi Howar: an anthropological study of human skeletal remains from the Sudanese part of the Eastern Sahara. (Johannes Gutenberg-Universität Mainz, 2011).

[131] Varadzin, L. et al. The *Shaqadud Archaeological Project* (Sudan): exploring prehistoric cultural adaptations in the Sahelian hinterlands. *Antiquity* **97**, e2 (2023).

[132] Duday, H., et al. Les pratiques funéraires des populations du Gobaad du IVe millénaire avant notre ère à l’islamisation dans leur contexte régional de la Corne de l’Afrique. In X. Gutherz (Ed.), *Asa Koma, site Néolithique dans le bassin du Gobaad (République de Djibouti)* (pp. 241–264). (Presses universitaires de la Méditerranée, 2017).

[133] Brandt, S. A. The Upper Pleistocene and early Holocene prehistory of the Horn of Africa. *Afr. Archaeol. Rev.* **4**, 41–82 (1986).

[134] Brandt, S. A. Early Holocene mortuary practices and hunter-gatherer adaptations in southern Somalia. *World Archaeol.* **20**, 40–56 (1988). 16470993 10.1080/00438243.1988.9980055

[135] Angel, J. L., Phenice, T. W., Robbins, L. H. & Lynch, M. *Late Stone-Age fishermen of Lothagam, Kenya*. (Publications of the Museum – Michigan State University, 1980).

[136] L’Abbé, E. N., Krüger, G. C., Theye, C. E. G., Hagg, A. C. & Sapo, O. The Pretoria Bone Collection: A 21st century skeletal collection in South Africa. *Forensic Sci.* **1**, 220–227 (2021).

[137] Vermeersch, P. M. *Palaeolithic quarrying sites in Upper and Middle Egypt*. (Leuven University Press, 2002).

[138] Vermeersch, P. M. et al. 33,000-yr old chert mining site and related Homo in the Egyptian Nile Valley. *Nature* **309**, 342–344 (1984). 6427628 10.1038/309342a0

[139] Grine, F. (ed.) *Hofmeyr: A Late Pleistocene human skull from South Africa*. (Springer 2022).

[140] Clark, J. D. *The prehistoric cultures of the Horn of Africa. An analysis of the Stone Age cultural and climatic succession in the Somalilands and Eastern parts of Abyssinia*. (Michigan University Press, 1954).

[141] Vandermeersch, B. *Les hommes fossiles de Qafzeh (Israël)*. Editions du Centre national de la recherche scientifique, Paris (1981).

[142] McDermott, F. et al. Mass-spectrometric U-series dates for Israeli Neanderthal/early modern hominid sites. *Nature* **363**, 252–255 (1993). 8387643 10.1038/363252a0

[143] Henry-Gambier, D., Nespoulet, R. & Chiotti, L. An Early Gravettian cultural attribution for the human fossils from the Cro-Magnon rock shelter (Les Eyzies-de-Tayac, Dordogne). *PALEO* **24**, 121–138 (2013).

[144] Villotte, S., Chiotti, L., Nespoulet, R. & Henry-Gambier, D. É tude anthropologique des vestiges humains récemment découverts issus de la couche 2 de l’abri Pataud (Les Eyzies-de-Tayac-Sireuil, Dordogne, France). *Bull. mém. Soc. Anthr. Paris* **27**, 158–188 (2015).

[145] Soubeyran, M. *Station de Raymonden à Chancelade (Dordogne). Série Paléolithiques Conservées Au Musée Du Périgord, à Périgueux (Dordogne)*. (Ecole du Louvre, Paris, 1965).

[146] Gambier, D., Valladas, H., Tisnérat-Laborde, N., Arnold, M. & Bresson, F. Datation de vestiges humains présumés du Paléolithique supérieur par la méthode du Carbone 14 en spectrométrie de masse par accélérateur. *PALEO* **12**, 201–212 (2000).

[147] Ferembach, D. Le squelette humain Azilien de Rochereil (Dordogne). *Bull. Mém. Soc. Anthrop. Paris* **2**, 271–291 (1974).

[148] Manzi, G., Gracia, A. & Arsuaga, J. L. Cranial discrete traits in the Middle Pleistocene humans from Sima de los Huesos (Sierra de Atapuerca, Spain). Does hypostosis represent any increase in “ontogenetic stress” along the Neanderthal lineage? *J. Hum. Evol.* **38**, 425–446 (2000). 10683308 10.1006/jhev.1999.0362

[149] Arnold, L. J. et al. Luminescence dating and palaeomagnetic age constraint on hominins from Sima de los Huesos, Atapuerca, Spain. *J. Hum. Evol.* **67**, 85–107 (2014). 24485349 10.1016/j.jhevol.2013.12.001

[150] Rink, W. J., Schwarcz, H. P., Smith, F. H. & Radovĉiĉ, J. ESR ages for Krapina hominids. *Nature* **378**, 24–24 (1995). 7477281 10.1038/378024a0

[151] Eixea, A., Oltra, I., Bergadà, M. M. & Villaverde, V. The reinterpretation of the Cova Negra archaeological and stratigraphical sequence and its implications in the understanding of the Middle Palaeolithic Iberian Peninsula. *Quat. Int.* **566**, 98–112 (2020).

[152] Richard, M. et al. ESR/U-series chronology of early Neanderthal occupations at Cova Negra (Valencia, Spain). *Quat. Geochronol.* **49**, 283–290 (2019).

[153] Rougier, H. & Semal, P. (eds.) *Spy Cave: 125 Years of Multidisciplinary Research at the Betch Aux Rotches (Jemeppe-Sur-Sambre, Province of Namur, Belgium)*, vol. 1. (Anthropologica et Praehistorica 123/2012, Royal Belgian Institute of Natural Sciences, Royal Belgian Society of Anthropology and Praehistory & NESPOS Society, 2013).

[154] Maureille, B. La redécouverte du nouveau-né néandertalien Le Moustier 2. *PALEO* **14**, 221–238 (2002).

[155] Maureille, B. et al. Les Pradelles à Marilac-le-Franc (Charente). Fouilles 2001-2007: nouveaux résultats et synthèse. in *Préhistoire entre Vienne et Charente. Hommes et Sociétés du Paléolithique* (eds. Buisson-Catil, J. & Primault, J.) vol. XXXVIII, 145–162 (Chauvigny, 2010).

[156] Garralda, M. D. et al. The Neanderthal teeth from Marillac (Charente, Southwestern France): Morphology, comparisons and paleobiology. *J. Hum. Evol.* **138**, 102683 (2020). 31765984 10.1016/j.jhevol.2019.102683

[157] Mussini, C. *Les restes humains moustériens des Pradelles (Marillac-le-Franc, Charente, France): étude morphométrique et réflexions sur un aspect comportemental des Néandertaliens*. (University of Bordeaux, 2011).

[158] Rougier, H. et al. Neandertal cannibalism and Neandertal bones used as tools in Northern Europe. *Sci. Rep.* **6**, 29005 (2016). 27381450 10.1038/srep29005 PMC4933918

[159] Soressi, M., Jones, H., Rink, W., Maureille, B. & Tillier, A. The Pech-de-l’Azé I Neandertal child: ESR, uranium-series, and AMS 14C dating of its MTA type B context. *J. Hum. Evol.* **52**, 455–466 (2007). 17284331 10.1016/j.jhevol.2006.11.006

[160] Frouin, M. et al. Dating the Middle Paleolithic deposits of La Quina Amont (Charente, France) using luminescence methods. *J. Hum. Evol.* **109**, 30–45 (2017). 28688458 10.1016/j.jhevol.2017.05.002

[161] Guérin, G. et al. A multi-method luminescence dating of the Palaeolithic sequence of La Ferrassie based on new excavations adjacent to the La Ferrassie 1 and 2 skeletons. *J. Archaeol. Sci.* **58**, 147–166 (2015).

[162] Balzeau, A. et al. Pluridisciplinary evidence for burial for the La Ferrassie 8 Neandertal child. *Sci. Rep.* **10**, 21230 (2020). 33299013 10.1038/s41598-020-77611-z PMC7725784

[163] Gómez-Olivencia, A., Crevecoeur, I. & Balzeau, A. La Ferrassie 8 Neandertal child reloaded: New remains and re-assessment of the original collection. *J. Hum. Evol.* **82**, 107–126 (2015). 25805043 10.1016/j.jhevol.2015.02.008

[164] Dibble, H. L. et al. A critical look at evidence from La Chapelle-aux-Saints supporting an intentional Neandertal burial. *J. Archaeol. Sci.* **53**, 649–657 (2015).

[165] Rendu, W. et al. Evidence supporting an intentional Neandertal burial at La Chapelle-aux-Saints. *Proc. Natl. Acad. Sci. USA* **111**, 81–86 (2014). 24344286 10.1073/pnas.1316780110 PMC3890882

[166] Deviese, T. et al. Reevaluating the timing of Neanderthal disappearance in Northwest Europe. *Proc. Natl. Acad. Sci. USA* **118**, e2022466118 (2021). 33798098 10.1073/pnas.2022466118 PMC7999949

[167] Spoor, F., Hublin, J.-J., Kondo, O. The bony labyrinth of the Dederiyeh child. In: Akazawa, T., Muhesen, S. (Eds.), Neanderthal burials: excavations of the Dederiyeh Cave, Afrin, Syria., pp. 215e220 (KW Publications, 2002).

[168] Valladas, H. et al. Thermoluminescence dates for the Neanderthal burial site at Kebara in Israel. *Nature* **330**, 159–160 (1987).

[169] Valladas, H. et al. TL Dates for the Neanderthal Site of the Amud Cave, Israel. *J. Archaeol. Sci.* **26**, 259–268 (1999).

