# Supplementary material for: Bony labyrinth morphology reveals deep population history and isolation among Homo sapiens in prehistoric northeastern Africa (44–3 ka)
Source: Nat Commun. 2026 Aug 12;17:8181. doi: 10.1038/s41467-026-76423-5 (PMC13470358; doi:10.1038/s41467-026-76423-5)
Supplement: Supplementary file 1 [file 41467_2026_76423_MOESM1_ESM.pdf]

Supplementary material for

Bony labyrinth morphology reveals deep population history and isolation among *Homo sapiens* in prehistoric northeastern Africa (44–3 ka)

Nicolas Martin*, Petr Velemínský, Alessandro Urciuoli, Lenka Varadzinová, Matthieu Honegger, Stanley H. Ambrose, Daniel Antoine, Steven A. Brandt, Jaroslav Brůžek, Jessie Cauliez, Henri Duday, Frederick Grine, Petra Brukner Havelková, Joel D. Irish, Friederike Jesse, Laura Maréchal, Bruno Maureille, Isabelle Ribot, Rebeka Rmoutilova, Hélène Rougier, Frédéric Santos, Adrien Thibeault, Donatella Usai, Nicolas Vanderesse, Ladislav Varadzin, Rebecca J. Whiting and Isabelle Crevecoeur*

*Corresponding authors:

Nicolas Martin

UMR 5199 PACEA, Université de Bordeaux,
Bâtiment B8, Allée Geoffroy Saint Hilaire, CS 50023
33615 Pessac Cedex – France

Phone : +33540003733
Email: nicolas.martin.bioanth@gmail.com

Isabelle Crevecoeur

UMR 5199 PACEA, Université de Bordeaux,
Bâtiment B8, Allée Geoffroy Saint Hilaire, CS 50023
33615 Pessac Cedex – France

Phone : +33540003342
Email: isabelle.crevecoeur@u-bordeaux.fr

This file includes:

SI Note S1-S3

SI Figs. S1-S22

SI Table S1-S6

SI References

Supplementary Note S1: Description of the principal component analyses (PCAs) performed on the landmarks Procrustes coordinates and 2D measurements.

2D measurement (2D) analysis

The 2D analysis included a broader range of individuals and samples than the other approaches, notably incorporating older representatives of the Neandertal clade (Pre-Neandertals) as well as early *Homo sapiens* from Qafzeh and Skhul (Main text, Fig. 3B). The PCA based on these linear measurements and angular parameters reveals morphometric variation between the Neandertal clade and *Homo sapiens* samples along PC1, which accounts for 22.5% of the total variance. The negative values of this component appear to reflect the Neandertal group's relatively larger lateral semi-circular canal (higher LSC%R and LSC-R values), narrower anterior SCC (higher ASCh/w values), and smaller, more inferiorly positioned posterior SCC (higher SLI and lower PSC-R and PSC%R values).

Within the *Homo sapiens* sample, PC1 also captures notable variation between Eurasian and African Late Pleistocene individuals from the Middle Nile Valley food producers. The latter group is characterised by high positive values along PC1, indicating a larger posterior SCC, relatively smaller anterior and lateral SCCs, a slightly larger cochlea, and a distinct orientation of the cochlea relative to the lateral SCC (LSCm>COs).

Interestingly, consistent with the other analyses, a subgroup of Holocene individuals emerges as outliers. These individuals plot within the range of variation observed in Neandertals. One individual (AD10_S1) even plots beyond the range of variation of Pre-Neandertals, characterised by extremely low values on both PCs. This pattern is associated with a smaller posterior SCC, smaller anterior SCC, slightly reduced cochlear size (CO-R), and different orientation of the cochlea or common crus relative to the lateral SCC (LSCm>COs and LCSm>APA).

Landmark-based (LM) analysis

The PCA conducted for the LM analysis reveals significant variation between the groups examined in this study (Main text, Fig. 3C). Most notably, Neandertal individuals are found within the upper-right quadrant of the PCA plot, due to their characteristically smaller and lower-positioned posterior semi-circular canal (SCC), smaller anterior SCC, slightly larger and wider lateral SCC (i.e., highly positive values along PC2), and cochlea with fewer turns (i.e., highly negative values along PC1).

Among *Homo sapiens* individuals, the greatest variation is observed along PC1 (accounting for 39.55% of the total variance), primarily reflecting differences in the number of cochlear turns. In contrast, PC2 (accounting for 10.81% of the variance) distinguishes more effectively between the groups under comparison. Specifically, Middle Nile Valley food-producing populations are characterised by lower values along this component, whereas African and European Late Pleistocene individuals plot closer to zero or at higher PC2 values. This results from the Mid-

Holocene individuals having a larger and rounder posterior SCC and relatively smaller anterior and lateral SCCs.

Interestingly, as observed in the DSM analysis (Main text, Fig. 3A), a subset of individuals consistently emerges as outliers due to their markedly reduced posterior SCC, shorter common crus, and slightly smaller anterior SCC. While some of these individuals plot closer to the Neandertal range of variation, most exhibit highly positive values on PC2, which correlates with a more tightly coiled cochlea.

Supplementary Note S2: Detailed excavation background and collection status of the original African skeletal assemblages included in this study.

Abu Tabari (Sudan)

The sites of Abu Tabari were excavated in 2002, 2003 and 2006, in the framework of the CRC ACACIA (*Arid Climate Adaptation and Cultural Innovation in Africa*), Sub-project A2 "Wadi Howar: Settlement Area and Thoroughfare at the Southern Margin of the Libyan Desert (North-western Sudan)" of the University of Cologne (Germany). Excavations at Abu Tabari were carried out with all necessary permits issued by the National Corporation for Antiquities and Museums (NCAM) of Sudan and with the participation of Muawiya Ali El-Tayeb (2002-2003) and Ali Elmirghani Mohamed (2006) as inspectors from the NCAM.

The archaeological material from these excavations was exported on loan for study and analysis with all necessary licenses and permits issued by the NCAM of Sudan. The human remains are now curated at the Duckworth Laboratory (University of Cambridge, UK). Study of the collections was done in collaboration with European institutions, including Johannes Gutenberg University Mainz, Ludwig-Maximilian-University Munich (both Germany) and PALAEO centre (University of York, UK). Requests for access to the original material should be directly addressed to the Duckworth Laboratory curatorial staff (contact: duckworth@human-evol.cam.ac.uk).

Ali Daba (Djibouti)

The site of Ali Daba was excavated between 1999 and 2003, in the framework of the French-Djiboutian Archaeological Mission (French Ministry for Europe and Foreign Affairs) led by H. Duday and X. Gutherz. Excavations at Ali Daba were carried out with all necessary permits issued by the Djiboutian Centre for Research and Studies and the Institute of Archaeological and Historical Research (Ministry of Higher Education and Research of Djibouti).

Part of the collections excavated from the site was exported to University Toulouse Jean-Jaurès (France) and the University of Bordeaux (France) on temporary loan, for curation, restoration, and study. Export was carried with all necessary licenses and permits issued by the Djiboutian Centre for Research and Studies and the Institute of Archaeological and Historical Research. Study of the collections was carried in collaboration with European and African institutions, including the Institute of Archaeological and Historical Research (IRAH) of Djibouti and the TRACES Laboratory (France). Requests for access to the original material should be directly addressed to the Director of the Institute of Archaeological and Historical Research of Djibouti (S. Zakaria: zakariacheik@yahoo.fr).

El-Barga (Sudan)

The site of El-Barga was excavated between 2001 and 2014, in the framework of the Swiss Archaeological mission in Sudan led by M. Honegger. Excavations at El-Barga were carried out with all necessary permits issued by the NCAM of Sudan and with the participation of NCAM representatives, and curators from the Kerma museum, as well as the active involvement of the local communities.

Part of the collections excavated from the site were exported to the University of Neuchâtel on temporary loan for curation, restoration, and study. Export was carried with all necessary licenses and permits issued by the NCAM of Sudan. Study of the collections was carried in collaboration with European, African, North American, and Australian institutions, including the University of Neuchâtel, the Kerma museum, the University of Adelaide, the University of Leuven, and the University of Bordeaux. Requests for access to the original material should be directly addressed to the University of Neuchâtel (M. Honegger, project director: matthieu.honegger@unine.ch).

Fox Hill (Sudan)

The site of Fox Hill was excavated between 2011 and 2022, in the framework of the Sabaloka (West Bank) Research Project (Charles University, Prague) led by L. Varadzinová. Excavations were carried out with all necessary permits issued by the NCAM of Sudan and with the participation of Murtada Bushara, Seif ed-Daula, Sayda Ahmed Adam, Balsam Abdelhamed, Bushara Abdallah Adam (in 2019) and Saddam Hassan as duly appointed representatives of the NCAM, Altayeb Abdallah from the University of Al Neelain as a field assistant (in 2019), four junior staff from the NCAM (Abir Yahya Adam, Selma Mohamed Omar, Maydulin Osman, Ehssan Hashim), and twelve students from the University of Khartoum (Hamad Mohamed Hamdeen, Mohamed Al Badri Suleyman, Ammar Awad Mohamed, Ezz Aldein Abdelrahim, Hajir Fadlallah Marhoum, Tasneen Abdurahman Mahjoub Elhaj, Nahid Adam Hdob Suliman), University of Al Neelain (Altayeb Abdallah – in 2017 and 2018; Mohamed el-Tahir Suleyman, Rowida Rasheda Abdelqadar, Hajir Fadlallah Marhoum), University of Shendi (Bushara Abdallah Adam) and University of Bahri (Safaa Musa Eisa) for archaeological training.

Part of the human remains excavated at the site were exported to the Czech Republic on long-term loan for curation, restoration, and study. They are currently deposited in the National Museum. Export was carried with all necessary licenses and permits issued by the NCAM of Sudan. Study of the assemblage was carried in collaboration with European and North American institutions, including the University of Bordeaux (France), and Department of Anthropology, University of Illinois at Urbana-Champaign (USA). Requests for access to the original material should be directly addressed to the Charles University, Faculty of Arts, Czech Institute of Egyptology (L. Varadzinová, project director: lenka.Varadzinova@ff.cuni.cz).

Ghaba (Sudan)

The site of Ghaba was excavated between 1980 and 1986, in the framework of the French Archaeological mission in Sudan led by the *Section Française de la Direction des Antiquités du Soudan* (SFDAS). Excavations at Ghaba were carried out with all necessary permits issued by the NCAM of Sudan and with the participation of members of the NCAM (namely, Salah Omer Es Sadiq, Gamal el-Din Mohammed Idris, Awadallah el-Bacha, El Sheikh Mahmoud el Tayed, and Mohamed Taha Mohamed Ali).

Part of the collections excavated from the site were permanently exported to the Liverpool John Moores University (LJMU, UK) for curation, restoration, and study. Export was carried with all necessary licenses and permits issued by the NCAM of Sudan and the SFDAS. Study of the collections was carried in collaboration with European institutions, including Liverpool John Moores University, the *Centro Studi Sudanesi and Sub-Sahariani*, and Pompeu Fabra University of Barcelona. Requests for access to the original material should be directly addressed to the LJMU (J. D. Irish, contact: J.D.Irish@ljmu.ac.uk).

Gogoshiis Qabe (Somalia)

The site of Gogoshiis Qabe was excavated in 1935 and between 1983 and 1989, respectively in the framework of the Italian Ethnological mission in Somalia (dir. P. Graziosi; University of Florence) and of the Buur Ecological and Archeological Project (dir. S. Brandt; University of Florida). Excavations at Gogoshiis Qabe were carried out with all necessary permits issued by the Somali Academy of Sciences and Arts and with the support of the Buur Heybe's local communities.

Part of the collections excavated from the site were exported on loan to the University of Florida (USA) for curation, restoration, and study. Export was carried with all necessary licenses and permits issued by Somali Academy of Sciences and Arts. Study of the collections was carried in collaboration with European and North American institutions. Requests for access to the original material should be directly addressed to University of Florida (S. Brandt, project director: sbrandt@ufl.edu).

Guli Waabayo (Somalia)

The site of Guli Waabayo was excavated between1985 and 1989, in the framework of the the Buur Ecological and Archeological Project led by S. Brandt. Excavations at of Guli Waabayo were carried out with all necessary permits issued by Somali Academy of Sciences and Arts and with the support of the Buur Heybe's local communities.

Part of the collections excavated from the site were exported on loan to the University of Florida (USA) for curation, restoration, and study. Export was carried with all necessary licenses and permits issued by Somali Academy of Sciences and Arts. Study of the collections was carried

in collaboration with European and North American institutions. Requests for access to the original material should be directly addressed to University of Florida (S. Brandt, project director: sbrandt@ufl.edu).

Hara Idé 2 (Djibouti)

The site of Hara Idé 2 was excavated between 2001 and 2003, in the framework of the French-Djiboutian Archaeological (French Ministry for Europe and Foreign Affairs) led by H. Duday and X. Gutherz. Excavations at Hara Idé 2 were carried out with all necessary permits issued by the Djiboutian Centre for Research and Studies and the Institute of Archaeological and Historical Research (Ministry of Higher Education and Research of Djibouti).

Part of the collections excavated from the site was exported to University Toulouse Jean-Jaurès (France) and the University of Bordeaux (France) on temporary loan, for curation, restoration, and study. Export was carried with all necessary licenses and permits issued by the Djiboutian Centre for Research and Studies and the Institute of Archaeological and Historical Research. Study of the collections was carried in collaboration with European and African institutions, including the Institute of Archaeological and Historical Research (IRAH) of Djibouti and the TRACES Laboratory (France). Requests for access to the original material should be directly addressed to the Director of the Institute of Archaeological and Historical Research of Djibouti (S. Zakaria: zakariacheik@yahoo.fr).

Jebel Sahaba (Sudan)

The site of Jebel Sahaba was excavated between 1965 and 1966, in the framework of the Combined Prehistoric Expedition during the UNESCO 'International Campaign to Save the Monuments of Nubia' and carried out under the direction of F. Wendorf (Southern Methodist University, USA). Excavations at Jebel Sahaba were carried out with all necessary permits issued by Sudan's Antiquities Service. This took place with the participation of UNESCO staff in Sudan and Egypt, the Director General and staff of the Antiquities Service Department in Egypt, as well as the Commissioner and Inspectors of the Sudan Antiquities Service. Furthermore, many members of the local communities and students and staff from the University of Cairo were involved with the excavations, the workmen at Jebel Sahaba were led by Aïd Maariif and came from the Maariff family of the Awazin tribe. Many other organisations and their staff were heavily involved in this work, including from the USA: The State Department, National Science Foundation, Fort Burgwin Research Centre, The Smithsonian Institution; from Poland: The Academy of Sciences; from Egypt: the Egyptian Geological Survey; from Belgium: the Fonds National de la Recherche Scientifique; and from France: the Centre National de la Recherche Scientifique. For further details about individuals see *Prehistory of Arid North Africa. Essays in Honor of Fred Wendorf* (A. Close, 1987).

Part of the collections excavated from the site, including the human remains, were exported to the Southern Methodist University (SMU) after the excavations. SMU acquired the material legally, through division, as part of the UNESCO Campaign to Save the Monuments of Nubia. Since 2001, these remains are now on permanent deposition at the British Museum for curation, restoration, and study. The British Museum legally acquired the collection as a gift from Department of Anthropology, SMU, on 25 August 2001. This was signed by the Provost and Vice President for Academic Affairs and had been approved by the Anthropology Collections Advisory Committee and by the Department of Anthropology. Export was carried out with all necessary licenses and permits issued by the NCAM of Sudan. Study of these collections has been carried out in collaboration with European, North American and African institutions, including with colleagues from the NCAM of Sudan, and students studying across the world. Requests for access to these remains should be directly addressed to the British Museum. Since the donation of the remains from Jebel Sahaba, and other donations of human remains from NCAM over the last 15-20 years, the British Museum has been capacity building in Sudan, training new bioarchaeologists and supporting the building of new bioarchaeological facilities, so as future excavated remains may be stored and studied in Sudan.

Requests for access to the original material should be directly addressed to the Department of Egypt and Sudan of the British Museum (D. Antoine, curator: DAntoine@britishmuseum.org).

Kadruka 1 (Sudan)

The site of Kadruka 1 was excavated between 1986 and 1999, in the framework of the French Archaeological mission in Sudan, and led by the SFDAS (dir. J. Reinold). Excavations at Kadruka 1 were carried out with all necessary permits issued by the NCAM and with the participation of SFDAS and NCAM representatives, and with the support and assistance from the local communities.

Part of the collections excavated from the site were permanently exported to the University of Geneva for curation, restoration, and study. Export was carried with all necessary licenses and permits issued by the NCAM and the SFDAS. Study of the collections was carried in collaboration with European and Sudanese institutions. Requests for access to the original material should be directly addressed to the University of Geneva (J. Desideri, contact: Jocelyne.Desideri@unige.ch) and the SFDAS (contact: khartoum.sfdas@gmail.com).

Mille-Logya (Ethiopia)

The site of Mille-Logya was excavated in 2014, in the framework of the Mille-Logya Project led by Z. Alemseged. Excavations at Mille-Logya were carried out with all necessary permits

issued by the Authority for Research and Conservation of the Cultural Heritage (ARCCH) of Ethiopia and with the participation of S. Melaku as ARCCH's representative.

The collections excavated from the site is housed at the National Museum of Ethiopia. Export of one petrous bone to the Max Planck Institute (Jena) for microCT acquisition was carried with all necessary licenses and permits issued by the ARCCH of Ethiopia. Study of the collections is carried in collaboration with the National Museum of Ethiopia. Requests for access to the original material should be directly addressed to Dr. Z. Alemseged (alemseged@uchicago.edu) and to Sahle Melaku (sahle2131@gmail.com), responsible for the Paleontology and Paleoanthropology collections at the National Museum of Ethiopia.

Shaqadud (Sudan)

The site of Shaqadud was excavated in the 1960's, 1980's and between 2020 and 2022, in the framework of the K.H. Otto's investigations, the Butana Archaeological Project (dir. A.E. Marks and A. Mohammed-Ali), and the Shaqadud Archaeological Project (dir. L. Varadzin) respectively. In the framework of the latter project, which provided the samples for this study, excavations were carried out with all necessary permits issued by the NCAM of Sudan and with the participation of Bushara Abdallah Adam and Mustafa Abdalla Mohammed (NCAM), Altayeb Abdallah (University of Al Neelain), Ikram Madani Ahmed (University of Khartoum), and Hamad Mohamed Hamdeen (University of Al Neelain).

Part of the collections excavated from the site were exported to the Institute of Archaeology of the Czech Academy of Sciences, Prague (Czech Republic) on temporary loan for curation, restoration, and study. Export was carried with all necessary licenses and permits issued the NCAM of Sudan. Study of the collections was carried in collaboration with the University of Bordeaux (France) and the University of Illinois at Urbana-Champaign (USA). Requests for access to the original material should be directly addressed to the Institute of Archaeology of the Czech Academy of Sciences, Prague (L. Varadzin, project director: varadzin@arup.cas.cz).

Shum Laka (Cameroon)

After preliminary field work in the 1980's, the site of Shum Laka was re-excavated on a large-scale between1991 and 1994, in the framework of an interdisciplinary archaeological mission led by Pierre de Maret and Raymond Asombang. Excavations at the rock shelter of Shum Laka were carried out with all necessary research permits issued by Cameroonian Authorities (MESIRES) and with the participation of the following people: archaeologists from the AfricaMuseum in Tervuren (E. Cornelissen, P. Lavachery, J. Moeyersons, H. Doutrelepont); bioarchaeologists from the Royal Belgian Institute of Natural Sciences in Brussels (W. Van Neer, I. Ribot); palynologist from ORSTOM in Montpellier (J. Maley); students from Yaounde University (A. Dweazoni, E. Keumaleu) and Free University of Brussels (N. Collard, S. Collette, E. De Crits, S.

Fagnard, S. Fenaux, F. Renoirte, Z. Rosinsky) and Paris X (S. Boulay, C. Garreau). Both hospitality and help were also granted on-site by the Fon of Bafotchu-Mbu and its local population.

Part of the collections excavated from the site were exported to Royal Belgian Institute of Natural Sciences in Brussels on temporary loan for curation, restoration, and study. Export was carried with all necessary licenses and permits issued by the Cameroonian authorities. Study of the collections was carried in collaboration with the Yaounde University, the Free University of Brussels, the Royal Belgian Institute of Natural Sciences in Brussels, the AfricaMuseum, the Université de Montréal and the Boston Harvard Medical School. Requests for access to the original material should be directly addressed to the Royal Belgian Institute of Natural Sciences in Brussels (P. Semal, curator: psemal@naturalsciences.be).

Sphinx (Sudan)

The site of Sphinx was excavated between 2011 and 2019, in the framework of the Sabaloka (West Bank) Research Project (Charles University, Prague) led by L. Varadzinová. Excavations were carried out with all necessary permits issued by the NCAM of Sudan and with the participation of Murtada Bushara, Seif ed-Daula, Sarah Abdulatif esh-Sheikh and Bushara Abdallah Adam (in 2019) as duly appointed representatives of the NCAM, Altayeb Abdallah from the University of Al Neelain as a field assistant (in 2019), and seven junior staff from the NCAM (Abir Yahya Adam, Selma Mohamed Omar, Safaa Ahmed Mohamed, Reemah Abdelrahim Kabashi, Abud Adam Hamid, Bella Hajj Mohamed, Tafa'u Ráfi' Ahmed), and five students from the University of Khartoum (Hamad Mohamed Hamdeen, Mohamed Al Badri Suleyman) and the University of Bahri (Hanaa Mohamed Hafiz, Huyam Mohammed Alamin Ahmed Salim, Saba Suleiman Murkez) for archaeological training.

Part of the human remains excavated at the site were exported to the Czech Republic on long-term loan for curation, restoration, and study. They are currently deposited at the National Museum. Export was carried with all necessary licenses and permits issued by the NCAM of Sudan. Study of the assemblage was carried in collaboration with European and North American institutions, including the University of Bordeaux (France), and Department of Anthropology, University of Illinois at Urbana-Champaign (USA). Requests for access to the original material should be directly addressed to the Charles University, Faculty of Arts, Czech Institute of Egyptology (L. Varadzinová, project director: Lenka.Varadzinova@ff.cuni.cz).

Wadi Shaw (Sudan)

The site of Wadi Shaw 83/110 was excavated in 1983, in the framework of the B.O.S. (*Besiedlungsgeschichte der Ostsahara*)-Project led by R. Kuper (University of Cologne). Excavations at Wadi Shaw were carried out with all necessary permits issued by the NCAM of Sudan and with the participation of Jamal el Deen Idris (inspector from the NCAM).

The archaeological material from the excavation was exported on loan for study and analysis with all necessary licenses and permits issued by the NCAM of Sudan. The human remains are now curated at the Duckworth Laboratory (University of Cambridge, UK). Study of the collections was carried in collaboration with European institutions, including the University of Geneva (Switzerland), Johannes Gutenberg University Mainz (Germany) and PALAEO centre (University of York, UK). Requests for access to the original material should be directly addressed to the Duckworth Laboratory (University Cambridge, contact: duckworth@human-evol.cam.ac.uk).

Supplementary Note S3: Statistical comparison of EDJ- and bony labyrinth-based analyses of the Middle Nile Valley material.

To assess the capacity of each proxy—EDJ morphology of upper first and second molars (i.e., Martin et al., 2025) vs. bony labyrinth morphology—to discriminate among groups, we applied a common multivariate analytical framework to each dataset obtained for the Middle Nile Valley samples (**Supplementary Fig. S13**). Multivariate analyses were conducted separately for the UM1, UM2, and bony labyrinth datasets. For each dataset, principal component (PC) scores derived from precomputed ordinations were used as multivariate descriptors of the samples. All analyses were performed in R using the packages *caret* v. 7.0-1 (Kuhn et al., 2024), *vegan* v.2.7-2 (Oksanen et al., 2025), and *glmnet* v. 4.1-10 (Friedman et al., 2025; see **Supplementary Code**).

First, statistical differences in multivariate composition among groups were assessed using non-parametric multivariate analysis of variance (NPMANOVA) implemented with the *adonis2* function in the *vegan* package. Analyses were based on Euclidean distances calculated from the first two PC axes, with group as the explanatory factor. Statistical significance was evaluated using 9,999 permutations.

In addition to inferential testing, supervised classification was used to assess the ability of multivariate descriptors to predict group membership (i.e., the capacity of each analysis to discriminate between each group). Penalised multinomial logistic regression models were fitted using the *glmnet* method implemented in the *glmnet* package (see Friedman et al., 2010). Models were trained using all available variables and evaluated using leave-one-out cross-validation (LOOCV). This procedure was applied independently to each dataset to ensure comparability of model training and evaluation.

This approach was first applied to all five *a priori* defined chrono-cultural groups from the Middle Nile Valley (LP, Meso, ENeoN, ENeoCS, and MNeoN). Under this framework, classification rates were higher for the EDJ datasets (46% for UM1s and 40% for UM2s) than for the bony labyrinth dataset (18%). A subsequent analysis focusing on a simplified dichotomy between foragers and food producers (LP + Meso vs. ENeoN + ENeoCS + MNeoN) yielded substantially higher classification accuracies for the dental data, with 83% and 87% correct classification for UM1s and UM2s, respectively. In contrast, the bony labyrinth dataset achieved a classification accuracy of only 65%.

Together, these results indicate that both anatomical structures are more effective at differentiating between foraging and Neolithic populations than at resolving distinctions among individual chrono-cultural groups, consistent with previous observations (Martin et al., 2025). Importantly, they demonstrate that EDJ morphology consistently provides higher discriminatory power than bony labyrinth morphology on this sample of Middle Nile Valley individuals.

Supplementary Fig. S1: Maximum (right) and minimum (left) conformation for the first two principal component of the principal component analysis computed on the deformation fields of the vestibule and SCCs.

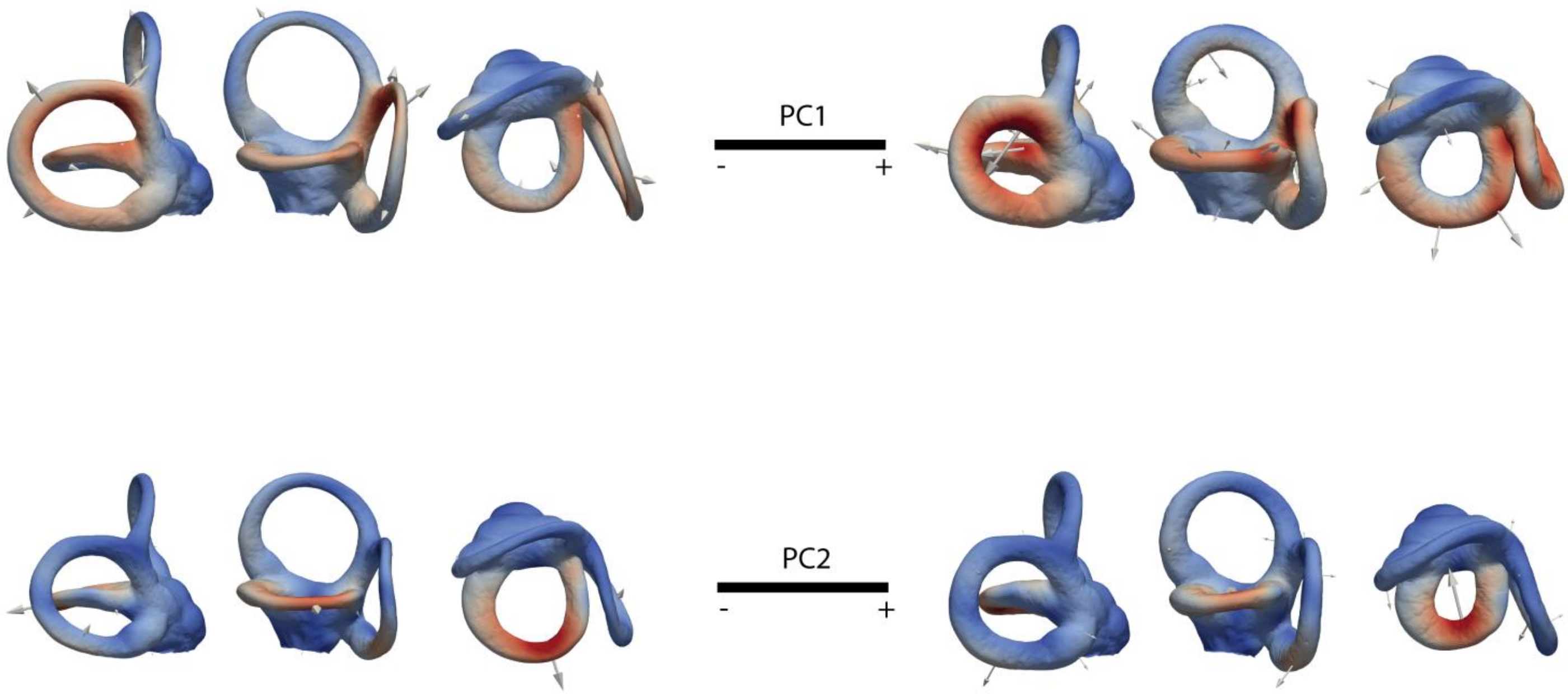

Deformations from the sample global mean shape (GMS) are represented by a colour scale, with red and blue corresponding to maximal (0.6 mm) and no deformation from the average respectively. Arrows indicate the directionality of the deformation.

Supplementary Fig. S2: Principal component analyses (PCA; PC3-4) computed on the deformation fields of the vestibule and SCCs (**A**), or on the whole bony labyrinth and based on 2D measurements (**B**) and on landmark Procrustes coordinates (**C**) of the whole sample considered in this study. Source data are provided as a Source Data file.

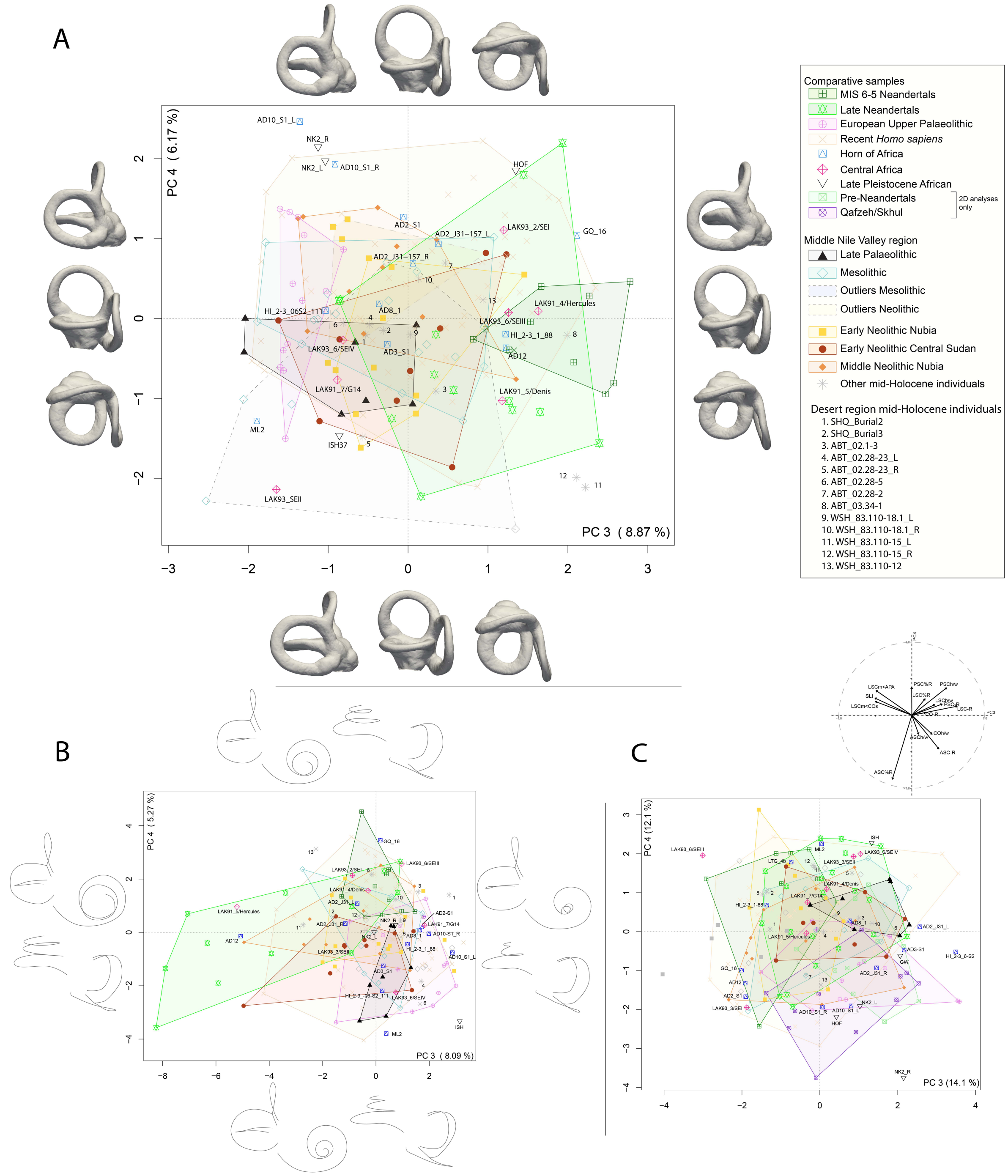

*The extreme conformations (**A-B**) correspond to the maximum and minimum values on the axes, represented by simulated vestibule and SCCs surfaces in posterior, lateral and supero-posterior views (**A**) or by wireframes comprised of the semi-landmarks in posterior and infero-medial views (**B**). The biplot in panel (C) shows the principal component loadings. MIS = Marine Isotope Stage.*

Supplementary Fig. S3: Principal component analyses (PCA) computed on the deformation fields of the vestibule and SCCs (**A**), or on the whole bony labyrinth and based on 2D measurements (**B**) and on landmark Procrustes coordinates (**C**) with the Middle Nile Valley region Mid-Holocene additional individuals labelled.

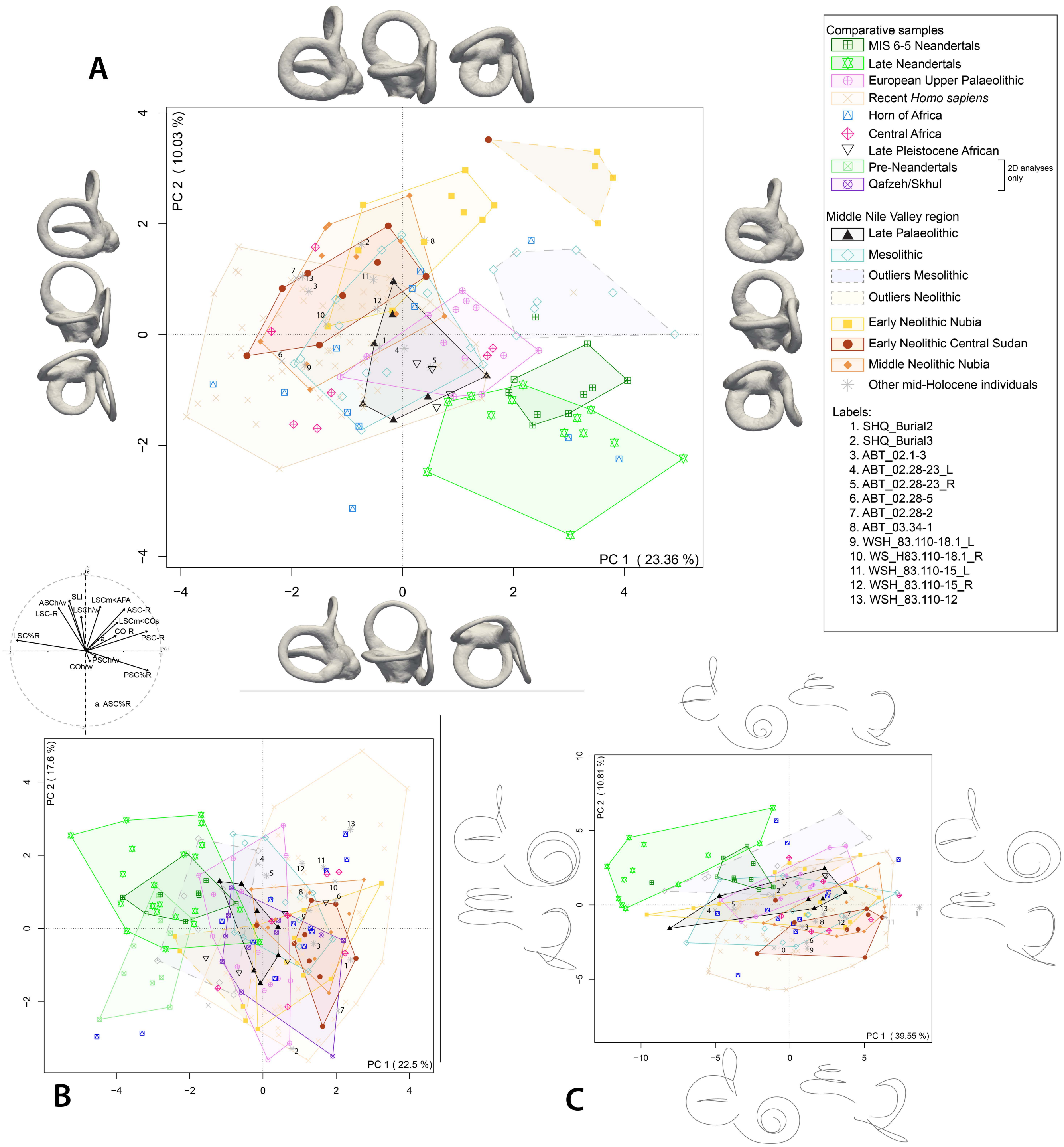

*The extreme conformations (**A-C**) correspond to the maximum and minimum values on the axes, represented by simulated vestibule and SCCs surfaces in posterior, lateral and supero-posterior views (**A**) or by wireframes comprised of the semi-landmarks in posterior and infero-medial views (**C**). The biplot in panel (**B**) shows the principal component loadings. MIS = Marine Isotope Stage.*

Supplementary Fig. S4: Between-group principal component analyses (bgPCA) computed on the deformation fields of the vestibule and SCCs (**A**), or on the whole bony labyrinth and based on landmark Procrustes coordinates (**B**) and on 2D measurements (**C**) of the Middle Nile Valley sample and *a posteriori* projection of the Abu Tabari, Wadi Shaw and Shaqadud individuals.

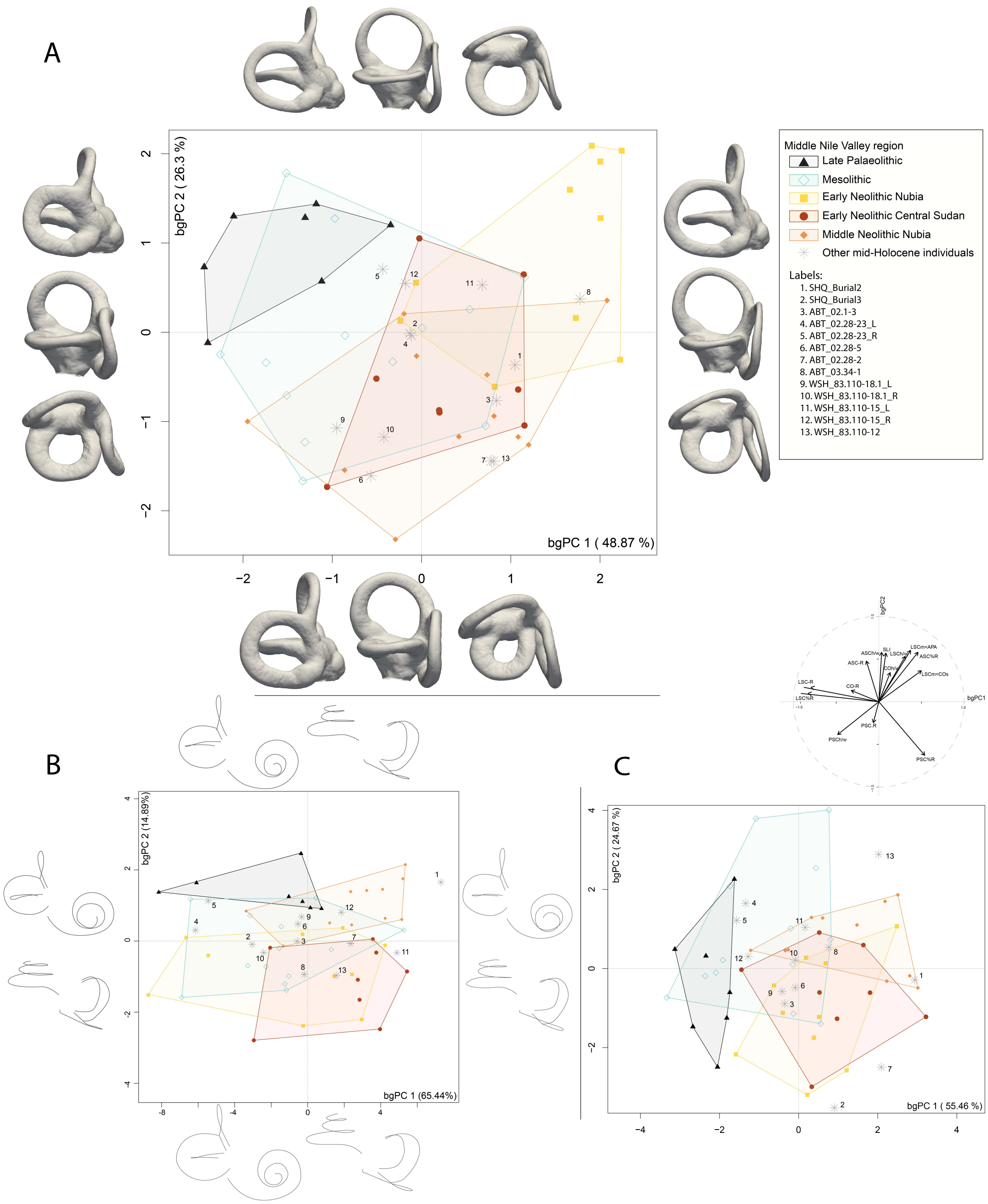

*The extreme conformations (**A-B**) correspond to the maximum and minimum values on the axes, represented by simulated vestibule and SCCs surfaces in posterior, lateral and supero-posterior views (**A**) or by wireframes comprised of the semi-landmarks in posterior and infero-medial views (**B**). The biplot in panel (**C**) shows the principal component loadings.*

Supplementary Fig. S5: Principal component analyses (PCA) computed on the deformation fields of the vestibule and SCCs (**A**), or on the whole bony labyrinth and based on 2D measurements (**B**) and on landmark Procrustes coordinates (**C**) with the Shum Laka individuals labelled.

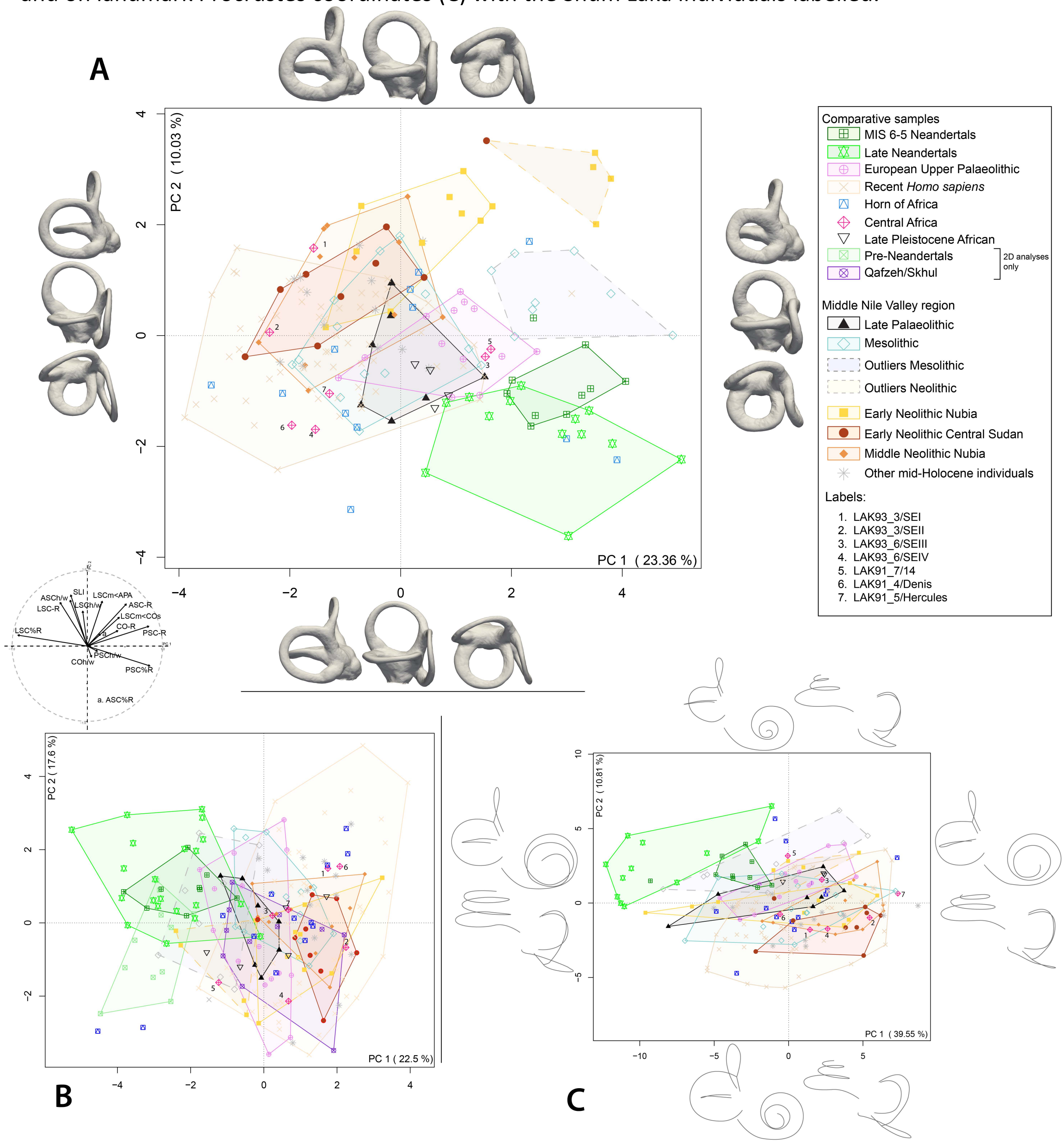

*The extreme conformations (**A-C**) correspond to the maximum and minimum values on the axes, represented by simulated vestibule and SCCs surfaces in posterior, lateral and supero-posterior views (**A**) or by wireframes comprised of the semi-landmarks in posterior and infero-medial views (**C**). The biplot in panel (**B**) shows the principal component loadings. MIS = Marine Isotope Stage.*

Supplementary Fig. S6: Principal component analyses (PCA) computed on the deformation fields of the vestibule and SCCs (**A**), or on the whole bony labyrinth and based on 2D measurements (**B**) and on landmark Procrustes coordinates (**C**) with the Horn of Africa individuals labelled.

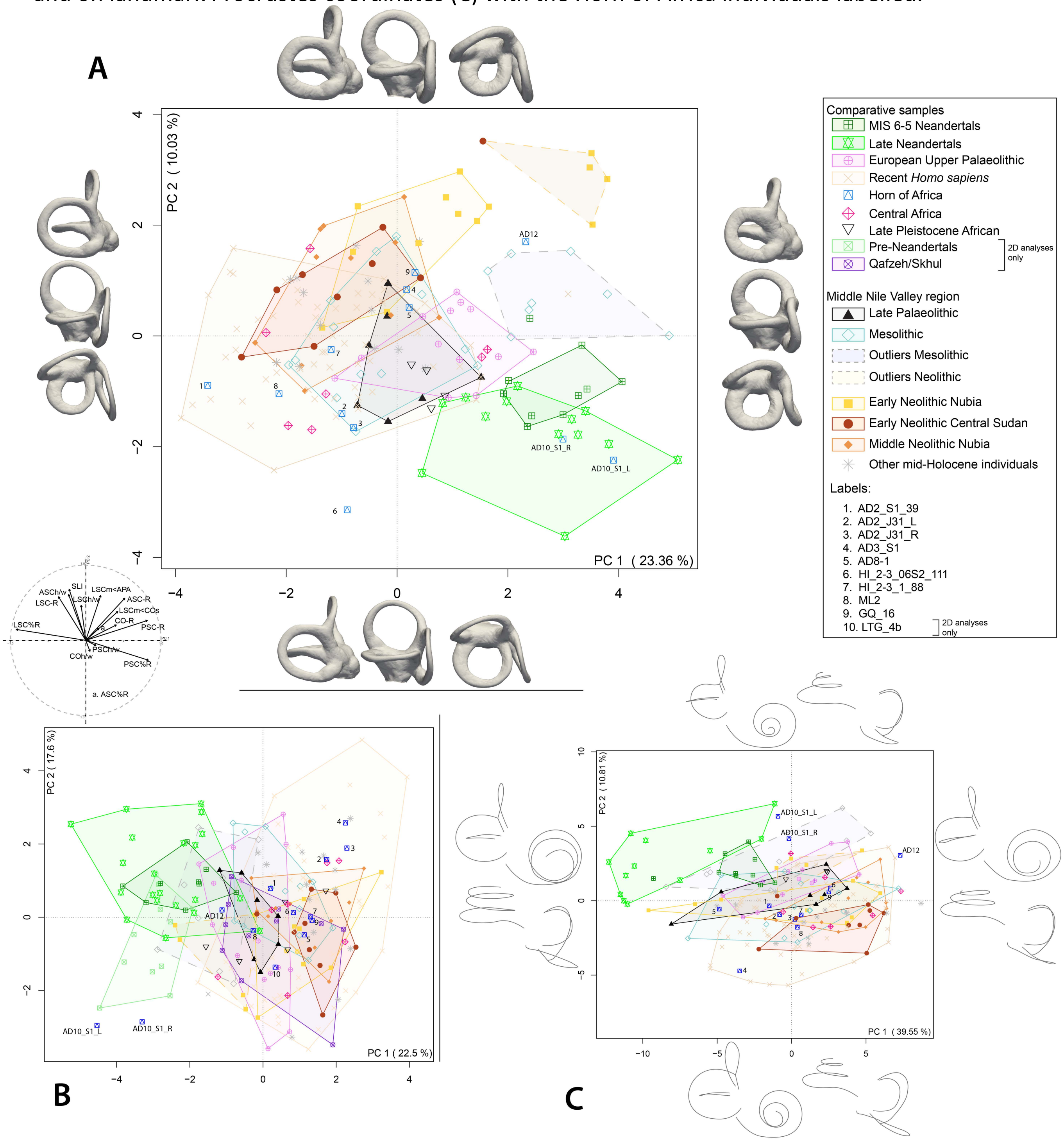

*The extreme conformations (**A-C**) correspond to the maximum and minimum values on the axes, represented by simulated vestibule and SCCs surfaces in posterior, lateral and supero-posterior views (**A**) or by wireframes comprised of the semi-landmarks in posterior and infero-medial views (**C**). The biplot in panel (**B**) shows the principal component loadings. MIS = Marine Isotope Stage.*

Supplementary Fig. S7: Principal component analyses (PCA) computed on the deformation fields of the vestibule and SCCs (**A**), or on the whole bony labyrinth and based on 2D measurements (**B**) and on landmark Procrustes coordinates (**C**). Analyses restricted to the *Homo sapiens* sample.

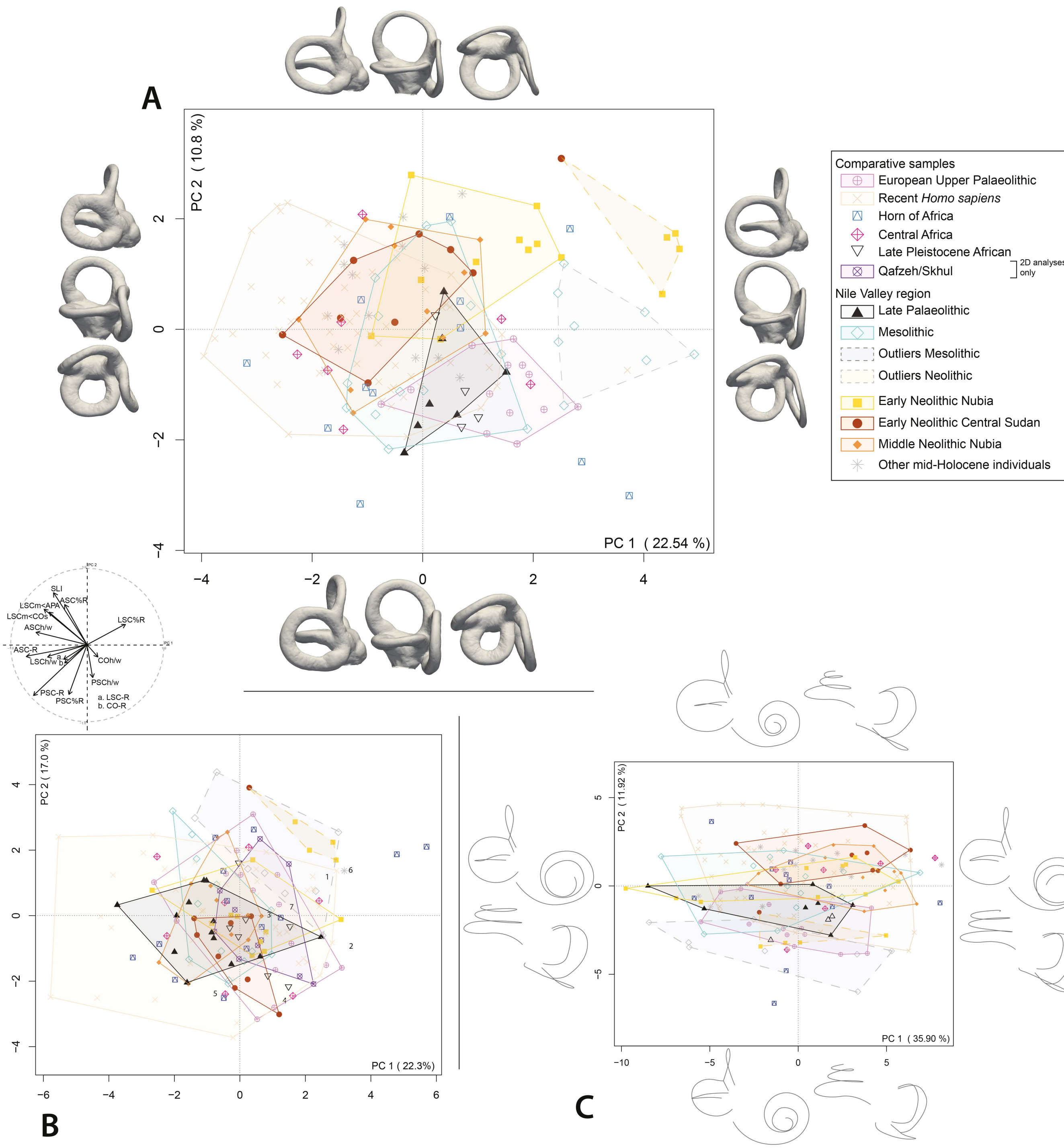

*The extreme conformations (**A-C**) correspond to the maximum and minimum values on the axes, represented by simulated vestibule and SCCs surfaces in posterior, lateral and supero-posterior views (**A**) or by wireframes comprised of the semi-landmarks in posterior and infero-medial views (**C**). The biplot in panel (**B**) shows the principal component loadings. MIS = Marine Isotope Stage.*

Supplementary Fig. S8: Principal component analyses (PCA) computed on the deformation fields of the vestibule and SCCs of the archaeological specimens only (**A**), and subsequent Mahalanobis distances between all archaeological *Homo sapiens* individuals and the centroid, compared to the individuals with inner ear-specific pathological changes.

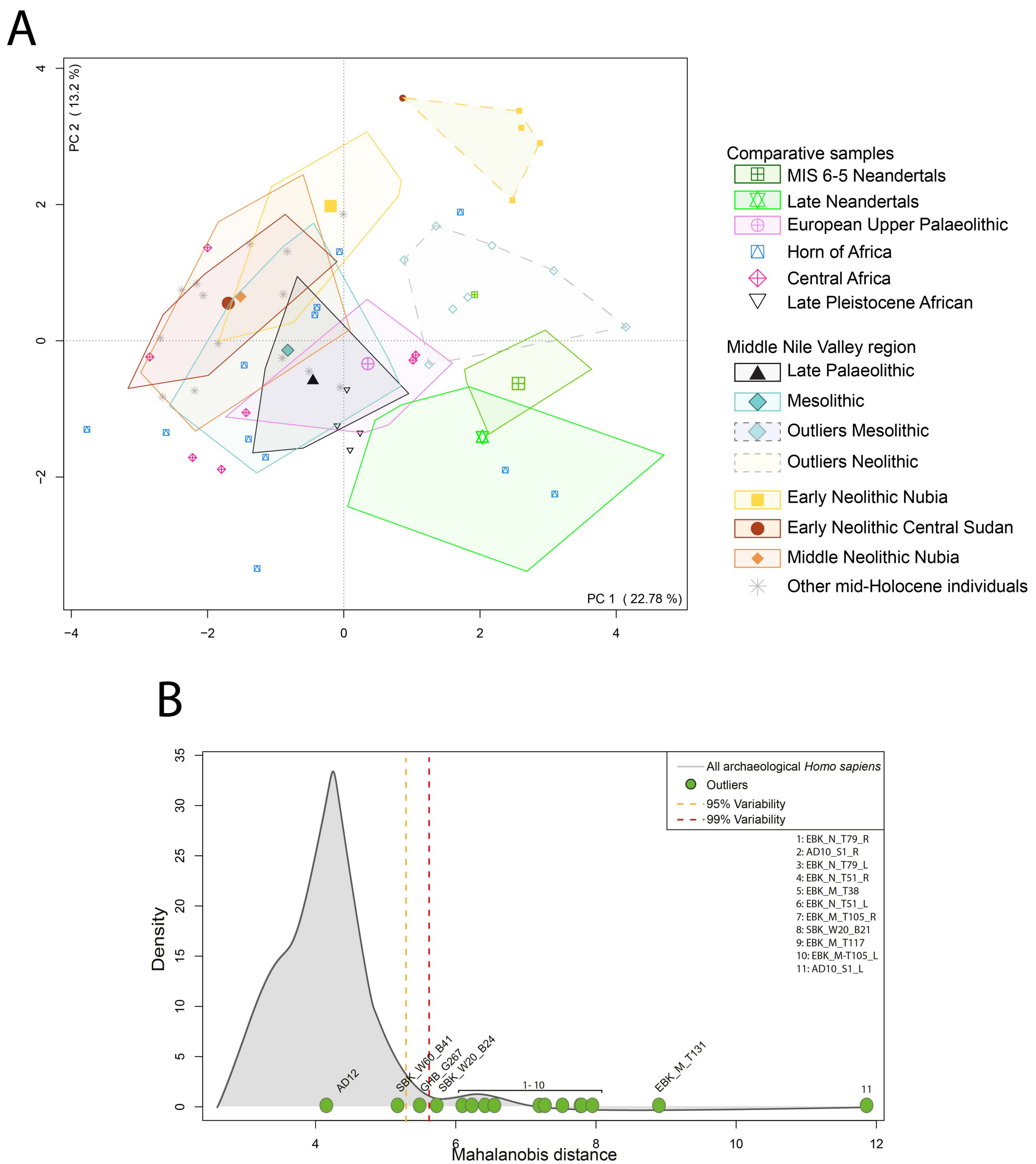

Supplementary Fig. S9: Views of the outlier bony labyrinths. Individuals from the Middle Nile Valley Early Holocene assemblage at El-Barga. *From left to right: posterior, lateral, supero-posterior, anterior and supero-medial views.*

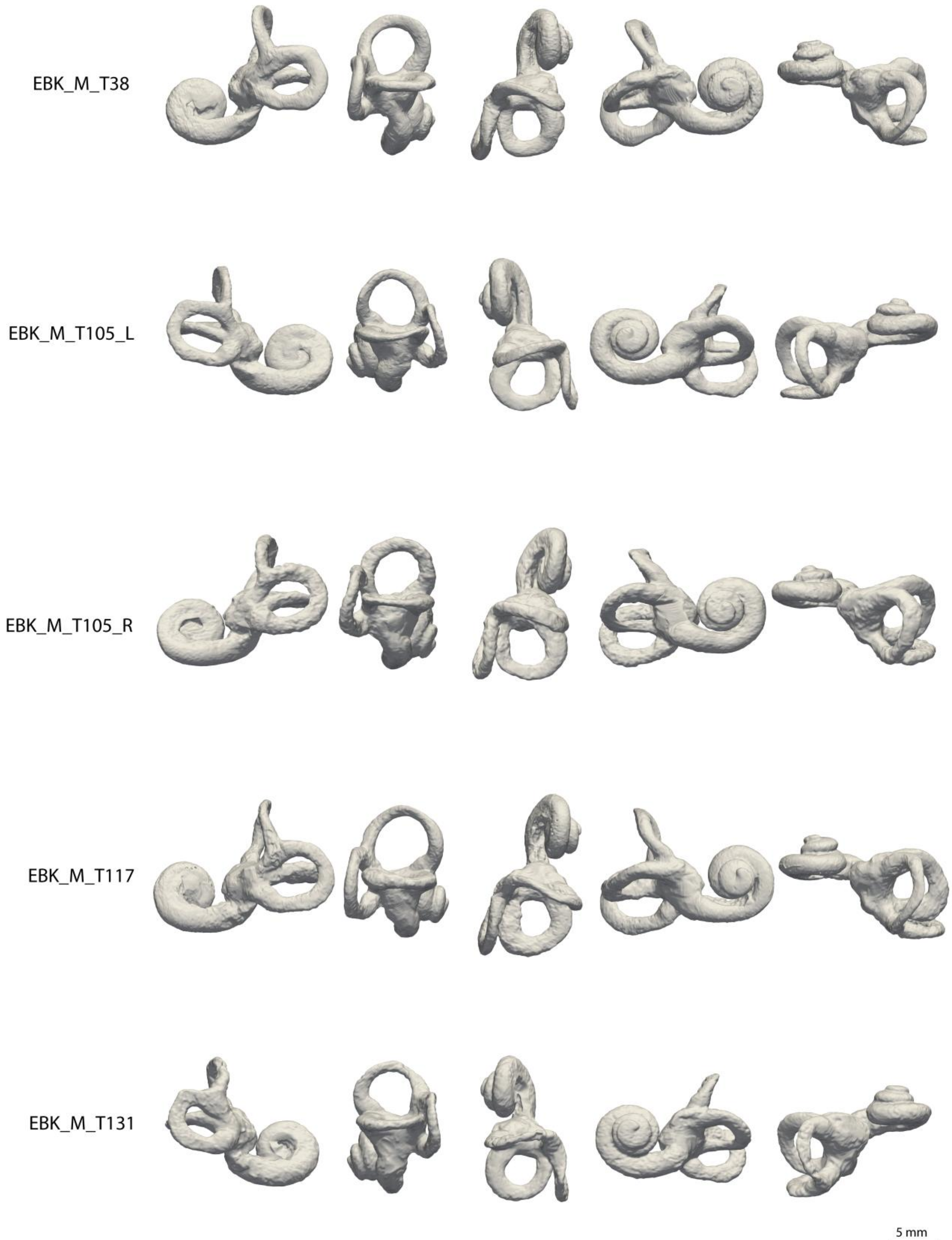

Supplementary Fig. S10: Views of the outlier bony labyrinths. Individuals from the Middle Nile Valley Early Holocene assemblages from Fox Hill (SBK_W20) and Sphinx (SBK_W60). *From left to right: posterior, lateral, supero-posterior, anterior and supero-medial views.*

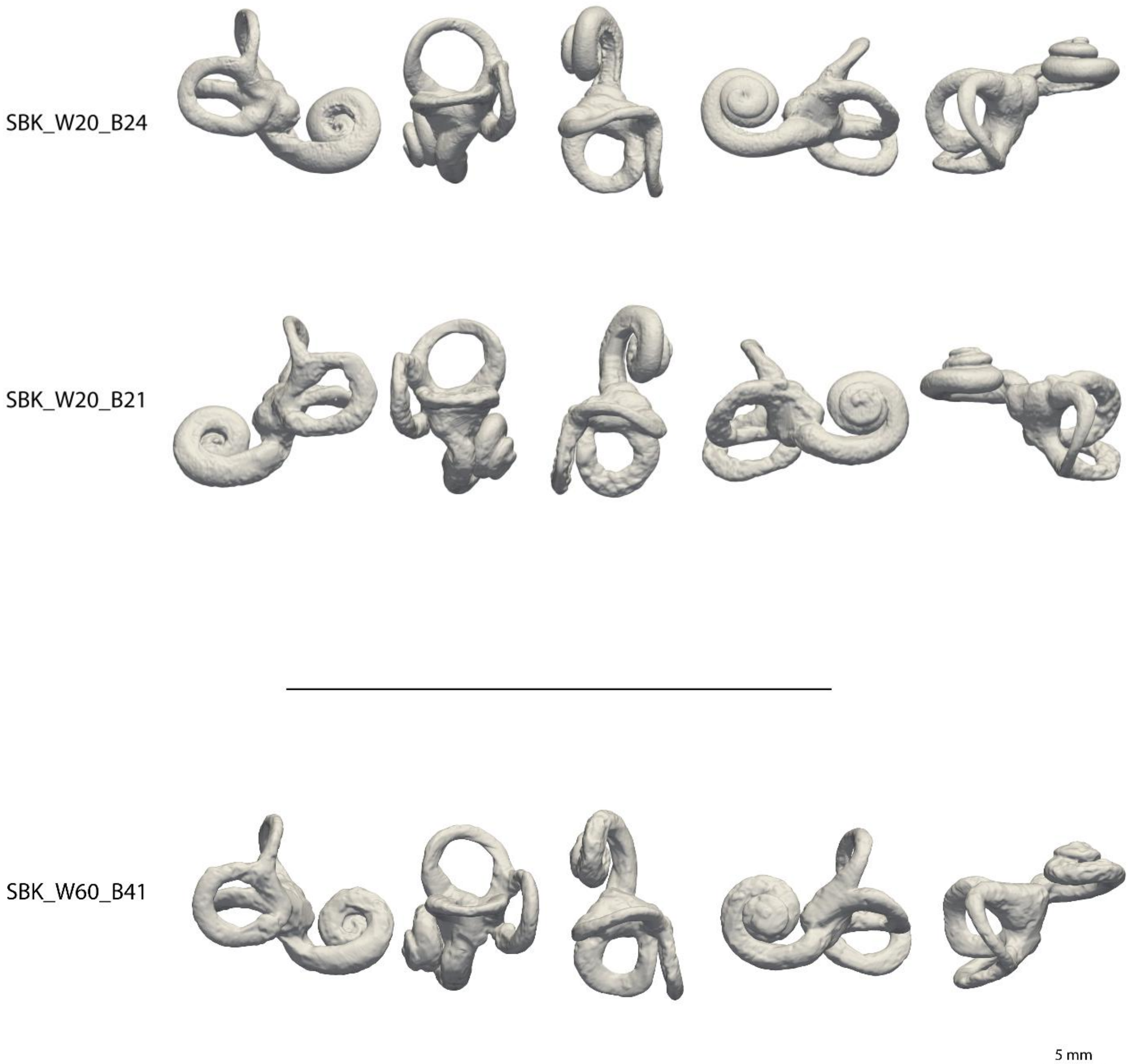

Supplementary Fig. S11: Views of the outlier bony labyrinths. Individuals from the Middle Nile Valley Mid-Holocene assemblages from El-Barga and Ghaba. *From left to right: posterior, lateral, supero-posterior, anterior and supero-medial views.*

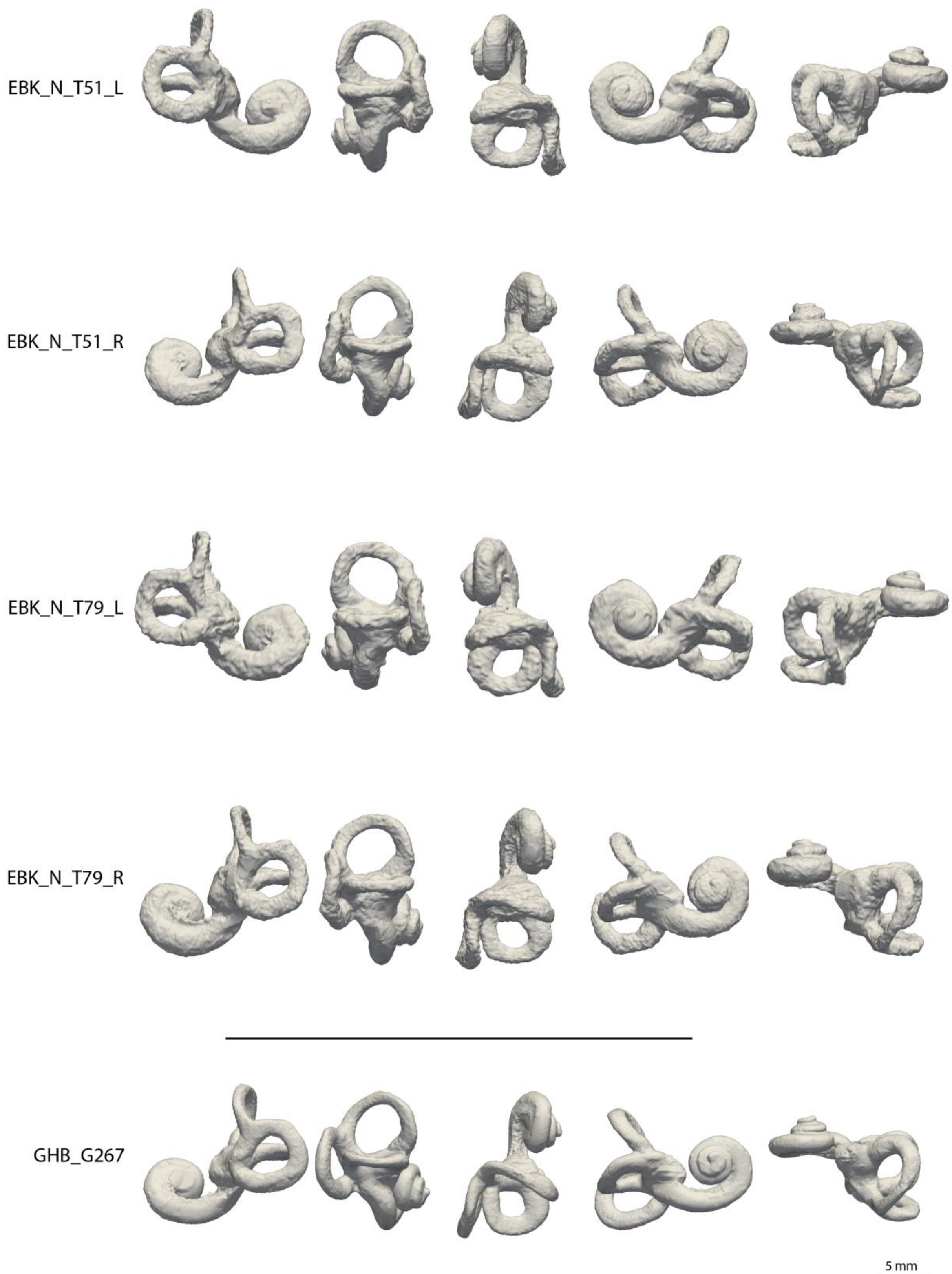

Supplementary Fig. S12: Views of the outlier bony labyrinths. Individuals from the Horn of Africa mid-Holocene assemblages from Ali Daba, and recent individual from South Africa (unilateral deformation). *From left to right: posterior, lateral, supero-posterior, anterior and supero-medial views.*

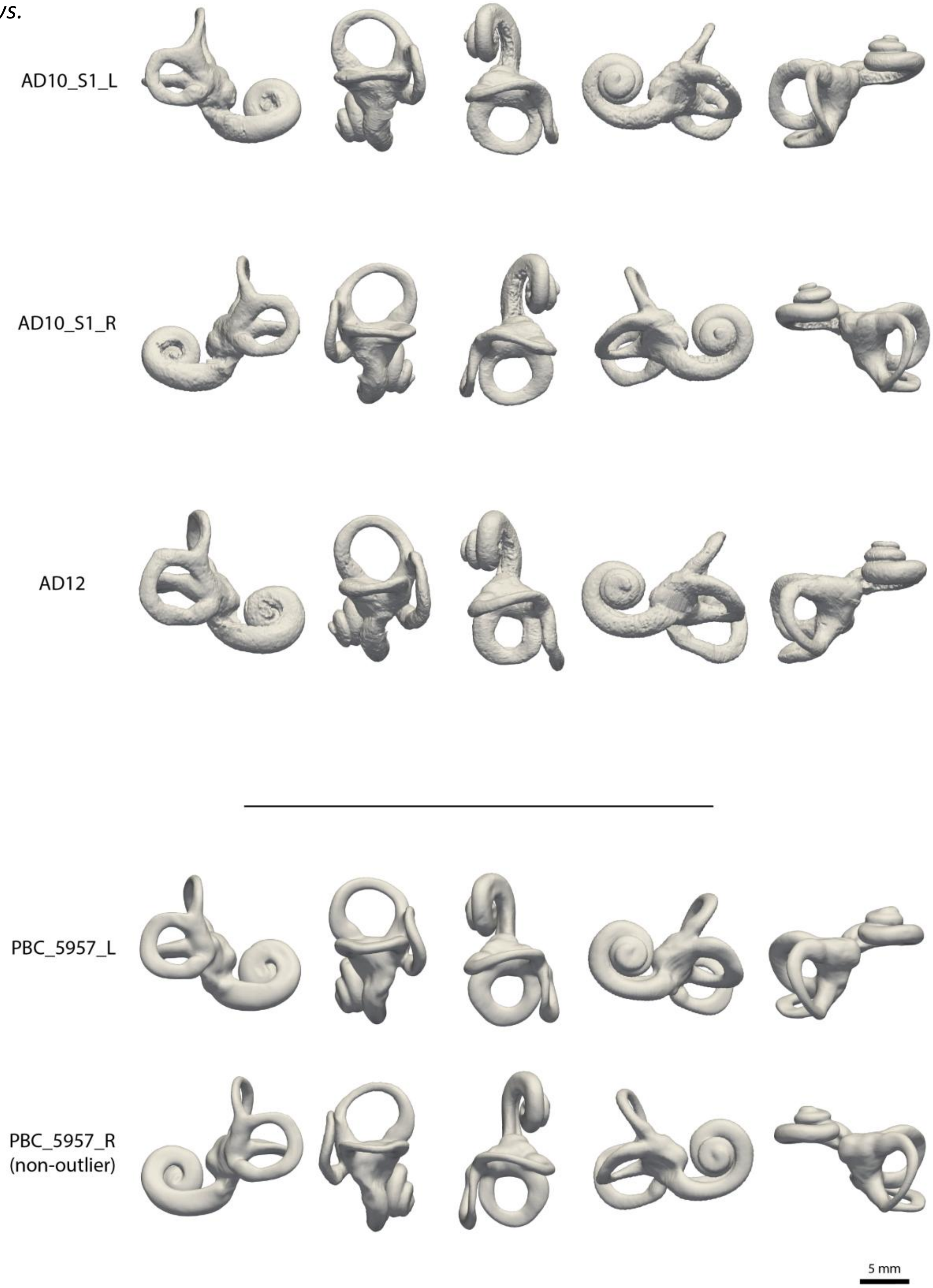

Supplementary Fig. S13: Comparison of the analyses on the EDJ of UM1s (**A**), UM2s (**B**) and on the bony labyrinth (**C**) of the Middle Nile Valley sample. Only the individuals present in both dental and bony labyrinth analyses are labelled. **A** and **B** are derived from Martin et al. (2025).

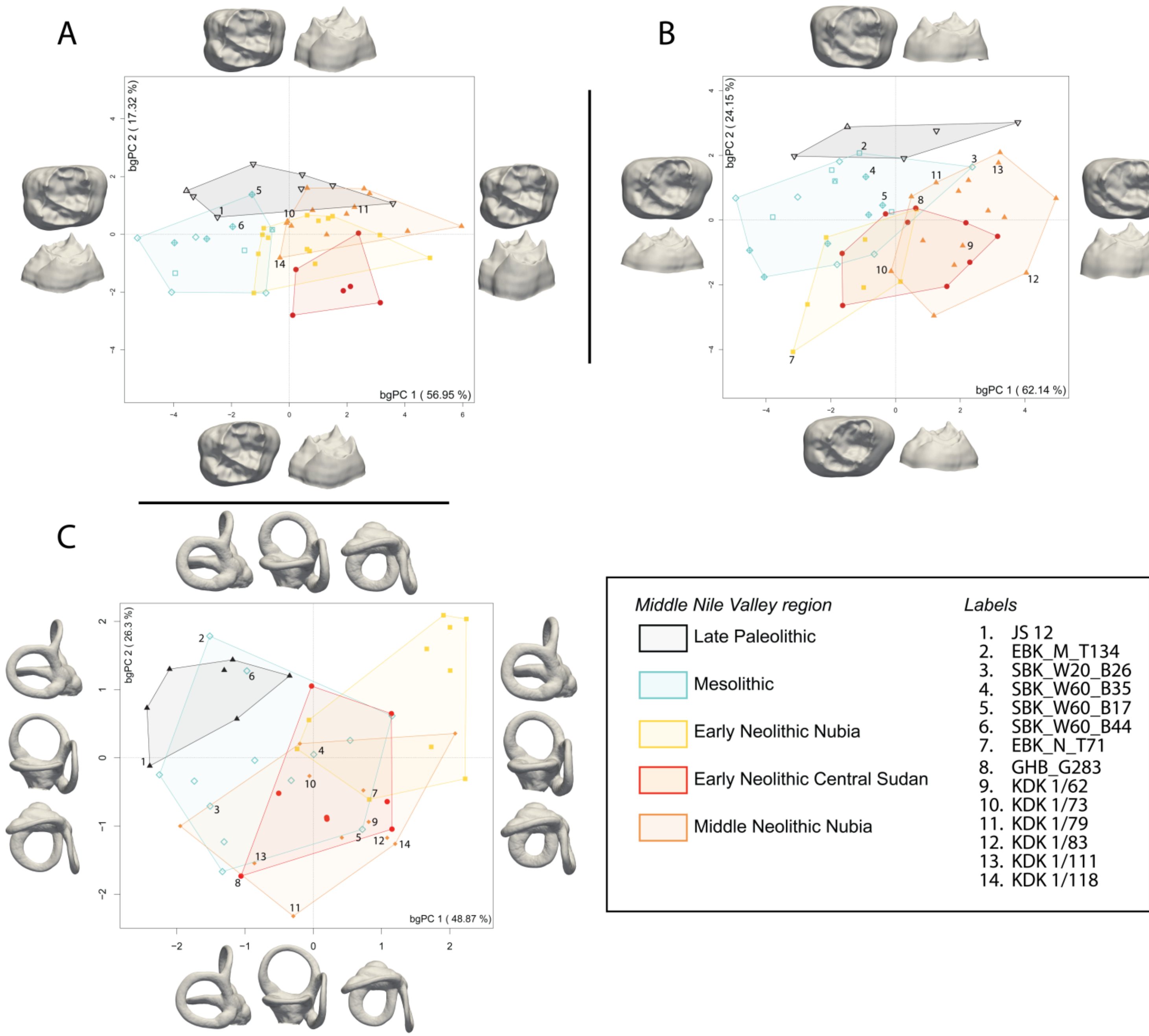

Supplementary Fig. S14: Mahalanobis distances between all non-pathological *Homo sapiens* individuals and the centroid, compared to the individuals with inner ear-specific pathological changes.

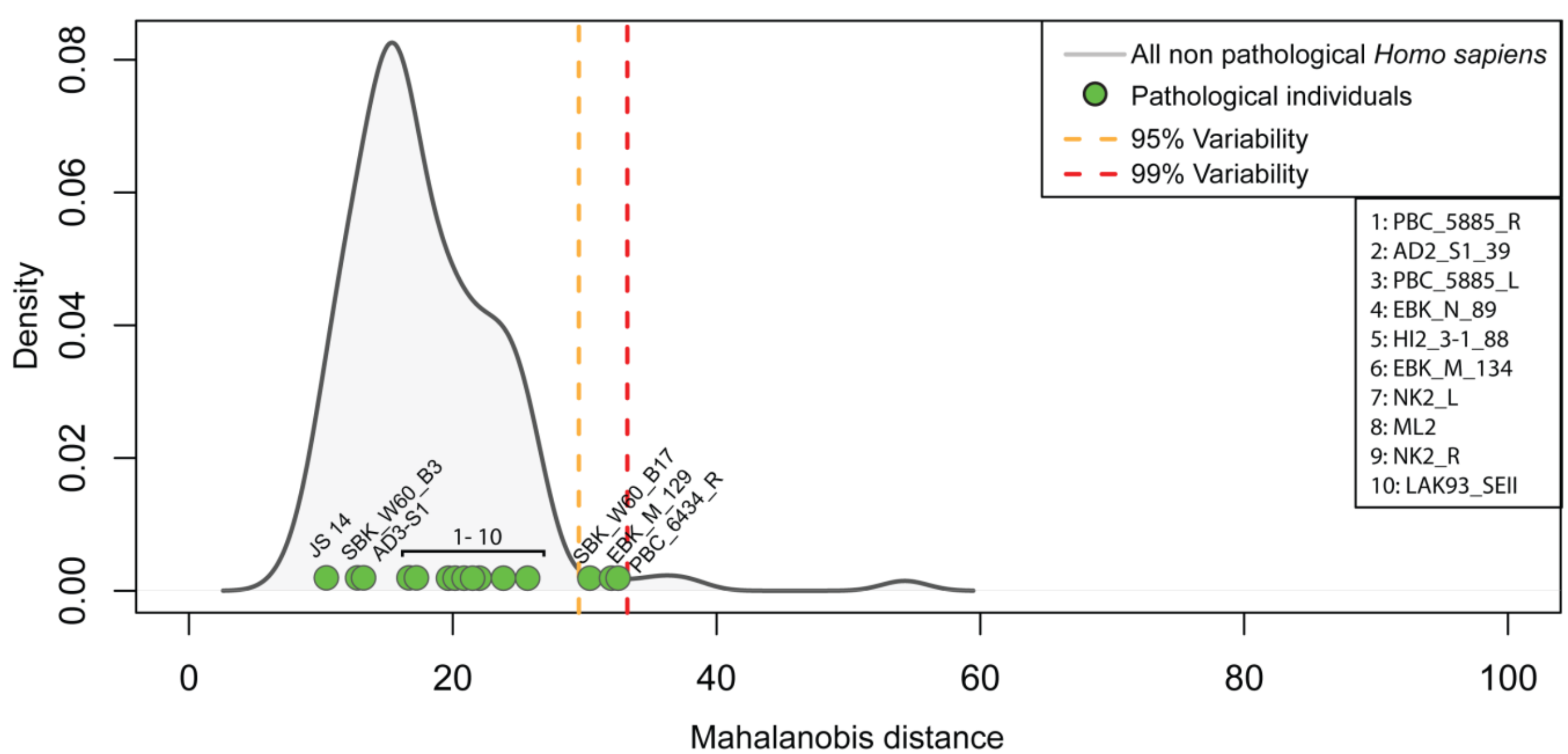

Supplementary Fig. S15: Segmentation error test. Principal component analysis computed on the deformation fields of the vestibule and SCC of four individuals, segmented three times by N.M.

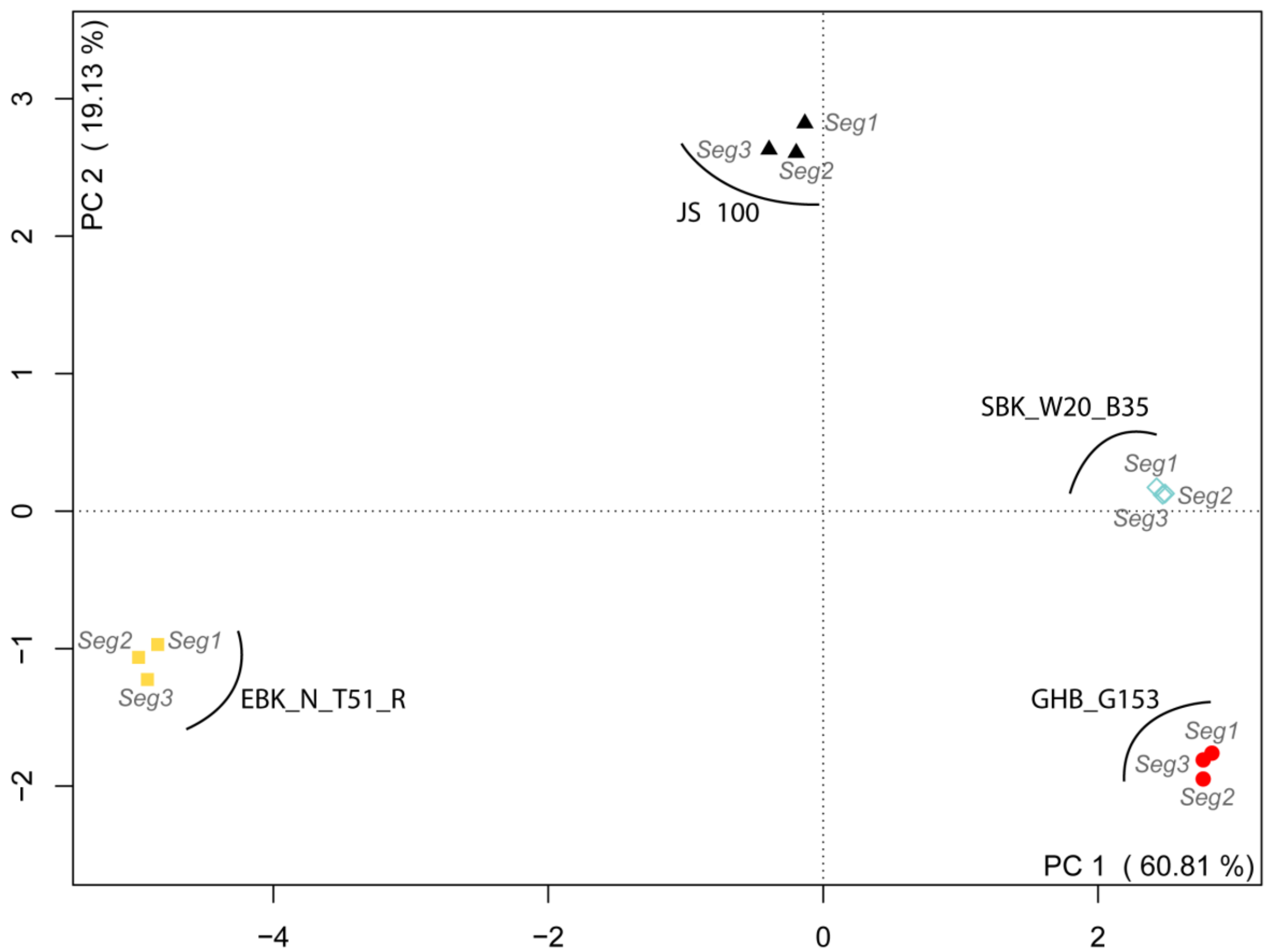

Four individuals were segmented three times by N.M., chosen to represent various preservation and infilling conditions. Principal component analysis of the resulting shapes shows that replicates cluster tightly. Quantitative assessment of replicate variability was performed by comparing the average Euclidean distance between replicates to the average inter-individual distance on PC1-3 (92.2 % of variance). These within-individual distances were consistently low (systematically < 5 % of the average inter-individual distance), indicating that measurement error is small relative to biological variation.

Supplementary Fig. S16: Reorientation steps in Avizo software used for the 2D measurement analysis. (**A**) Orientation of the transverse plane following the axis of the lateral semi-circular canal, comparable to the method described in Gunz et al. (2012). (**B**) Definition of the sagittal plane as perpendicular to the transverse plane, which is then (**C**) manually adjusted to match the orientation described by Spoor and Zonneveld (1995; Fig. 5j).

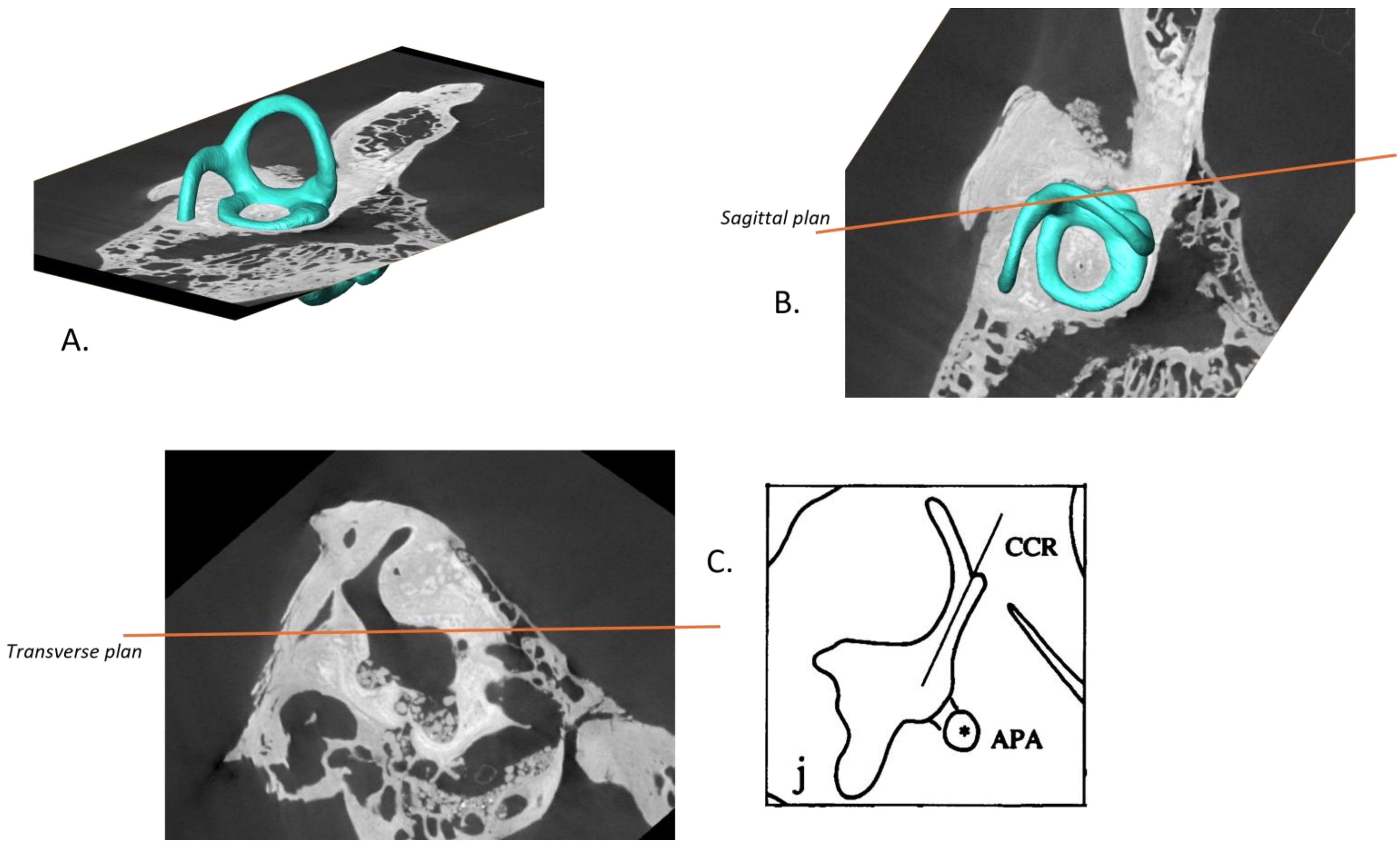

Supplementary Fig. S17: Landmarks (red) and semi-landmarks (yellow) used in the landmark-based analyses. See **Table S6** for further details on the order and number of landmarks and semi-landmarks. *Views of a left bony labyrinth in supero-lateral (top-left), posterior (top-right), and infero-lateral (bottom) perspectives.*

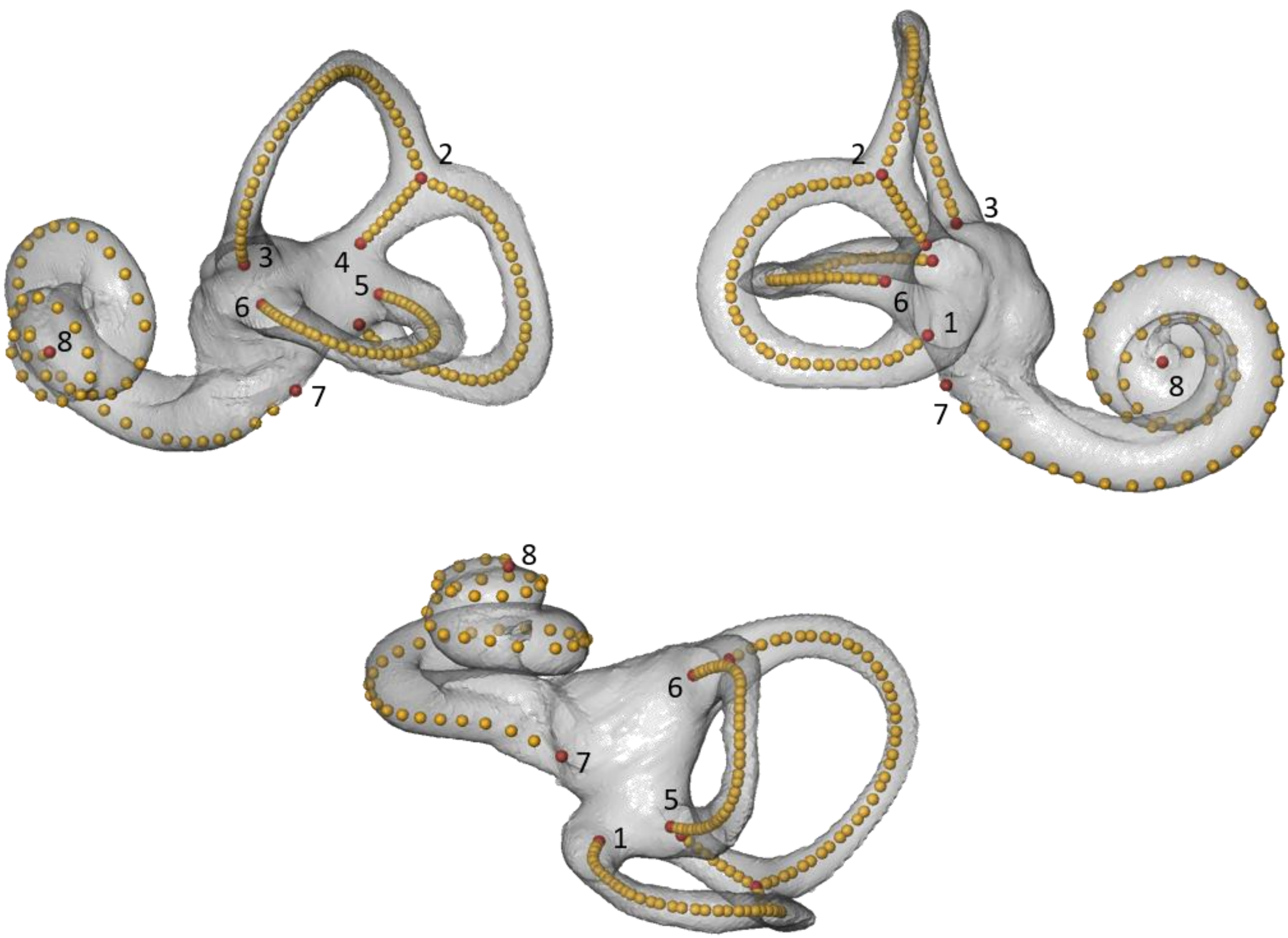

Supplementary Fig. S18: Principal component analysis (PCA) based on the original Procrustes landmark coordinates dataset (i.e., **Fig. 3C**) with the replicate individuals *a posteriori* projected, together with a comparison of Euclidean inter-individual and inter-replicate distances. Boxplots represent the distribution of Euclidean distances between all pairs of individuals, computed using the first 18 PCs (>1 % of total variance, 88.9 % in total), while individual points represent Euclidean distances between a replicate and the original measure.

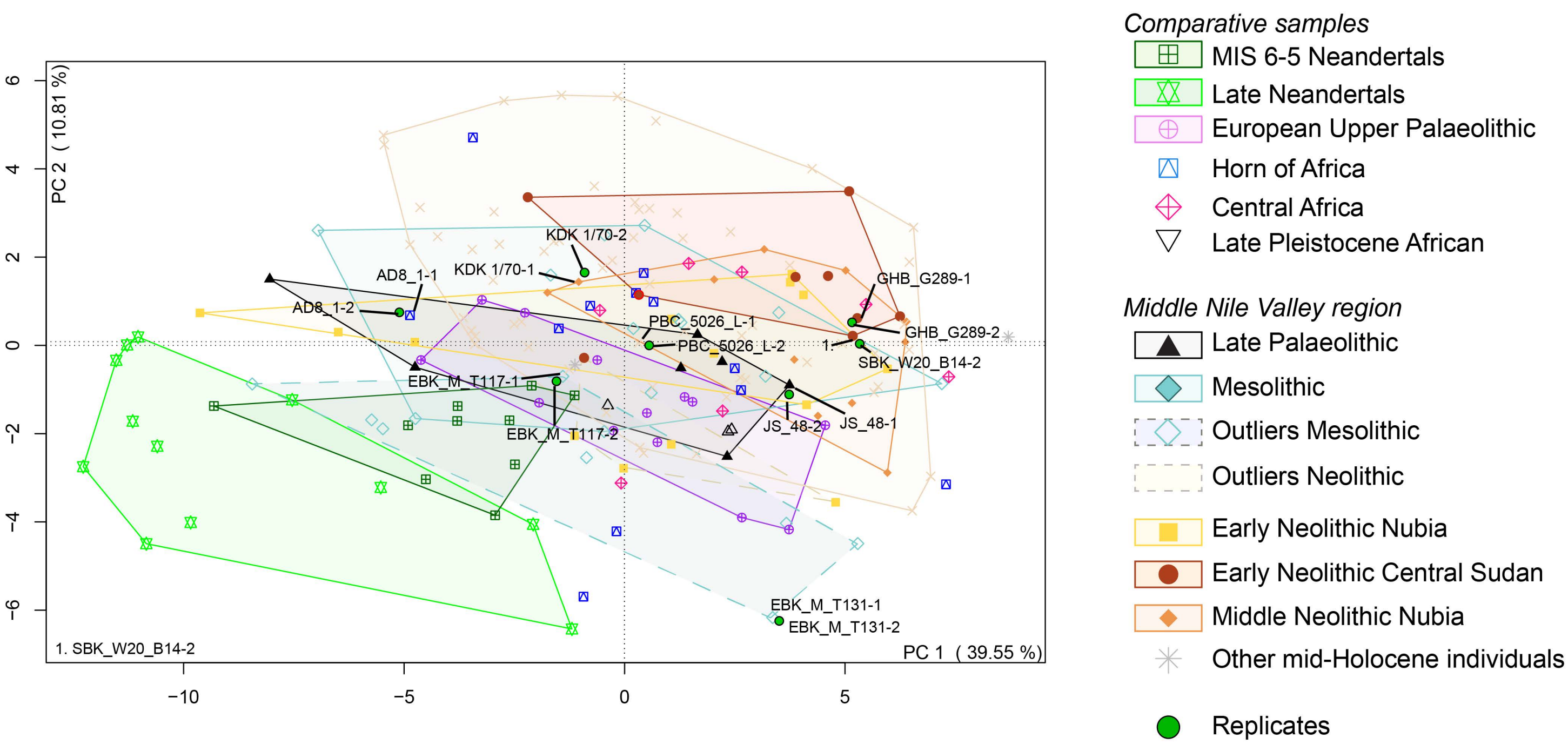

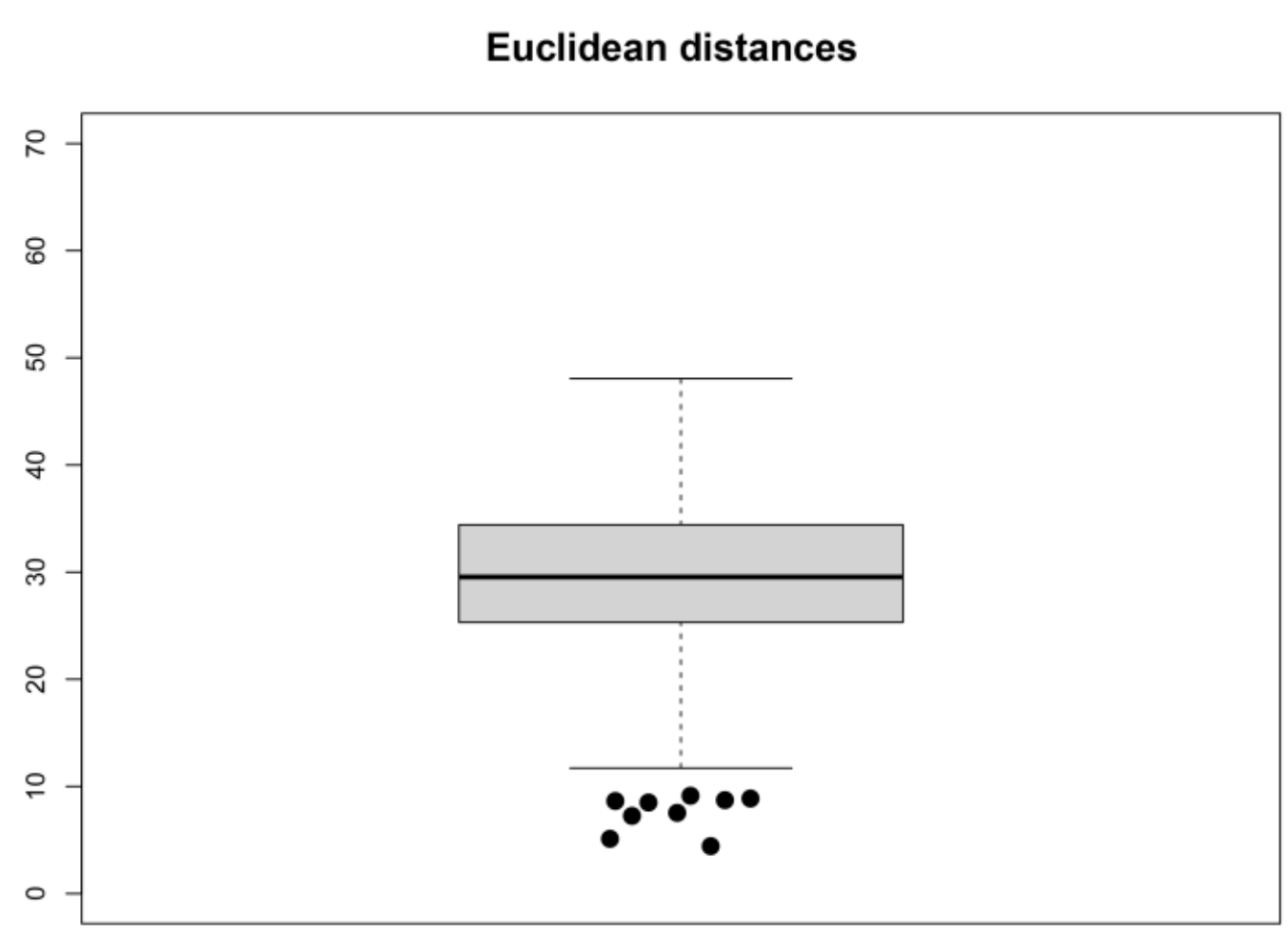

Replicate measurements cluster closely in the PCA space, and inter-replicate distances are consistently much smaller than inter-individual distances. This pattern indicates high repeatability of the landmarking protocol and a negligible effect of intra-observer error.

Supplementary Fig. S19: Principal component analysis based on the deformation fields of the vestibule and SCC newly computed on the sample restricted to the archaeological material.

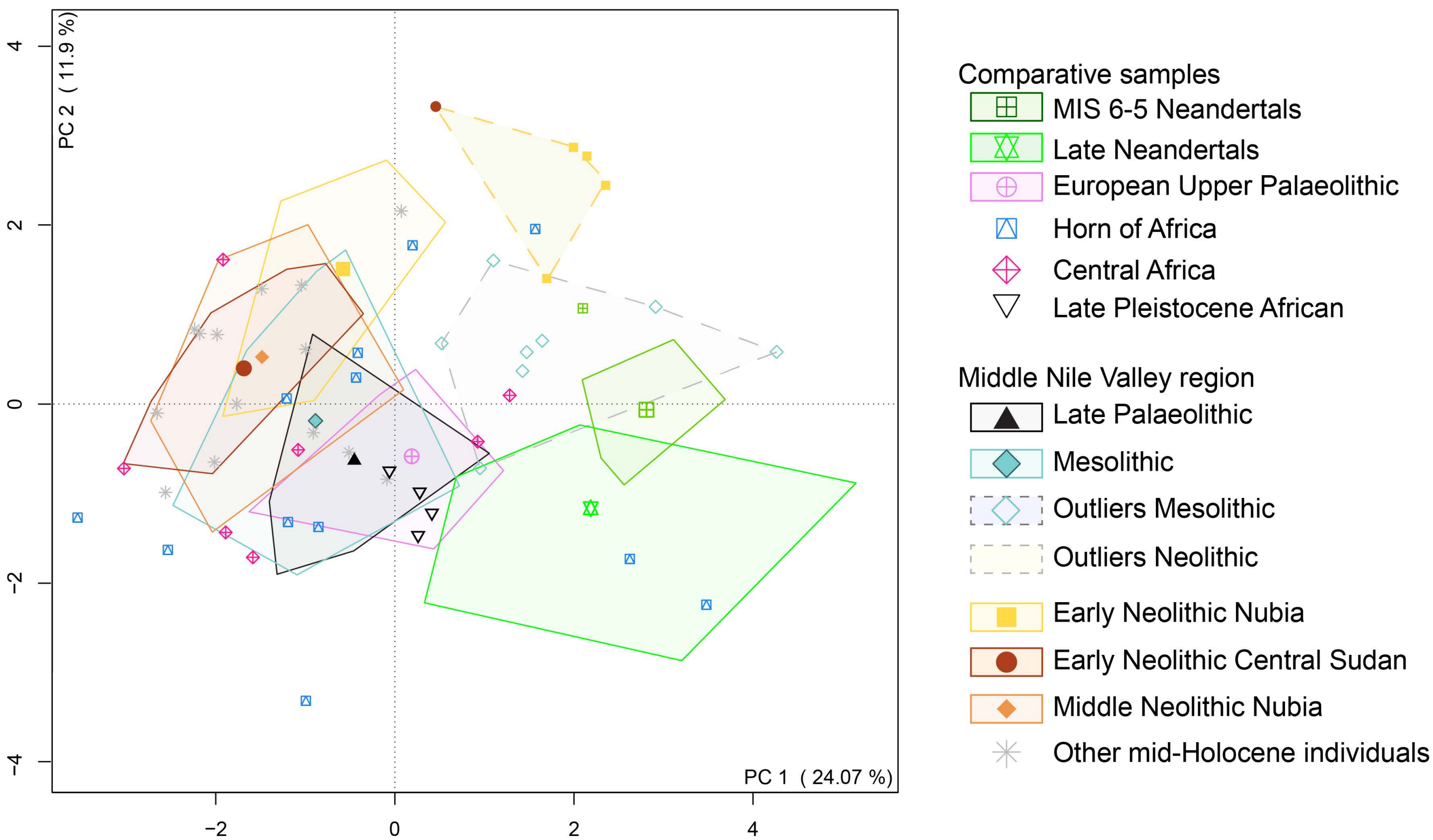

Supplementary Fig. S20: Cross-validated between-group principal component analyses (cv-bgPCA) computed on the deformation fields of the vestibule and SCCs (**A**), or on the whole bony labyrinth and based on landmark Procrustes coordinates (**B**) and on 2D measurements (**C**) of the Middle Nile Valley sample.

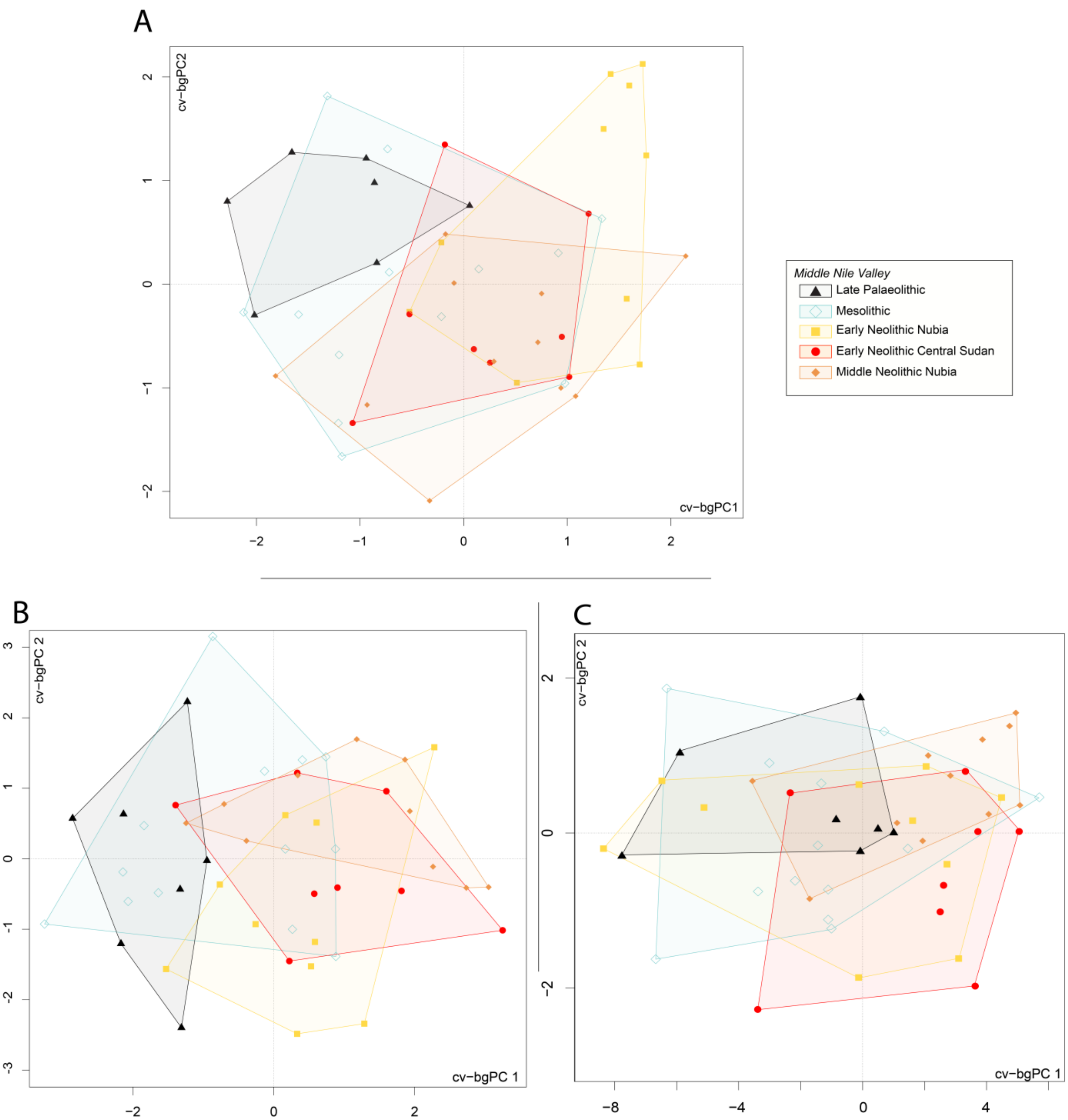

Supplementary Fig. S21: Principal component analysis computed on the deformation fields of the vestibule and SCCs. Only pairs of bony labyrinths sampled from the same individual are shown, connected by dark blue arrows. The arrows point from the left antimere towards the right one.

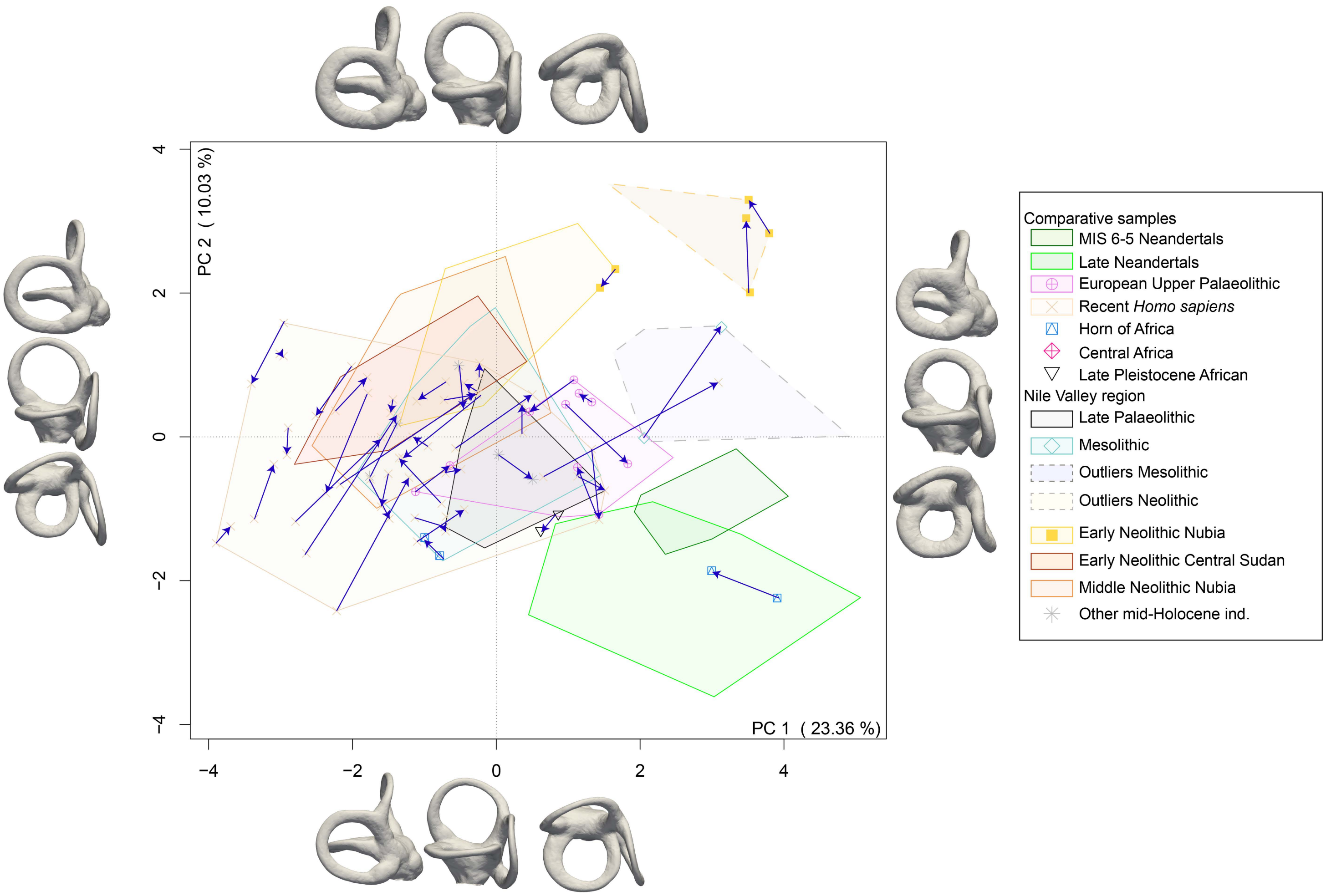

Supplementary Fig. S22: Differences in preservation states in the sample. (A–C) preservation impacting EVA assessment; (D–E) preservation relevant to identifying the VA–PSCC fistula. *(VA: vestibular aqueduct; PSCC: posterior semi-circular canal; ASCC: anterior semi-circular canal; LSCC: lateral semi-circular canal; CC: common crus). (A-B) measurements not taken due to the inability to determine the VA contours reliably. (E) comparison of transverse and sagittal views confirmed the presence of a fistula.*

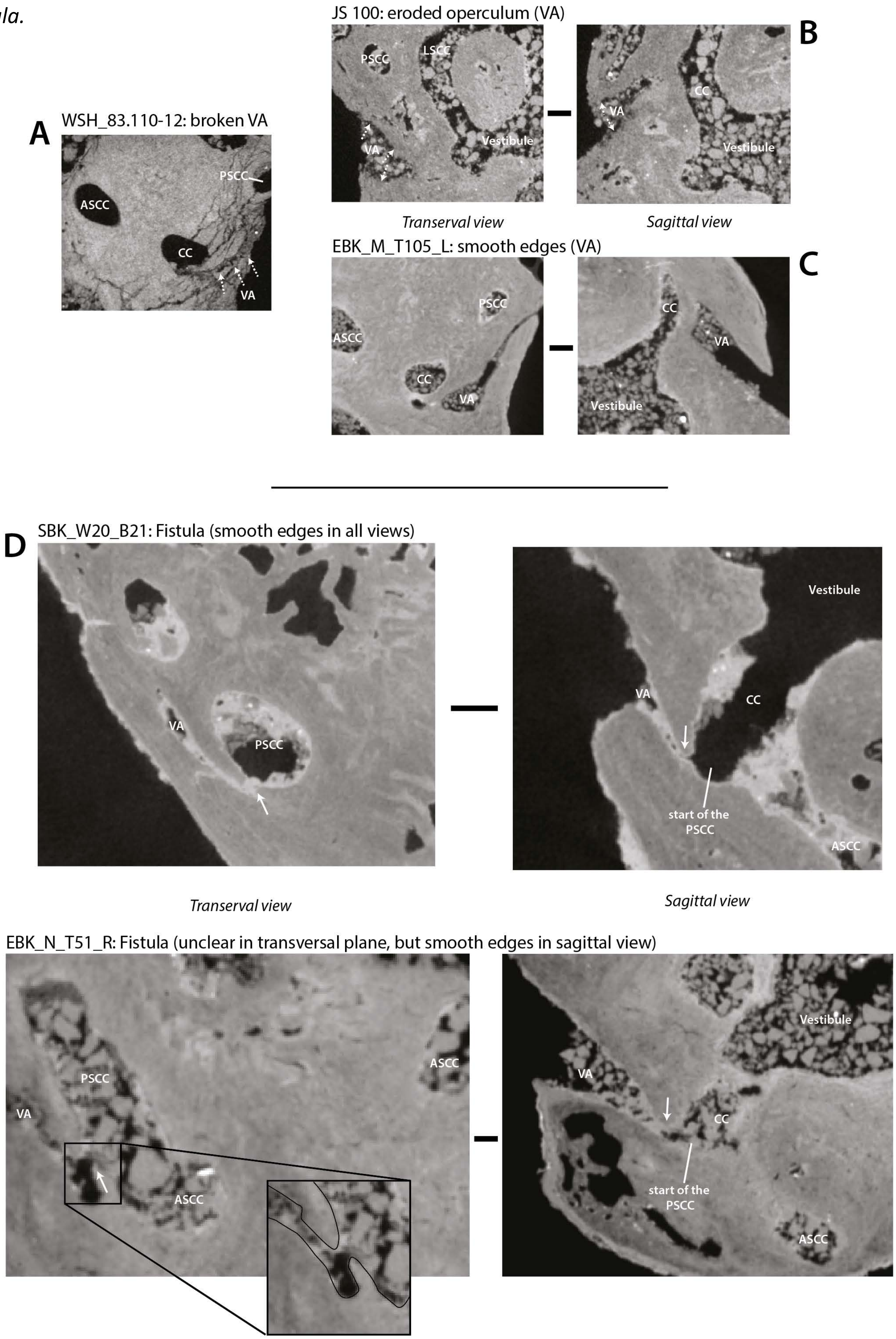

Supplementary Table S1: Results of the linear ordinary least squares regressions of PC scores against the natural logarithm of the centroid size (i.e., of the SCC and vestibule for the DSM analysis, and of the whole landmark set for the LM analyses). Two-sided t-tests were used, with no correction for multiple comparisons applied. Source data are provided as a Source Data file.

Whole sample configuration (i.e., with Neandertals):

	Surface registration				*Landmarks*			
	DF	F-statistic	R^2	p-value	DF	F-statistic	R^2	p-value
PC1	193	31.81	0.142	**<0.0001**	191	10.42	0.052	**0.002**
PC2		0.11	0.001	0.739		1.43	0.007	0.233
PC3		1.32	0.007	0.252		0.98	0.005	0.324
PC4		0.20	0.001	0.6574		0.15	0.001	0.695

P-values in bold are statistically significant (p < 0.05).

Sample restricted to *Homo sapiens*:

	Surface registration				*Landmarks*			
	DF	F-statistic	R^2	p-value	DF	F-statistic	R^2	p-value
PC1	170	17.69	0.121	**<0.0001**	168	4.26	0.025	**0.041**
PC2		0.04	<0.001	0.846		13.54	0.075	**0.0003**
PC3		0.13	<0.001	0.716		0.14	<0.001	0.711
PC4		0.01	<0.001	0.913		2.68	0.016	0.104

P-values in bold are statistically significant (p < 0.05).

Sample without the recent South African sample

	Surface registration				*Landmarks*			
	DF	F-statistic	R^2	p-value	DF	F-statistic	R^2	p-value
PC1	134	26.19	0.194	**<0.0001**	132	8.748	0.063	**0.004**
PC2		0.98	0.007	0.325		0.57	0.005	0.453
PC3		1.87	0.014	0.174		0.40	0.003	0.527
PC4		0.38	0.003	0.542		0.49	0.004	0.485

P-values in bold are statistically significant (p < 0.05).

Supplementary Table S2: Results on the between-group pairwise PERMANOVAs (1,000 permutations, based on Euclidean distances) computed on all PC scores for the Middle Nile Valley sample.

Groups		2D measurements			Landmark analysis			Surface registration analysis		
		Sum of squares	*R^2*	*p-value*	*Sum of squares*	*R^2*	*p-value*	*Sum of squares*	*R^2*	*p-value*
LP	Meso	23.18	0.092	0.092	31.35	0.045	0.489	14.76	0.069	0.105
LP	ENeoN	38.67	0.178	**0.002**	42.91	0.060	0.381	39.26	0.224	**0.001**
LP	ENeoCS	40.37	0.222	**0.001**	94.99	0.167	**0.038**	26.21	0.165	**0.001**
LP	MNeoN	53.86	0.229	**0.002**	110.81	0.175	**0.013**	30.52	0.174	**0.001**
Meso	ENeoN	33.30	0.104	**0.013**	30.23	0.035	0.527	34.65	0.131	**0.001**
Meso	ENeoCS	33.80	0.119	**0.026**	91.71	0.125	**0.027**	17.85	0.073	0.087
Meso	MNeoN	30.91	0.096	**0.034**	124.85	0.153	**0.007**	15.64	0.061	0.090
ENeoN	ENeoCS	21.11	0.096	0.076	64.17	0.089	0.185	23.14	0.123	**0.018**
ENeoN	MNeoN	28.13	0.105	**0.033**	84.86	0.107	0.068	25.47	0.126	**0.003**
ENeoCS	MNeoN	16.30	0.075	0.209	38.31	0.071	0.221	14.89	0.079	0.089

P-values in bold are statistically significant ($p < 0.05$). Tests were two-sided, with no correction applied. LP: Late Palaeolithic; Meso: Mesolithic; ENeoN: Early Neolithic Nubia; ENeoCS: Early Neolithic Central Sudan; MNeoN: Middle Neolithic Nubia.

Supplementary Table S3: Typicality probabilities for the Abu Tabari, Wadi Shaw and Shaqadud individuals computed on the bgPC-scores based on the surface registration fields (left), 2D measurements (centre), and landmark Procrustes coordinates (right). Only the highest probability is shown.

	LP	Meso	ENeoN	ENeoCS	MNeoN
ABT_02.1-3	-/-/-	-/-/0.79	-/-/-	0.84/0.78/-	-/-/-
ABT_02.28-2	-/-/-	-/-/-	-/-/0.78	0.48/0.50/-	-/-/-
ABT_02.28-5	-/-/-	-/-/0.59	-/-/-	-/0.49/-	0.58/-/-
ABT_02.28-23_L	-/-/0.72	0.98/0.87/-	-/-/-	-/-/-	-/-/-
ABT_02.28-23_R	-/-/0.55	0.61/0.98/-	-/-/-	-/-/-	-/-/-
ABT_03.34-1	-/-/-	-/-/0.55	0.85/0.84/-	-/-/-	-/-/-
SHQ_Burial 2	-/-/-	-/-/-	-/-/-	0.61/0.15/-	-/-/0.48
SHQ_Burial 3	-/-/-	0.82/-/-	-/0.27/0.65	-/-/-	-/-/-
WSH_83.110-12	-/-/-	-/0.04/0.18	-/-/-	0.48/0.04/-	-/-/-
WSH_83.110-15_L	-/-/-	-/0.96/-	-/-/-	0.72/-/0.75	-/-/-
WSH_83.110-15_R	-/-/-	-/0.64/-	-/-/-	0.42/-/-	-/-/0.83
WSH_83.110-18.1_L	-/-/-	-/0.89/0.88	-/-/-	0.55/-/-	-/-/-
WSH_83.110-18.1_R	-/-/-	-/-/0.96	-/-/-	-/0.74/-	0.43/-/-

LP: Late Palaeolithic; Meso: Mesolithic; ENeoN: Early Neolithic Nubia; ENeoCS: Early Neolithic Central Sudan; MNeoN: Middle Neolithic Nubia

Supplementary Table S4: Measurements of the vestibular aqueduct (VA) and cochlear nerve canal (CNC) width for the individuals presenting congenital pathologies (in mm; only pathological values are shown).

Individual	Site		$VA_{operculum}$	$VA_{mid\text{-}point}$	CNC
Individuals with an outlier morphology					
EBK_M_T105_L	El-Barga (Mesolithic)		2.18	1.70	1.80
EBK_M_T105_R	El-Barga (Mesolithic)		-	-	1.79
EBK_M_T117	El-Barga (Mesolithic)		2.19	1.24	-
EBK_N_T51_L	El-Barga (Neolithic)		2.00	1.34	1.75
EBK_N_T51_R	El-Barga (Neolithic)		2.07	1.03	1.77
EBK_N_T79_R	El-Barga (Neolithic)		-	1.35	-
SBK_W60_B41	Sphinx		2.18	-	-
GHB_G267	Ghaba		-	1.17	-
AD12	Ali Daba		-	-	1.61
AD10_S1_L	Ali Daba		2.01	1.30	-
AD10_S1_R	Ali Daba		2.17	-	-
Individuals with a “non-outlier” morphology					
NK2_L	Nazlet Khater 2		-	1.35	-
NK2_R	Nazlet Khater 2		-	1.21	-
JS 14	Jebel Sahaba		-	1.23	-
EBK_M_T129	El-Barga (Mesolithic)		-	1.14	-
EBK_M_T134	El-Barga (Mesolithic)		2.55	1.12	-
SBK_W60_B3	Sphinx		2.33	1.14	-
SBK_W60_B17	Sphinx		-	1.07	0.97
EBK_N_T89	El-Barga (Neolithic)		2.04	-	-
LAK93_2/SEII	Shum Laka		-	1.36	-
ML2	Mille-Logya		-	-	1.79
AD2_S1_39	Ali Daba		2.78	1.06	
AD3_S1	Ali Daba		3.10	1.97	-
HI_2-3_1_88	Hara Idé 2		2.06	-	1.73
PBC_5885_L	Recent human		-	1.07	-
PBC_5885_R	Recent human		-	1.32	-
PBC_6434_R	Recent human		2.48	1.49	-
		Mean	*1.14*	*0.58*	*2.49*
Values for the rest of the Homo sapiens *sample*		*SD*	*0.39*	*0.21*	*0.26*
(n= 100)		*Min*	*0.30*	*0.15*	*2.04*
		Max	*1.95*	*0.98*	*3.23*

Supplementary Table S5: Details on the preservation and taphonomic state of the original African samples.

Sample	Region	Date	Overall preservation	Sediment infilling	Bone-matrix contrast	Diagenetic alteration	Contours of the structure studied
Jebel Sahaba	Nubia	Late Pleistocene	Very good	Moderate to heavy (coarse grains)	Good	Moderate (erosion of the outer bony surface)	Sharp (slight erosion of some outer aspect of VA)
El-Barga		Early-Mid Holocene	Medium to very good	Moderate to heavy (mixed grain size)	Medium to good	Sometimes eroded and/or carbonate crust	Sharp to partially eroded
Kadruka		Middle Holocene	Very good	Moderate to none	Very good	None	Sharp
Sphinx	Central Sudan	Early Holocene	Medium to very good	Moderate to heavy (mixed grain size)	Medium to good	Sometimes eroded and/or carbonate crust	Sharp to partially eroded
Fox Hill		Early-Mid Holocene	Medium to very good	Moderate to heavy (mixed grain size)	Medium to good	Sometimes eroded and/or carbonate crust	Sharp to partially eroded
Shaqadud		Middle Holocene	Very good	Moderate (coarse grains)	Very good	None	Sharp
Ghaba		Middle Holocene	Medium to very good	Moderate (mixed grain size)	Medium to very good	None to moderate erosion	Sharp
Abu Tabari	Sudanese Western Desert	Middle Holocene	Good to very good	Moderate (mixed grain size)	Good to very good	None to moderate erosion	Sharp to partially eroded
Wadi Shaw		Middle Holocene	Very good	Moderate to none	Very good	None	Sharp
Ali Daba	Horn of Africa	Middle Holocene	Medium to very good	Moderate to heavy (coarse grains)	Very good	None	Sharp
Hara Idé 2		Middle Holocene	Very good	Moderate (coarse grains)	Very good	None	Sharp
Mille-Logya		Middle Holocene	Good	Moderate (mixed grain size)	Good	None	Sharp
Gogoshiis Qabe		Middle Holocene	Very good	Moderate (mixed grain size)	Very good	None	Sharp
Shum Laka	Central Africa	Middle-Late Holocene	Very good	None to moderate	Very good	None	Sharp
PBC	South Africa	Present day	Very good	None	/	None	Very good

Supplementary Table S6: Details on the landmarking procedure.

Landmarks

1	Entry point of the posterior SCC into the vestibule (midline)
2	Y-junction of the cochlea (midline)
3	Entry point of the anterior SCC into the vestibule (midline)
4	Entry point of the common crus into the vestibule (midline)
5	Posterior entry point of the lateral SCC into the vestibule (midline)
6	Anterior entry point of the lateral SCC into the vestibule (midline)
7	Entry point of the cochlea into the vestibule (outer surface)
8	Apex of the cupula (outer surface)

Curve semi-landmarks

Landmarks 1-2	38 equidistant semi-landmarks (posterior SCC midline)
Landmarks 2-3	38 equidistant semi-landmarks (anterior SCC midline)
Landmarks 2-4	8 equidistant semi-landmarks (common crus midline)
Landmarks 5-6	38 equidistant semi-landmarks (lateral SCC midline)
Landmarks 7-8	48 equidistant semi-landmarks (cochlea outline)

SI References

Close. A. *et al*. *Prehistory of arid North Africa. Essays in honor of Fred Wendorf.* (Southern Methodist University, Dallas) 357 p. (1987).

Friedman *et al*. ***glmnet**: Lasso and Elastic-Net Regularized Generalized Linear Models.* R package (2025). https://glmnet.stanford.edu/

Kuhn *et al.* ***caret**: Classification and Regression Training*. R package (2024). https://github.com/topepo/caret/

Martin, N. *et al.* Enamel–dentine junction morphology reveals population replacement and mobility in the late prehistoric Middle Nile Valley. *Proc. Natl. Acad. Sci. U.S.A.* **122**, e2419122122 (2025).

Oksanen *et al.* ***vegan**: Community Ecology Package.* R package (2025). https://vegandevs.github.io/vegan/

Spoor, F. & Zonneveld, F. Morphometry of the primate bony labyrinth: a new method based on high-resolution computed tomography. *J. Anat.* **186**, 271–286 (1995).
