# Supplementary material for: Bony labyrinth morphology reveals deep population history and isolation among Homo sapiens in prehistoric northeastern Africa (44–3 ka)
Source: Nat Commun. 2026 Aug 12;17:8181. doi: 10.1038/s41467-026-76423-5 (PMC13470358; doi:10.1038/s41467-026-76423-5)
Supplement: Supplementary file 2 [file 41467_2026_76423_MOESM2_ESM.pdf]

Description of Additional Supplementary Files

File Name: Supplementary Data 1
Description: List of the individuals included, with details on the context, provenance, current location, and previous studies on the bony labyrinth.

File Name: Supplementary Data 2
Description: Detailed acquisition parameters of the original data reported in this study.

File Name: Supplementary Data 3
Description: Interactive html PCA plots of the shape analyses discussed in the main text (i.e., Figure 3)

File Name: Supplementary Data 4
Description: Raw landmark coordinates collected on the Nile Valley and recent South African samples

File Name: Supplementary Data 5
Description: Linear bony labyrinth measurements collected on the Nile Valley and recent South African samples

File Name: Supplementary Code 1
Description: R code used to conduct the analyses presented in this study.
