# Supplementary material for: Bony labyrinth morphology reveals deep population history and isolation among Homo sapiens in prehistoric northeastern Africa (44–3 ka)
Source: Nat Commun. 2026 Aug 12;17:8181. doi: 10.1038/s41467-026-76423-5 (PMC13470358; doi:10.1038/s41467-026-76423-5)
Supplement: Supplementary file 3 [file 41467_2026_76423_MOESM6_ESM.pdf]

nature portfolio

Corresponding author(s): Nicolas Martin, Isabelle Crevecoeur

Last updated by author(s): Jun 1, 2026

Reporting Summary

Nature Portfolio wishes to improve the reproducibility of the work that we publish. This form provides structure for consistency and transparency in reporting. For further information on Nature Portfolio policies, see our Editorial Policies and the Editorial Policy Checklist.

Statistics

For all statistical analyses, confirm that the following items are present in the figure legend, table legend, main text, or Methods section.

n/a	Confirmed	
☐	☒	The exact sample size (*n*) for each experimental group/condition, given as a discrete number and unit of measurement
☐	☒	A statement on whether measurements were taken from distinct samples or whether the same sample was measured repeatedly
☐	☒	The statistical test(s) used AND whether they are one- or two-sided<br>*Only common tests should be described solely by name; describe more complex techniques in the Methods section.*
☐	☒	A description of all covariates tested
☒	☐	A description of any assumptions or corrections, such as tests of normality and adjustment for multiple comparisons
☐	☒	A full description of the statistical parameters including central tendency (e.g. means) or other basic estimates (e.g. regression coefficient) AND variation (e.g. standard deviation) or associated estimates of uncertainty (e.g. confidence intervals)
☐	☒	For null hypothesis testing, the test statistic (e.g. *F*, *t*, *r*) with confidence intervals, effect sizes, degrees of freedom and *P* value noted<br>*Give P values as exact values whenever suitable.*
☒	☐	For Bayesian analysis, information on the choice of priors and Markov chain Monte Carlo settings
☒	☐	For hierarchical and complex designs, identification of the appropriate level for tests and full reporting of outcomes
☒	☐	Estimates of effect sizes (e.g. Cohen's *d*, Pearson's *r*), indicating how they were calculated

Our web collection on statistics for biologists contains articles on many of the points above.

Software and code

Policy information about availability of computer code

Data collection	The microCT data were processed using Avizo 9.5.0 (Thermo Fisher Scientific) and Biomedisa softwares. 2D measurements and landmark coordinates were obtained using Avizo 9.5.0. The meshes used for DSM analysis were processed with Avizo 9.5.0 and Paraview v5.6.2.
Data analysis	DSM computation was performed using Deformetrica v4.3 (https://www.deformetrica.org/). Statistical analyses were performed using R v4.2.2 and Rstudio v2025.05.0+496. The following packages were used: ade4 (v.1.7.22); plotly (v.4.11.0); Morpho (v2.11); pairwiseAdonis (v0.4.1); dispRity (v1.8).<br>The R scripts used in this study, adapted from previously published sources cited in the manuscript, are available as Supplementary Code 1. The scripts were developed for the specific datasets analysed here and may require adaptation to be reused. Some of the raw data required to run the scripts are not yet publicly available.

For manuscripts utilizing custom algorithms or software that are central to the research but not yet described in published literature, software must be made available to editors and reviewers. We strongly encourage code deposition in a community repository (e.g. GitHub). See the Nature Portfolio guidelines for submitting code & software for further information.

Data

Policy information about availability of data

All manuscripts must include a data availability statement. This statement should provide the following information, where applicable:

- Accession codes, unique identifiers, or web links for publicly available datasets
- A description of any restrictions on data availability
- For clinical datasets or third party data, please ensure that the statement adheres to our policy

Access to the original material should be addressed to the curators of the relevant curating institutions (see Supplementary Note S2). Access is subject to institutional approval and curation policies governing the human remains.
The µCT scan data and derived 3D models are available from the corresponding authors only with permission from the relevant curating institutions. Raw µCT data are also held by the curating institutions and may be accessed under their data access procedures for scientific research purposes. Requests must be directed to the curatorial contacts listed in Supplementary Note S2 and are subject to review and approval by the relevant institution.
Linear measurements and landmark coordinate data for the Middle Nile Valley and present-day South African individuals are provided in Supplementary Data S4. Equivalent datasets for other individuals are available from the corresponding publications cited in the text (i.e., refs8,40-44,89-91) or from the corresponding author subject to institutional approval.
Source data are provided with this paper.

Research involving human participants, their data, or biological material

Policy information about studies with human participants or human data. See also policy information about sex, gender (identity/presentation), and sexual orientation and race, ethnicity and racism.

Reporting on sex and gender	The archaeological assemblages analysed in this study represent pooled individuals, and biological sex could not be reliably determined for all specimens. Sex was therefore not considered in the analyses. Biological sex is mentioned only in Table 2, solely for informational purposes and only where available.
Reporting on race, ethnicity, or other socially relevant groupings	N/A
Population characteristics	N/A
Recruitment	N/A
Ethics oversight	Part of the material included in this study was excavated in the framework of several international archaeological missions in Africa, with the authorization and support of the responsible authorities and governments. Some of the material recovered from these excavations, including human remains studied in this work, are currently on temporary loan or permanent deposit in various European and American institutions for study, documentation and restoration, with all necessary licenses and permits issued by the responsible authorities and governments. Access to the collections was granted by the institutions in charge of the remains and all inquiry about access to the original material should be directed to them. See Supplementary Material Note S2 for further details on the excavations, exportation, study and current status of the original collections included in this study.<br>Access to the µCT acquisitions from the Pretoria Bone Collection was granted by the University of Pretoria, with approval of the Research Ethics Committee of the Faculty of Health Sciences (HUM017/1123).

Note that full information on the approval of the study protocol must also be provided in the manuscript.

Field-specific reporting

Please select the one below that is the best fit for your research. If you are not sure, read the appropriate sections before making your selection.

☐ Life sciences ☐ Behavioural & social sciences ☒ Ecological, evolutionary & environmental sciences

For a reference copy of the document with all sections, see nature.com/documents/nr-reporting-summary-flat.pdf

Ecological, evolutionary & environmental sciences study design

All studies must disclose on these points even when the disclosure is negative.

Study description	Northeastern Africa is a pivotal region for understanding modern human dispersals within and out of the continent. Yet, in the absence of ancient DNA and given the scarcity of morphological studies based on large, well-contextualized samples, the population history of this region remains poorly understood. By focusing on bony labyrinth morphology at this scale for the first time, our study provides novel insights into deep ancestry, isolation, and demographic transitions over the past 40,000 years. We focus on the bony labyrinth morphology (a highly reliable proxy for population affinities) of 148 individuals spanning the Late Pleistocene to Late Holocene and orginating from the Nile Valley, the Horn of Africa and Central Africa, with comparative African and Eurasian samples. By combining cutting-edge 3D imaging and morphometric methods with rare and often inaccessible collections, we generate a fine-

grained picture of northeastern African population dynamics across the Late Pleistocene and Holocene.
This study employed a comparative observational design using CT-derived morphological data from archaeological and comparative specimens. Individual specimens constituted the units of analysis, with each bony labyrinth contributing a single set of measurements (or landmark configuration). No experimental treatments were applied. Specimens were assigned to predefined groups based on site, period, and cultural affinities, and statistical analyses evaluated morphological variation among these groups.

Research sample	The sample included the pretrous bones/temporal bones of 148 individuals from Africa and Eurasia. Specifically, we included 68 individuals from Late Pleistocene to Late Holocene sites in the Middle Nile Valley region; 7 individuals from the Shum Laka (Cameroun) assemblage; 11 individuals from Holocene sites in the Horn of Africa; 4 Late Pleistocene fossils from diverse African sites; 21 Eurasian Pleistocene Homo sapiens individuals; 41 Neandertal specimens; and 30 present-day South African individuals
Sampling strategy	The individuals included in this study were selected to represent key temporal and geographic points across northeastern Africa, the broader continent and Eurasia, with the goal of documenting variation in the bony labyrinth to address the population/archeological questions articulated in the manuscript. All data available for the regions and periods studied were included. Key comparative samples were selected with respect to the research questions.
Data collection	The 3D models of the specimens were obtained through segmentation of X-ray micro-computed tomography scans. Original scans were performed at various institutions, depending on the available facilities near the curating institutions (see details in SI Data S2). Scans of the remaining individuals were provided to N.M. and I.C. by the curating institutions in charge of the remains. All segmentation of the scans and sub-sequent measures/analyses of the original data was performed by N.M.
Timing and spatial scale	Given that all the investigated specimens were either dry crania or fossils, the timing and spatial scale of the data collection is not affecting the analysis performed in the current study.
Data exclusions	No available data relevant for the regions and period of time studied here was excluded from the analysis.
Reproducibility	The methods employed in this work have been previously tested and variously published, and fully demonstrated to be reliable and replicable. Intra-observer error tests were performed for the segmentation and landmark digitization protocols. Both revealed negligible errors.
Randomization	To ensure that the results were not dependent from sample size, disparity metrics were computed on the bootstrapped distributions of the groups.
Blinding	Blinding was not relevant to this study because no experimental treatments or any clinical trial with participants and groups. Analyses were based on objective morphometric measurements obtained from CT-derived anatomical structures.

Did the study involve field work? ☐ Yes ☒ No

Reporting for specific materials, systems and methods

We require information from authors about some types of materials, experimental systems and methods used in many studies. Here, indicate whether each material, system or method listed is relevant to your study. If you are not sure if a list item applies to your research, read the appropriate section before selecting a response.

Materials & experimental systems

n/a	Involved in the study	
☒	☐	Antibodies
☒	☐	Eukaryotic cell lines
☐	☒	Palaeontology and archaeology
☒	☐	Animals and other organisms
☒	☐	Clinical data
☒	☐	Dual use research of concern
☒	☐	Plants

Methods

n/a	Involved in the study	
☒	☐	ChIP-seq
☒	☐	Flow cytometry
☒	☐	MRI-based neuroimaging

Palaeontology and Archaeology

Specimen provenance	Individuals from the Nile Valley were recovered from various sites from present-day Sudan and dated to the Late Pleistocene (Jebel Sahaba), Early Holocene (El-Barga, Sphinx, Fox Hill), and Middle-Late Holocene (El-Barga, Fox Hill, Ghaba, Kadruka 1, Shaqadud, Abu Tabari, Wadi Shaw). Central African individuals originate from the Shum Laka cave, in Cameroun. Individuals from the Horn of Africa originate from various sites dated to the Holocene in Djibouti (Ali Daba, Hara Idé 2), Ethiopia (Mille-Logya), Somalia (Gogoshiis Qabe), and Kenya (Lothagam). Late Pleistocene African fossils originate from assemblages in Egypt (Nazlet Khater 2), Democratic Republic of Congo (Ishango), South Africa (Hofwmeyr), and Somalia (Guli Waabayo). Eurasian Pleistocene Homo sapiens and Neandertal specimens originate from sites in France (Cro-Magnon, Abri Pataud, Chancelade, Abri Lafaye, Rochereil, Laugerie-Basse, Le Moustier, Les Pradelles, Pech de l'Azé, La Quina, La Ferrassie, La Chapelle-aux-Saints, Arcy-sur-Cure, Peti-Puymoyen), Belgium (Spy, Goyet, Engis), Czech Republic (Zlatý kůň), Spain (Sima de los Huesos, Cova Negra), Syria (Dederiyeh), and Israel (Kebara, Amud). The present-day South African individuals originate from the Pretoria Bone Collection.

Specimen deposition	Original specimens examined in this study are curated at various African, European and North American institutions, as detailed in Supplementary Information Note S2, and compiled in Supplementary Data S1. Comparative specimens measured directly for this study are housed at the University of Pretoria, the Muséum national d'Histoire naturelle (Paris, France), the Neanderthal Museum digital archive, the Smithsonian Institution, the Périgord Museum of Art and Archaeology, the Natural History Museum of Montauban, the Institue of Natural Sciences (Brussel, Belgium), and the French Museum of National Antiquities. All additional comparative data were obtained from the published literature cited in the main text, where specimen provenance and repository information are provided.
Dating methods	No new dates are provided in the present study.

☐ Tick this box to confirm that the raw and calibrated dates are available in the paper or in Supplementary Information.

Ethics oversight	Access to the archaeological collections was granted by the institutions in charge of the remains. See Supplementary Material Note S2 for further details on the excavations, exportation, study and current status of the original collections included in this study. No further ethic approval was sought for these fossil specimens.<br>Access to the µCT acquisitions from the Pretoria Bone Collection was granted by the University of Pretoria, with approval of the Research Ethics Committee of the Faculty of Health Sciences (HUM017/1123).

Note that full information on the approval of the study protocol must also be provided in the manuscript.

Plants

Seed stocks	N/A
Novel plant genotypes	N/A
Authentication	N/A
