# Supplementary material for: Bony labyrinth morphology reveals deep population history and isolation among Homo sapiens in prehistoric northeastern Africa (44–3 ka)
Source: Nat Commun. 2026 Aug 12;17:8181. doi: 10.1038/s41467-026-76423-5 (PMC13470358; doi:10.1038/s41467-026-76423-5)
Supplement: Supplementary file 4 [file 41467_2026_76423_MOESM7_ESM.pdf]

nature portfolio

Peer Review File

Bony labyrinth morphology reveals deep population history and isolation among *Homo sapiens in prehistoric northeastern Africa (44 – 3 ka)*

Corresponding Author: Dr Nicolas Martin

This file contains all reviewer reports in order by version, followed by all author rebuttals in order by version.

Version 0:

Reviewer comments:

Reviewer #1

(Remarks to the Author)
This study explores the morphological variation of bony labyrinth morphology in African and western Eurasian human populations spanning the last circa quarter million years. Most original data derive spatially from across the Nile Valley and temporally from contexts spanning the Holocene and terminal Pleistocene. The manuscript is difficult to follow because no clear hypotheses are provided at the outset, the aims are rather broad, and the discussion provides interpretations that go beyond what the data can properly address. While the Introduction provides the general motivation for conducting the exploratory study, the first two of the three aims are not sufficiently justified or addressed with the analyses undertaken. In addition, there are some study design and methodological choices that need to be addressed before the manuscript can be considered for publication. Nevertheless, the manuscript excels in that (1) the original data is extremely valuable given its relative scarcity, (2) it applies three analytical methods (2D morphometrics, 3D-GMM, and DSM) to one anatomical element, and (3) provides careful analysis of pathological features, likely often overlooked in archaeological/paleontological studies of the bony labyrinth. I expect several revisions will be necessary to bring this manuscript to a publication stage that is commensurate with this journal and accessible to its wide readership.

The points below outline some of the major and minor areas of improvement in revising the manuscript.

Major points:
1. Study Design, justification: the primary reason the manuscript is difficult to follow is that it is attempting to address several topics at once with variable success relative to the data available, including: (1) human population structure in Africa since the Late Pleistocene, (2) population (dis)continuity, (3) comparison of inferences on population (dis)continuity in the Nile Valley from the original bony labyrinth results to previous dental studies, (4) morphological bony labyrinth differences between foragers and food producers, and (5) the effect of pathologies on bony labyrinth form. The Introduction and Discussion further introduce (6) climate variability as a corollary. The aims that are actually articulated (lines 123-127) are roughly aligned with Topics 3-4. Topics 1-2 likely stem from questions that were set out when the petrosae were sampled for aDNA, but more intractable with the samples examined here (and including the comparative data) and the methods employed. Topics 3-5 are more directly tied to the original samples and would benefit from more information on their archaeological context. Limiting the paper to topics 3-4 would make for a much stronger manuscript. Overall, because none of the topics is tied to explicit hypotheses in the Introduction, the entire manuscript is difficult to follow and reads as too ambitious and speculative despite some very interesting analyses.

2. Study Design, aim 2 and comparative samples: while it could be useful to include a recent South African sample and Neanderthal samples in a comparative context, there isn't a clear justification for doing so. For example, how is a recent South African population relevant here? In other words, why is it useful to compare recent South Africans to Late Pleistocene-Holocene populations from Northeastern Africa? In the same vein, why should Neanderthals be included? Is there some relevance, for example, to the back-to-Africa Neanderthal discussion from population genetics or Nubian technology discussions from archaeology? Or is it simply to contrast the Neanderthal-specific bony labyrinth morphology? Similarly, how are samples from Cameroon relevant here? Justifying the inclusion of the samples is important because it will affect the results. For example, calculation of momenta in the DSM analysis might be strongly biased with the recent South African sample. For specialists in the field, this manuscript reads largely as an opportunistic exploratory study that takes the

scans of petrosae that were sampled for aDNA to simply put into comparative context with other data available, then work a story around it. That in itself is absolutely fine, but does not make for a clear hypothesis-driven paper commensurate with the quality of publications in this journal. Perhaps there were indeed hypotheses at the outset, in which case they should be clearly articulated (see comment 1, above). What might make the most sense is to focus on aim 3 and limit the analyses spatially to the Nile Valley and Horn of Africa and temporally to the terminal Pleistocene and Holocene. Other samples could be part of a separate manuscript with additional data to property address aims 1-2.

3. Study Design, use of bilateral labyrinths: the use of two labyrinths for one individual (i.e. the right and left inner ears) in the analyses in only the cases where they are available raises some important implications that must be further explored to ensure robust interpretation of the results. Unless otherwise unspecified, each labyrinth is essentially being treated as one case in all analyses, even when two bilateral labyrinths are from the same individual case/specimen. This will produce a bias in terms of appropriately quantifying variation of groups because some individuals are considered twice and others only once when bilateral labyrinth data is unavailable. More concerning is that a brief inspection of the 2D measurement values and the PCA distribution shows that the differences within individuals might be significant. For example, the Supplementary Data S2 corresponding to Fig. 2A in the main text can be used to explore the fact that in some cases, e.g. PBC_5089, the L and R plot relatively distant from each other, with several other cases in between. This implies that the variation within individuals can be greater than variation between individuals and populations: note, for example that PBC_5089R plots within the convex hulls of two other groups, while PBC_5089L does not. One solution is to simply take the average values of the L and R for each case so as to not bias the variation within and between groups, which is the primary aim of analysis in this study. It would then also be necessary to reassess whether the remaining sample sizes of true cases is sufficient for the statistical analyses employed, with justification of parametric and non-parametric methods. At the same time, it could be interesting to look at the bilateral data separately and assess, for example, whether the degree of asymmetry is larger in the outlier cases (see also comment 7 on pathologies, below). Again, a careful evaluation of sample sizes is necessary here depending on the statistics employed.

4. Study Design, group composition and sample sizes: It is difficult to follow how groups were delimited and what the sample sizes were in each analysis. For example, Table 1 lists 10 sample groups (5 original, 5 comparative), while Figure 1 implies having 12 groups (7 original, 4 comparative, and 1 overlapping), and Figure 2 implies having 13+ groups (6 original, 6 comparative + 2 for 2D analyses, 1 overlapping, plus 2 outlier groups), etc. At a minimum, Table 1 should reflect the groupings most commonly used in the analyses so that sample sizes can be assessed efficiently by the reader (see also comment 3, above, on case duplicates). The Nile Valley groups might be well sampled, but some assessment of this should be presented. The Horn of Africa and Central Africa samples are more problematic. For the Horn of Africa group, the single Gogoshiis Qabe case is spatially and temporally more distant to the others, so some justification of inclusion is necessary. For Central Africa, it is not clear why the samples should be treated as one group when the temporal span is almost as large as that of the Holocene Nile Valley samples, which comprise 6 groups. Underlying these decisions is both a statistical question in data analysis as well as an evolutionary question on population [dis]continuity, the latter of which forms a large part of the discussion and interpretation of the manuscript.

5. Discussion, PCA results: In describing PCA results, avoid terminology that suggests the analysis is associated to, or is itself, a discriminant or classificatory analysis or that there are clear boundaries (e.g. line 133: “distinguishes,” line 134: “separation,” line 138: “differences,” Supplementary Note S1: “clear distinction”, etc.). First, it is clear that there is substantial overlap between the different groups and, to a lesser degree, between taxa, so it would be more appropriate to refer to general patterns in PCA space. Even so, the Discussion might still be difficult to follow with reference to the figures. To better appreciate the trends, one possible form of displaying the PCA result is to delineate the convex hulls and only use the symbol for the mean of each group in the main text’s Fig. 2, referring to Supplementary Data S2 for further details. Secondly, terminology that suggests discrimination between groups in PCA results is particularly confusing because bgPCA and PERMANOVA are also employed. Thirdly, since much of the discussion relies on patterns and inferences from the PCA, identifying whether the sample sizes are sufficient to do so should be explored (e.g. getMeaningfulPCs() function in Morpho; see Bookstein 2014, Measuring and reasoning: numerical inference in the sciences. Cambridge University Press).
6. Discussion, differential preservation of population history: The argument that the bony labyrinth is more conserved than teeth (lines 213-226) appears to be based on relative measures of disparity, but it is not clear from this Discussion whether the different dental and bony labyrinth datasets can be properly compared. Particularly since this is reiterated as a conclusion of the paper, more work is necessary to substantiate the claim. For example, were the same individuals and populations sampled in previous dental studies also sampled in this study? Were the analytical methods similar?

7. Discussion, congenital pathologies and climate: this section (lines 290-373) is a substantial portion of the discussion and, while indeed stimulating, requires more context. For example, some indication of the archaeological background and taphonomic state of each sample should be included (e.g. as supplementary information) in order to rule out the possibility that some of these features are due to taphonomic effects (particularly for the assessment of EVA). The taphonomic state is also important for understanding possible variation in calculating the HMH for segmentation and the resulting values derived for the subsequent 2D, 3D-GMM, and DSM analyses (see also comment 10, below). The sociocultural interpretations of the archaeological record are also important because endogamy is assumed here to be the result of changing climate conditions (line 347) but could also be associated with behavioral (social, cultural, economic) practices, or both. The general impression is that the section on pathologies could be another paper altogether and better when more fully explored.

8. Methods, 2D linear comparative data: the original data presented in Table S1 appears to be reported with values to about the 13-14th decimal. This should be consistent in order to avoid subtle differences in the analyses that are simply due to rounding. More importantly, since the previously published measurements are unlikely to be to the 13-14th decimal, the original data should be rounded to the lowest common decimal point in order to avoid any inadvertent false interpretation

due to rounding. For transparency, all specimens, including those derived from published data, should be compiled in Table S1. In addition, since the study is likely using PCA on both the variance-covariance matrix (in the case of 3D-GMM and DSM data) as well as the correlation matrix (in the case of 2D data), this should be confirmed and stated.

9. Methods, landmark post-processing: landmark data pre-processing is described simply as "Procrustes superimposition." Since the shapes package provides the option of both GPA and OPA, specify for a non-specialist audience that GPA was applied. More importantly, specify whether the semilandmarks were slid or resampled in any way and justify why or why not given the different approaches in the literature and in the cited works. Note that this affects how landmarks are referred to in line 461 as "pre-defined" points, which would only be true in the sense of landmark correspondence, i.e. pre-defining the number of landmarks defining a homologous region.
10. Methods, intraobserver measurement error test: the measurement error employed is insufficient. In general, a Procrustes ANOVA would be preferable to understand whether the variation observed between repeated measures exceeds the variation between groups under investigation. In addition, the interobserver PCA presented here is helpful only to get an idea about the possible impact of measurement error, but a better representation would be one that takes the PCA results of the full dataset and then projects the repeated measures onto that space. Still, note that this would only present a portion of total variation (e.g. along two axes in a 2D PCA plot). Related to the comment above, it might make sense to limit the error test to the Type 1-3 landmarks rather than at the inclusion of Type 4-6 landmarks (sensu Weber & Bookstein 2011), depending on how the Type 4-6 (semi)landmarks were processed. In addition, specify whether a measurement error was undertaken for calculating the HMH and undertaking the segmentation procedure. This is a difficult and extremely time-consuming task but is important because it directly affects all derived data values. Images of the segmented labyrinths in the supplement suggest that there is substantial variation in the degree of sediment found inside (i.e. taphonomy) and the degree of manual segmentation. If no error test was conducted to test segmentation variability, then it is important to state that variation is assumed to be negligible and, at minimum, some qualitative assessment would be necessary to support such assumption.

Minor points:
11. Throughout the manuscript, are the terms "hunter-gatherers," "foragers," and "hunter-fisher-gatherers" interchangeable? If so, simplify to "foragers" or specify the different designations.
12. Throughout the manuscript, are surface registration and DSM used interchangeably? Note that some methods referred to as surface registration in the literature differ from DSM (e.g. Roeding et al.'s implementation of Schlaeger's: https://doi.org/10.1002/ajpa.24642), so it is important to clarify.
13. Line 89: first clause is vague (i.e. what does "central role" and "dynamics" refer to?) Clarify or remove.
14. Line 92: word choice; consider changing "exploitable" to neutral term or more descriptive word (e.g. endogenous)
15. Line 92: a citation is needed here or further explanation to which of the samples included in the study were sampled for ancient molecules, alongside the success rate of endogenous molecules
16. Lines 95-96: this sentence is confusing since the first clause implies that the Middle Nile Valley region is an exception to the scarcity of aDNA described in the previous paragraph, yet the last clause says that no molecular data is available. Rephrase for clarity.
17. Line 95: clarify what Middle Nile Valley refers to and/or indicate in Fig. 1
18. Lines 105-107: rephrase for clarity: as written, the gap appears to refer to dental studies; in addition, provide brief description of bony labyrinth for the wide readership of this journal
19. Lines 112-113: clarify what "…studied into much detail" refers to or rephrase
20. Lines 115: change "propose using" to "use"
21. Lines 115-116: disambiguate "population complexity" with additional details, citation(s), or by rephrasing
22. Line 116: disambiguate "reference point" or rephrase
23. Lines 117-122: edit for conciseness; details of fossils will be in Table 1, so there is no need to name them here, particularly for a general science audience
24. Figure 1C: specify what the bars and dashes indicate (i.e. date ranges reported in Table 1)
25. Figure 2 description: spell out SCC in the first instance for ease of reading without reference to main text
26. Figure 2 legend: spell out words when there is space to do so; specify here and throughout manuscript what "MIS" refers to (clarify to a non-specialist readership)
27. Figure 2A and 2B: consider moving the 2B inset down to the lower left corner in order to show that you've also used Levantine samples (i.e. Qafzeh/Skhul). Add these and the pre-Neanderthals to the map in 2A.
28. Figure 2A, 2C: it is better to indicate surface and wireframe deformations as number of standard deviations from the mean than at "extremes" along the axes, particularly when the axes are not labeled at their terminus
29. Figure 2A, 2C: given the distribution of the data, it would be more interesting to present the surface and wireframe deformations at the "extremes" of the quadrant corners (e.g. 2A: the "extreme corner" of quadrant comprising positive PC1 and negative PC2 to show the shape of the quadrant where the majority of Neanderthals plot; the corner of positive PC1 and positive PC2 where the majority of outliers plot)
30. Figure 2B: identify in figure description what the biplot is; move ASC%R label within the biplot to avoid confusion
31. Figure 2 and Table 1: avoid confusion by using the same labels for the samples
32. Results & Discussion: avoid using the term "fall" for describing the positioning of cases in PCA space. Use "plot" or other descriptive terminology with respect to eigenvalues/eigenvectors.
33. Lines 162-163: it is unclear how the disparity values for Horn of Africa and Central Africa samples are comparable to the recent South African and EUP samples. Check if this is correct or clarify.
34. Line 242: this it he only instance where the term "archaic" is used and, without further context, vague. The sentence could rephrased and simplified or further contextualized.
35. Line 262: disambiguate "opposite affinities." Do you simply mean that they plot on both negative and positive parts of PCs? It would be better to describe their affinity to other individuals (e.g. ProcD, MahalanobisD, MST, etc.) or groups.

36. Lines 418-420: explain that these are parameter range values, with specific values dependent on specimen
37. Lines 424-425: clarify how Avizo and Biomedisa were used in conjunction, as well as what "manual corrections" refers to
38. Lines 426-427: provide justification for smoothing and padding value
39. Line 430: change "plan" to "plane"
40. Lines 430-431: the works cited by Spoor et al. do not use Avizo for defining the transverse and sagittal planes, so be specific about how this was done (e.g. defining the transverse plane relative to the lateral canal?). Also clarify the interpolation step of this function.
41. Supplementary Data S2: ensure consistency of the group names used in the main text and other parts of the manuscript. Acronyms used here can be deciphered in most instances but might not be obvious to non-specialists. For example, it might not be clear that HHEID in the 2D PCA plots refers to the Pre-Neanderthals in other parts of the manuscript.
42. Line 437: clarify that "largest dataset possible" refers to maximizing comparative samples from Africa, the Levant, and western Eurasia (e.g. eastern Eurasia is not represented)
43. Line 441: specify what each study "inspired" (and see major comment 9, above)
44. Line 461: specify that "pre-defined" refers to the number of landmarks.
45. Line 464: justify the selection of 30k triangles in the decimation procedure
46. Supplementary Note S1: it should be referenced in the main text
47. Supplementary Note S1: Homo sapiens should be italicized consistently (it is not prior to referring to Qafzeh and Skhūl)
48. Figure 4: specify in the figure description what the specific pathologies are for C-E.
49. Lines 504-505: specify how the transverse/axial views were standardized across samples

--
Hugo Reyes-Centeno
Assistant Professor, University of Kentucky
Fellow, Swedish Collegium for Advanced Study

Department of Anthropology
211 Lafferty Hall, Lexington, KY 40506 USA
William S. Webb Museum of Anthropology
1020 Export St, Lexington, KY 40504 USA
heva.as.uky.edu | educelab.engr.uky.edu

Linneanum
Villavägen 6c, SE-752 36 Uppsala, Sweden
swedishcollegium.se | centerforthehumanpast.se

Co-Reviewer: Stylianos Koutalis, University of Tübingen, Early Career Researcher

Reviewer #2

(Remarks to the Author)
I co-reviewed this manuscript with one of the reviewers who provided the listed reports. This is part of the Nature Communications initiative to facilitate training in peer review and to provide appropriate recognition for Early Career Researchers who co-review manuscripts.

Reviewer #3

(Remarks to the Author)
The manuscript "Bony labyrinth morphology reveals deep population history and isolation in prehistoric northeastern Africa" is a very interesting study that tackles an important question about population history in northeastern Africa through the analysis of bony labyrinth morphology. The dataset is excellent, the methodological approach is solid, and the results are not only a great contribution to the anthropology and archaeology of northeastern Africa, but also potentially valuable for regions where ancient DNA is unavailable. The paper is generally well organized and clear, and the results have strong potential to advance our understanding of the morphological evolution of human populations during the Late Pleistocene and Holocene. Before being considered for publication, the manuscript would benefit from several clarifications and changes before it can reach its full shape.
Main comments
1. Context and framing: The introduction could do more to situate the study within existing literature from the region. Previous work using dental morphology in the Nile Valley (e.g., Irish 2005; Irish & Usai 2021; Martin et al. 2024, 2025) has already established key patterns of continuity and replacement. It would help readers if the authors briefly summarize what those studies showed and explain how the present work expands on them. Similarly, a short paragraph outlining the main archaeological expectations for each region would make the objectives and hypotheses much clearer.
2. Geographic and authorship imbalance: All authors are affiliated with European or North American institutions, despite the study's focus on African materials. This is increasingly recognized as an issue in our field. It would be appropriate to acknowledge the role of local collaborators and curators more explicitly, and to provide transparency about data access and permits—also mentioning if African researchers were involved in field or curatorial work.
3. Sampling and representativeness: The sample from the Nile Valley is strong and well distributed chronologically, but the comparative material from the Horn of Africa and Central Africa is very small (n = 7–10). This limits what can be inferred about those regions. Contrary to what other reviews might say, I think it is a good idea to include those samples despite their

small number; however, the authors should explicitly acknowledge this limitation and frame those observations as preliminary or exploratory.
4. Comparative framework/Neanderthal sample: Including Neanderthals in the same PCA and DSM spaces as Homo sapiens greatly inflates interspecific variance, which dominates the first principal component and makes it harder to discern the subtler population-level differences among H. sapiens. In the current analysis, PC1 is almost entirely driven by features distinguishing Neanderthals (smaller and lower posterior semicircular canal, narrower anterior canal, larger lateral canal), which overshadows the within-human variation the paper aims to interpret. This design choice compresses H. sapiens diversity and partly masks contrasts that are central to the study—such as those between Late Pleistocene/Early Holocene foragers and Neolithic producers in the Nile Valley. Because the study's main question concerns population structure within Africa, the Neanderthal sample should be excluded from the main statistical analyses. European Upper Paleolithic H. sapiens specimens provide a sufficient comparative outgroup without distorting the variance structure. This change would also improve the interpretability of downstream analyses (bgPCA, PERMANOVA, and disparity metrics), all of which are computed from PCA axes currently dominated by archaic–modern separation. Re-running these on H. sapiens only would make the distances and group classifications more meaningful and the discussion of the results more deeply connected to the aims of the study. The same applies to the allometric regressions, where results should be confirmed in the H. sapiens-only dataset to ensure that any size effects are not driven by interspecific differences. Importantly, the "outlier" Holocene individuals identified as morphologically "Neanderthal-like" because of shortened or thickened canals should remain outliers even in a H. sapiens-only analysis. Demonstrating this explicitly would strengthen the argument that their unusual morphology is truly exceptional within H. sapiens, rather than a by-product of including Neanderthals in the same morphospace. A sentence in the Discussion could clarify this: "Because our research question concerns intra-H. sapiens variation, we focus on analyses restricted to modern humans; ordinations including Neanderthals are presented in the Supplementary Information for reference." The figures would also benefit from showing both versions: the PCA including all taxa, and a second set with H. sapiens only, using the same axis limits for direct comparison.
5. Intra-observer error: The authors present a PCA but it would be more helpful to see an statistic testing of those differences are significant or not for each method used in the study (landmarks, measurements, deformation-based).
6. Presentation of results: The text mentions that all analytical approaches yielded "similar results" but it never specifies what those common patterns are. A short synthesis at the beginning of the Results section—summarizing the general patterns of continuity, discontinuity, and outliers observed across the three methods—would help orient readers before the detailed descriptions of the results obtained through each method. In addition, the figures could be made clearer and more self-explanatory:
o Figure 1: Please create a new figure for the variables displayed in Figure 1B. At the moment they are too small and mixed with inforamtion on geographic and chronological information of the samples.
o Figure 2: add concise labels or inset diagrams summarizing what PC1 and PC2 represent biologically (e.g., variation in posterior canal height, anterior canal width, cochlear coiling). Consider labeling or highlighting the 13 "outlier" individuals in the PCA plots and marking Nile Valley time periods with different symbols or ellipse outlines for clarity. As it is now, since there are many groups, the interpretation is a bit confusing.
o Figure 3: include confidence intervals and sample sizes for the disparity metrics, and note directly in the caption that small n values for the Horn and Central Africa make these estimates exploratory.
o Figure 4: indicate whether the inner-ear pathologies (EVA, CNC stenosis, fistulas) were measured or scored as present/absent, and show the diagnostic thresholds (e.g., ≤2 mm for EVA at the operculum, ≤1.8 mm for CNC) so the reader can understand the criteria.
7. Novel contribution: At several points the discussion reads as confirmation of patterns already described in the dental record. The paper would be stronger if it emphasized what new information the results of the bony labyrinth data adds—perhaps a deeper temporal signal, a different sensitivity to substructure, or evidence of evolutionary constraint compared to more plastic traits.
8. Pathological individuals: The section on individuals with "Neanderthal-like" labyrinth morphology is fascinating but needs more caution. It is not clear how "pathological" was defined or how consistent these diagnoses are across individuals. Some of these differences could reflect taphonomic deformation or natural variation rather than congenital syndromes. Please clarify the diagnostic criteria and consider alternative explanations, including de novo mutations or random developmental variation.
9. Interpretation of evolutionary processes: Some claims about isolation, endogamy, and fixation of mutations are currently too strong given the available evidence. These should be presented as possible scenarios rather than established facts. A short limitations paragraph at the end of the discussion would also strengthen the credibility of the paper.
Minor points
• In the introduction, adjust the phrasing "we propose using the Nile Valley as a reference" to "we use the Nile Valley as a reference."
• Also in the final paragraph of the introduction, explicitly state that the dataset includes individuals with pathological features and justify why they were included.
• Indicate whether fistulas and other inner-ear conditions were measured or simply recorded as present/absent.
• Figures should specify the percentage of variance explained by each axis and use more clear symbols for region or chronology.
• A few minor typos (e.g., "to to") and some small inconsistencies should be corrected.
• Supplementary data 1 and 3: when I download them, they are PDFs, but as a table with multiple columns it should be presented as xls or csv.
Overall assessment: This is a strong and potentially influential paper. The dataset is unique, and the integration of morphometric and pathological evidence is original. With some reframing of the introduction, clearer statements of limitations, a small redesign of the comparative framework regarding Neanderthal samples, and a more cautious interpretation of the pathological cases, the manuscript could make a significant contribution to the field. I recommend major revision.

Reviewer #4

(Remarks to the Author)
This paper is a very interesting study on morphological approaches to investigate population history and variability in northeast Africa. It specifically, and convincingly, demonstrates the usefulness of bony labyrinth morphology to complementing aDNA and other morphometric analyses (dental morphology) and it thereby opens new possibilities where the two former approaches are not fully available.
It involves 148 human individuals from a wide range of geographical locations, including Nile Valley, Horn of Africa, European, South and central African specimens, from late Pleistocene to late Holocene sites, covering a time span between 44 to 3 ka, i.e. from both forager and food-producing societies.
The article is very thoroughly researched and substantiated on the basis of past and current research results. The data and results are comprehensive and well-presented.
The results would support the authors' conclusions regarding, for example, the persistence of forager-related population groups well after the transition to Neolithic lifeways and the cohabitation of "two biologically distinct populations", at least in some areas of the Eastern Sahara margins. Moreover, the authors suggest that there was a high degree of "biological discontinuity at the Neolithic transition in the region", i.e. "the arrival of food-producing practices in the Nile Valley through migrations starting 8000 years ago."
While this interpretation generally requires more discussion, the result would prima facie support the idea of non-African origins of food-producing techniques, i.e. a knowledge import of food-producing live-ways from western Asia via human migration, parallel to the direct import of domesticates of sheep, goats and cattle, as well as cultivated plants around 8000 years ago. There are a number of relevant studies which are not cited, but which may be useful in the context, e.g.:
Vermeersch, P.M., Linseele, V., Marinova, E., Van Neer, W., Moyersons, J. and Rethemeyer, J. 2015. Early and Middle Holocene human occupation of the Egyptian Eastern Desert: Sodmein Cave. African Archaeological Review 32: 465– 503.
Xia X, Qu K, Wang Y, Sinding MS, Wang F, Hanif Q, Ahmed Z, Lenstra JA, Han J, Lei C, Chen N. Global dispersal and adaptive evolution of domestic cattle: a genomic perspective. Stress Biol. 2023 Apr 18;3(1):8. doi: 10.1007/s44154-023-00085-2
However, this reviewer sees two interrelated principal problems with the interpretation of the analysis results that the authors may want to consider before such wide-ranging conclusions are proposed.
One is that there is a wide gap in the regional representation of the 148 individuals included, and that there is no material from the Lower Nile Valley and adjacent regions. The sample only includes one specimen from Egypt (Nazlet Kater 2), which is obviously not representative for this part of Northeast Africa, and there are no Lower Nile Valley specimens from the Holocene period at all. While this reviewer appreciates the difficulties with working on human remains from modern excavations in Egypt, there are plenty of relevant specimens available for modern analysis in overseas museums and collections. These should be included in a study of such a broad research scope given that the northern Nile Valley, Nile Delta and Red Sea coast may well have been the key region of potential contact for early human groups using food-producing techniques with local African populations in the first place. What if those individuals who were of a distinctly non-African descent had no knowledge of food production? Or what if local Africans brought food-production to Africa themselves?
The authors of this study do emphasise the at times high degree of population variability in one and the same region or site (e.g. Shaqadud) and on the same chronological trajectory as well as "rare and localized instances of potential admixture or continuity between the last foragers and the earliest food-producing groups". In particular, those two individuals at Shaqadud, having been buried close to each other within a close temporal relationship but very different ancestry are interpreted as a possible result of the "cohabitation of two distinct biological populations". This reviewer would be more cautious to speak of populations when individuals are involved.
The other, related, issue is hence the interpretation of a limited number of individuals from a variety of geographically dispersed sites in the context of such significant socio-cultural processes as the Neolithic transition in Northeast Africa, which probably took place over more than a millennium. In view of the high degree of mobility of early Holocene groups inhabiting this region, there is also the possibility of local African forager groups in Northern Egypt expanding their scope of interest to areas in the Southern Levant and thereby adopting food-producing techniques themselves. Human migration, especially of the disorganised variety, is always a possibility when it comes to the transfer of knowledge, but it is rarely a one-way road when human populations had not adopted a sedentary life-style in the first place.
There are indeed numerous avenues to interpret the research results achieved in this interesting study and it would be helpful if these were sufficiently considered in the text, given the paper's title and key observations.
On the other hand, given the lack of material from the Lower Nile Valley, it may perhaps be prudent to reduce the interpretative scope of the paper.

Version 1:

Reviewer comments:

Reviewer #1

(Remarks to the Author)
This revision has resulted in a much improved manuscript, with additional analyses that help to support the robusticity of the results. In particular, one of the central aims—testing the differential population history signal of EDJ and bony labyrinth morphology in the Middle Nile Valley populations—has been well expanded. Additional points arising from revisions, as

well as points left unaddressed, are outlined below.

Major points:
1. Study Design, justification: The introduction is much improved, providing three key aims for the study in the penultimate paragraph. The second aim is unclear with regards to what "elsewhere" refers to. Is it referring to Horn of Africa, Central Africa, and South Africa, i.e. sub-Saharan Africa? Is the sampling justification then related to the third aim of assessing whether phenotypic patterns correspond to climate change and the hypothesized "ghost Green Saharans?" The third aim gives the impression that there will be an explicit analysis of samples from humid versus arid phases, but this is missing from the study design. If this is retained as a central aim, some solutions include adding paleoclimate markers to the timeline in Figure 1B, the designation of humid- versus arid- derived samples in Table 1, and additional/modified corresponding exploratory and statistical analyses. Otherwise, this would instead be part of the Discussion rather than a central aim.

2. Study Design, comparative samples: the revisions have adequately explored the effects of excluding the Neanderthal sample. Similarly, it would be beneficial to test the effect of removing the recent (Late Holocene) South African sample or, at minimum, resampling that group to test the effects of disproportionate sample size (e.g. results from Table S1, Homo sapiens sample results in Table S4, results of Figure S7, S13, etc.). Regarding the calculation of momenta, the point that is still unaddressed is whether reducing/excluding the South African sample will have an effect. The authors, in their rebuttal, have a clear hypothesis that "…such effects are not expected to meaningfully alter the resulting momenta or their interpretation…." Now it is time to test that hypothesis to further show that the results are robust.

3. Study Design, use of bilateral labyrinths: the added Figure S19 is very useful for visualizing variation within individuals. It is still unclear whether each labyrinth is being treated as one case in the other analyses e.g. OLS, PERMANOVA, etc. For example, it is unclear whether sample size has an effect on those results and to what degree this is affected by the inclusion of individual cases represented twice within each group. As an example, let's say a group comprises 5 individuals. In this sample, each individual can be counted twice (left AND right ears each) such that N=10. Alternatively, they could be counted only once (left OR right ears OR a mix thereof) such that N=5. The methodological decision is likely trivial with large sample sizes (e.g. recent South Africa) but could be consequential for small sample sizes (e.g. archaeological samples). One solution could be to resample the left and right data to test the effect on statistical results and/or to specifically account for asymmetry. In addition, a rarefaction analysis could be applied to downsample groups with larger sample sizes and/or duplicates (right AND left) of the same individuals. At the heart of the matter is whether asymmetry differs between groups and whether these differences could be variable spatiotemporally and associated to differential behavior: see, e.g. work by Ward et al. 2022 (AJPA: https://doi.org/10.1002/ajpa.24465) on directional and fluctuating asymmetry across populations with diverse subsistence strategies. With regard to the interpretation of Fig. S19 (lines 627-629), it's unclear how the variation reflects differences in canal thickness rather than shape. The arrow directionality (i.e. left-to-right ears) along the morphospace appears rather stochastic rather than unidirectional, i.e. in Fig. S19, the canal thickness seems to be larger along the positive ends of PC1 and PC2, but the arrow directionality is not uniformly toward that plot quadrant.

4. Methods, landmark pre- and post-processing: The authors should clarify the steps associated with landmark processing. The added content in lines 544-551 is confusing because it appears that the B-spline was applied in the shapes package, which I don't think is the case (and is certainly not reflected in the R code included). Briefly clarify whether the equidistant algorithm was applied in Avizo, for example, or include the proper R package in the main text and R code in the supplementary information.

5. Methods, intraobserver measurement error test: the new analyses reported in Fig. S17 are good additions, but the authors should note still that a better representation would be one that takes the PCA results of the full dataset and then projects the repeated measures onto that space. The objective for this kind of visualization is that it informs whether variation in measurement error is greater than variation between individuals along specified axes of variation (i.e. whether non-replicates plot within variation of replicates of a single individual). For the Euclidean distance reported in Fig. S17, justify why the first 5 PCs (and not more or less) were used. Lastly, note additional ways of accounting for measurement error that would not necessarily be limited to certain PCs (see again, e.g. Ward et al. 2022).

Minor points
6. Include "Ref." prior to stand-alone citations (e.g. Line 516, line 518)

7. Figure S19: the black convex hull (Nile Valley Late Paleolithic) is difficult to follow with the black arrows: change the color to enhance visualization

8. Double check the correspondence of the enumerated references in the main text and the bibliography list since several do not match up.

9. The sample group names are still difficult to follow, particularly between the main text and the supplementary information. For example, "Middle Nile Valey" has now been adopted in most parts of the main text but not so in the figures of the main text, nor in several parts of the supplementary files. I recommend establishing names (and possibly abbreviations) in Table 1 and using that as a template to check consistency on all other references to the groups throughout the main text and supplements.

Reviewer #2

(Remarks to the Author)
I co-reviewed this manuscript with one of the reviewers who provided the listed reports. This is part of the Nature Communications initiative to facilitate training in peer review and to provide appropriate recognition for Early Career Researchers who co-review manuscripts.

Reviewer #3

(Remarks to the Author)
I appreciate the substantial effort the authors have made in revising the manuscript. Several of the points raised in my previous review have been addressed in a serious and thoughtful manner. In particular, the improved framing of the study within the existing dental and archaeological literature, the clearer description of methodological procedures, and the more cautious and precise language used when discussing PCA patterns have all significantly strengthened the manuscript. Overall, these changes have improved both the clarity and the rigor of the work.
That said, there are still a few aspects of the revised version that I find less convincing and that, in my view, would benefit from further refinement:
1. The interpretation of individuals described as having "Neandertal-like" bony labyrinth morphologies, together with the discussion of associated pathologies, remains stronger than the available evidence fully supports, despite the added acknowledgment of limitations. I would encourage the authors to further separate the descriptive results from the explanatory scenarios proposed (e.g., isolation, endogamy, fixation of mutations), and to frame these scenarios more explicitly as alternative hypotheses rather than as preferred or implied explanations.
2. While I understand the authors' rationale for retaining and interpreting PC2 in the high-dimensional analyses, its prominent role in the Discussion would benefit from a clearer distinction between statistically supported structure and heuristic or exploratory visualization. Making this distinction more explicit would help avoid over-weighting patterns that are primarily exploratory in nature.
3. The additional supplementary material on curatorial histories and data access is a welcome improvement. However, I believe the manuscript would be further strengthened by including a brief reflective statement in the main text that acknowledges broader structural issues related to data access, representation, and authorship in African research contexts.
4. An additional point that became clearer to me after reading the revised manuscript alongside the other reviewers' reports concerns the geographic scope of the conclusions. Although the authors appropriately note sampling limitations, some parts of the main text and conclusions still read as broadly representative of "northeastern Africa," despite substantial gaps in regional coverage—most notably the Lower Nile Valley and Holocene Egypt. I would recommend tightening the language throughout the main text so that the scale of inference more consistently reflects the actual spatial and temporal distribution of the samples, rather than relying primarily on caveats placed in the Supplementary Information.
5. Finally, some interpretive sections of the Discussion still extend beyond what is explicitly demonstrated in the Results, particularly when linking morphological patterns to climatic or demographic processes outside the Nile Valley. While these broader interpretations are intellectually stimulating, the manuscript would benefit from more frequent signposting back to the specific results on which each inference is based, in order to maintain a clear distinction between empirical findings and broader synthesis.

Minor points:
• Line 93: "The Middle Nile Valley (i.e., the area extending from the First to the Sixth cataract of the Nile, in southern Egypt and Sudan) is one of the few regions where large skeletal assemblages exist." One of the few regions in Africa? Please clarify.
• Line 105: Please briefly explain why "aDNA remains very limited."
• Lines 136–138: "to interpret patterns observed elsewhere" is too vague; please clarify the intended meaning.
• Line 155: I suggest revising to "focus on describing the results of the DSM analyses, with more detailed information on the 2D and landmark-based analyses…" rather than "focus on the DSM analyses, with details on the 2D and landmark-based analyses…".
• Line 435: It is not clear what is meant by "demographic conditions."
• Lines 464–466: Please specify how many individuals had paired bony labyrinths analyzed.
• Line 500: I suggest revising to "Given the differential state of preservation…".
• Lines 523–531: Please add citations supporting the claim of "the largest comparative dataset possible of previously published inner ear measurements."
• Lines 651–652 and 658–659: The same information appears to be repeated; please consolidate.

I would like to emphasize that my remaining concerns do not call for additional analyses or new data. Rather, they relate to further refinement of the interpretive framework and tone, particularly where exploratory patterns are linked to specific evolutionary or demographic processes. These comments are offered in a constructive spirit: I consider the dataset and analytical work to be of high quality, and I believe that a more explicitly cautious and clearly signposted interpretive stance would ultimately strengthen both the impact and the credibility of the paper.

Reviewer #4

(Remarks to the Author)
This paper considers morphological approaches to investigating population history and variability in northeast Africa based on bony labyrinth morphology, thereby complementing aDNA and other morphometric analyses (dental morphology). Bony labyrinth morphology not only offers new possibilities of investigation in situations where the two other approaches are not fully available, but also—where the data allows—demonstrates the enormous value of using all three morphological

systems in combination.
The study involves 148 human individuals mainly from the Middle Nile Valley and a small number of individuals from a wide range of other geographical locations, including the Lower Nile Valley, Horn of Africa, European, South and central Africa. The chronological scope covers a wide time span between 44 to 3 ka, i.e. from the late Pleistocene to late Holocene periods, thus involving both forager and food-producing groups. The results show a significant degree of morphological discontinuity between some forager and food-producing groups as well as some evidence for continuity and admixture in other groups in this region.
The article is very thoroughly researched and substantiated by past and current research results. The data and results are comprehensive, well-presented and have great significance for understanding potential population dynamics in the region of the Middle Nile Valley. The wide chronological scope may have implications for understanding the possible contribution of human migration and ancestral variability of social groups in the process of the Neolithic transition in this part of northeastern Africa.

Version 2:

Reviewer comments:

Reviewer #1

(Remarks to the Author)
The revisions have sufficiently addressed the methodological and theoretical concerns from previous versions of the manuscript, adding necessary analyses and framing limitations of interpretation from the results provided. The dataset, in particular, will be an important contribution to the field. Some editorial and copy-editing changes might still be necessary.

Reviewer #2

(Remarks to the Author)
I co-reviewed this manuscript with one of the reviewers who provided the listed reports. This is part of the Nature Communications initiative to facilitate training in peer review and to provide appropriate recognition for Early Career Researchers who co-review manuscripts.

Reviewer #3

(Remarks to the Author)
I would like to acknowledge the effort invested by the authors in revising this manuscript. The current version is clearly improved in terms of clarity, methodological detail, and overall framing. Overall, the manuscript is now much more robust. The remaining points mainly concern clarifying a few methodological aspects and tightening the link between the results and their interpretation. Addressing them would further strengthen the paper and make the argument even more solid.
Major points
1. Identification of fistulas and distinction from segmentation artifacts: I would appreciate some clarification on how fistulas were distinguished from potential segmentation artifacts. Given the variability in preservation and scan resolution, it would be helpful to specify the criteria used to identify true anatomical features versus artefactual discontinuities (e.g., consistency across slices, bilateral occurrence, comparison with anatomical landmarks, or validation by multiple observers). Since these features play an important role in the interpretation, a more explicit explanation would strengthen this part of the manuscript.
2. Diagnostic criteria for inner ear pathologies: It would be useful to clarify how inner ear pathologies (e.g., enlarged vestibular aqueduct, cochlear nerve canal stenosis, semicircular canal hypoplasia) were identified. In particular, please indicate whether these diagnoses are based on relative comparisons within the sample or established clinical criteria, and how comparability was ensured across specimens.
3. Scan resolution and comparability: Given the relatively wide range of voxel sizes used in the µCT scans, a brief discussion of how differences in resolution might affect the identification of small anatomical features—especially pathological traits—would be helpful.
4. Segmentation and measurement procedures: Please clarify who performed the segmentations and who carried out the measurements. If more than one operator was involved, it would be useful to indicate whether inter-observer consistency was assessed, or whether all procedures were conducted by a single analyst.
5. Interpretation of pathological patterns: The discussion of inner ear pathologies has improved, but I would still encourage keeping a clear distinction between descriptive findings and broader demographic interpretations. Where multiple explanations are possible (e.g., isolation, endogamy, developmental processes), it would be good to continue presenting them as alternative, equally plausible scenarios.
Minor Comments
Line 136: Please revise to: "is also detectable when studying the bony labyrinth."
Line 139: Please revise to: "to interpret patterns of biological affinities in these regions."
Line 164: Please revise to: "This mainly relates to a combination of characteristic morphological traits observed in Neandertals."
Line 421: Please clarify what is meant by "cases" (e.g., individuals, specimens, or pathological instances).
Terminology: Terms such as "affinities," "similarities," and "continuity" are used throughout the manuscript and sometimes seem interchangeable. It may help to ensure consistent usage or briefly clarify how these terms are being used in this context.

Open Access *This Peer Review File is licensed under a Creative Commons Attribution 4.0 International License, which permits use, sharing, adaptation, distribution and reproduction in any medium or format, as long as you give appropriate credit to the original author(s) and the source, provide a link to the Creative Commons license, and indicate if changes were made.*
In cases where reviewers are anonymous, credit should be given to 'Anonymous Referee' and the source.
The images or other third party material in this Peer Review File are included in the article's Creative Commons license, unless indicated otherwise in a credit line to the material. If material is not included in the article's Creative Commons license and your intended use is not permitted by statutory regulation or exceeds the permitted use, you will need to obtain permission directly from the copyright holder.
To view a copy of this license, visit https://creativecommons.org/licenses/by/4.0/

REVIEWER COMMENTS

Reviewer #1 (Remarks to the Author):

This study explores the morphological variation of bony labyrinth morphology in African and western Eurasian human populations spanning the last circa quarter million years. Most original data derive spatially from across the Nile Valley and temporally from contexts spanning the Holocene and terminal Pleistocene. The manuscript is difficult to follow because no clear hypotheses are provided at the outset, the aims are rather broad, and the discussion provides interpretations that go beyond what the data can properly address. While the Introduction provides the general motivation for conducting the exploratory study, the first two of the three aims are not sufficiently justified or addressed with the analyses undertaken. In addition, there are some study design and methodological choices that need to be addressed before the manuscript can be considered for publication. Nevertheless, the manuscript excels in that (1) the original data is extremely valuable given its relative scarcity, (2) it applies three analytical methods (2D morphometrics, 3D-GMM, and DSM) to one anatomical element, and (3) provides careful analysis of pathological features, likely often overlooked in archaeological/paleontological studies of the bony labyrinth. I expect several revisions will be necessary to bring this manuscript to a publication stage that is commensurate with this journal and accessible to its wide readership.

The points below outline some of the major and minor areas of improvement in revising the manuscript.

Major points:

1. Study Design, justification: the primary reason the manuscript is difficult to follow is that it is attempting to address several topics at once with variable success relative to the data available, including: (1) human population structure in Africa since the Late Pleistocene, (2) population (dis)continuity, (3) comparison of inferences on population (dis)continuity in the Nile Valley from the original bony labyrinth results to previous dental studies, (4) morphological bony labyrinth differences between foragers and food producers, and (5) the effect of pathologies on bony labyrinth form. The Introduction and Discussion further introduce (6) climate variability as a corollary. The aims that are actually articulated (lines 123-127) are roughly aligned with Topics 3-4. Topics 1-2 likely stem from questions that were set out when the petrosae were sampled for aDNA, but more intractable with the samples examined here (and including the comparative data) and the methods employed. Topics 3-5 are more directly tied to the original samples and would benefit from more information on their archaeological context. Limiting the paper to topics 3-4 would make for a much stronger manuscript. Overall, because none of the topics is tied to explicit hypotheses in the Introduction, the entire manuscript is difficult to follow and reads as too ambitious and speculative despite some very interesting analyses.

We thank the reviewer for this comment. We agree that in the submitted version, the breadth of the topics addressed was not articulated with sufficient clarity, which made the integration of the different components of the study less explicit than intended. Importantly, our aim is not to address

these topics independently, but to show how they are interdependent and must be considered together in order to make sense of the population history of northeastern Africa.

Specifically, interpretations of morphological variation (topics 3–4) depend on the broader demographic context (topics 1–2), while the assessment of bony labyrinth morphology requires attention to potential pathological effects (topic 5) and to the climatic dynamics that structured mobility and settlement in the region (topic 6). Removing any of these components would artificially compartmentalize evidence that must be considered holistically.

To clarify this interdependence and complexity, we have substantially revised the Introduction, which now clearly articulates how population structure, continuity/discontinuity, and climate variability converge into a single overarching research question. The same way, it now presents a unified set of integrated research objectives and hypotheses aligned with these themes.

Additionally, we would like to address the reviewer's suggestion that topics 1–2 may stem from questions originally formulated when the petrous bones were sampled for aDNA. For clarity, the selection of the petrous bones that are presented in this study was not a collateral outcome of aDNA sampling efforts. Rather, the individuals included in this study were specifically and independently selected for the morphological analyses presented here, with the goal of documenting variation in the bony labyrinth to address the population/archeological questions articulated in the manuscript. If aDNA analyses were conducted on some of the samples included in this study, such efforts were conducted *after* primary morphological assessment (e.g., for Ishango, Hofmeyr, or Nazlet Khater). The only exception is Shum Laka, were both studies were conducted in parallel, for *some* individuals only. Our sampling strategy was therefore explicitly designed for this study's aims and does not derive from an aDNA-driven collection process.

We appreciate the reviewer's thoughtful critique, and we hope that the revised *Introduction* and clarifications demonstrate more clearly the coherence and focus of the study.

2. Study Design, aim 2 and comparative samples: while it could be useful to include a recent South African sample and Neanderthal samples in a comparative context, there isn't a clear justification for doing so. For example, how is a recent South African population relevant here? In other words, why is it useful to compare recent South Africans to Late Pleistocene-Holocene populations from Northeastern Africa? In the same vein, why should Neanderthals be included? Is there some relevance, for example, to the back-to-Africa Neanderthal discussion from population genetics or Nubian technology discussions from archaeology? Or is it simply to contrast the Neanderthal-specific bony labyrinth morphology? Similarly, how are samples from Cameroon relevant here? Justifying the inclusion of the samples is important because it will affect the results. For example, calculation of momenta in the DSM analysis might be strongly biased with the recent South African sample. For specialists in the field, this manuscript reads largely as an opportunistic exploratory study that takes the scans of petrosae that were sampled for aDNA to simply put into comparative context with other data available, then work a story around it. That in itself is absolutely fine, but does not make for a clear hypothesis-driven paper commensurate with the quality of publications in this journal. Perhaps there were indeed hypotheses at the outset, in which case they should be clearly articulated (see comment 1, above). What might make the most sense is to focus on aim 3 and limit the analyses spatially to the Nile Valley and Horn of Africa and temporally to the terminal Pleistocene and Holocene. Other samples could be part of a separate manuscript with additional data to property address aims 1-2.

As mentioned in the answer to the reviewer's previous comment, the inclusion of all comparative samples in this study was hypothesis-driven and is central to interpreting the morphological variation in the Nile Valley and broader northeastern Africa, rather than opportunistic. Specifically:

- Recent South African individuals were selected to provide a modern reference point for intraspecific morphological diversity in anatomically modern humans and in a given population. Including this sample allows us to contextualize variation observed in Late Pleistocene–Holocene northeastern African populations and helps identify which morphological differences are population-specific versus reflecting broader *Homo sapiens* variation.

- Neandertals were included as a well-characterized outgroup for the bony labyrinth. Their inclusion allows us to identify human-specific morphological traits, provide deeper evolutionary insights and, importantly, polarize variation within *Homo sapiens*. For the latter, it also allowed for a better assessment of the few "outlier" *Homo sapiens* individuals, who would have otherwise been artificially forced towards the rest of the sample (n= 115) in the computation of the PCA morphospaces. In this regard, our primary rationale is functional and phylogenetic contrast, not opportunistic inclusion.

- Cameroonian and Horn of Africa comparative samples: these specimens allow us to explore regional morphological affinities across northeastern Africa and contextualize Nile Valley variation within a broader continental framework. The inclusion of Central African and Horn of Africa specimens is crucial to evaluate hypotheses about population dynamics and divergence within northeastern and central Africa. Moreover, they provide rare data for under-documented regions and time periods, thereby enabling more robust and meaningful interpretations at the continental scale.

Importantly, all samples were selected specifically to address the research questions of this study, not as secondary outcomes of aDNA sampling. Analyses involving these comparative samples are framed in the context of testing explicit hypotheses about population continuity, regional affinities, and morphological variation (see Introduction). While the core focus of the manuscript remains on the Nile Valley and Horn of Africa during the Late Pleistocene–Holocene, comparative samples are essential for interpretation. We have clarified these points in the revised Materials section.

Finally, regarding the reviewer's concern about potential bias in the computation of momenta in the DSM analysis, we agree that sample composition can influence the global mean shape used in the model. However, the momenta themselves describe individual-specific deviations from this mean and are robust to changes in sample composition. While different choices of comparative samples may slightly affect the estimated global mean shape, such effects are not expected to meaningfully alter the resulting momenta or their interpretation, and will instead rather have an influence on the computation time.

3. Study Design, use of bilateral labyrinths: the use of two labyrinths for one individual (i.e. the right and left inner ears) in the analyses in only the cases where they are available raises some important implications that must be further explored to ensure robust interpretation of the results. Unless otherwise unspecified, each labyrinth is essentially being treated as one case in all analyses, even

when two bilateral labyrinths are from the same individual case/specimen. This will produce a bias in terms of appropriately quantifying variation of groups because some individuals are considered twice and others only once when bilateral labyrinth data is unavailable. More concerning is that a brief inspection of the 2D measurement values and the PCA distribution shows that the differences within individuals might be significant. For example, the Supplementary Data S2 corresponding to Fig. 2A in the main text can be used to explore the fact that in some cases, e.g. PBC_5089, the L and R plot relatively distant from each other, with several other cases in between. This implies that the variation within individuals can be greater than variation between individuals and populations: note, for example that PBC_5089R plots within the convex hulls of two other groups, while PBC_5089L does not. One solution is to simply take the average values of the L and R for each case so as to not bias the variation within and between groups, which is the primary aim of analysis in this study. It would then also be necessary to reassess whether the remaining sample sizes of true cases is sufficient for the statistical analyses employed, with justification of parametric and non-parametric methods. At the same time, it could be interesting to look at the bilateral data separately and assess, for example, whether the degree of asymmetry is larger in the outlier cases (see also comment 7 on pathologies, below). Again, a careful evaluation of sample sizes is necessary here depending on the statistics employed.

We thank the reviewer for this thoughtful and detailed comment. We agree that the use of bilateral labyrinths can raise important questions regarding independence and the quantification of within- and between-individual variation. In our dataset, both labyrinths were included whenever available. This applies to all present-day South African individuals and to several archaeological specimens. We chose this approach for two main reasons.

First, including both antimeres allows us to document biologically meaningful asymmetry in bony labyrinth morphology. As noted by the reviewer, some individuals exhibit substantial left-right differences, in some cases comparable to or exceeding between-individual variation. We interpret this intra-individual variation as a genuine morphological feature rather than measurement noise. Excluding one side would therefore remove information that is relevant to understanding the full range of variation present in the dataset. To make the extent of bilateral differences explicit, we now provide a supplementary PCA figure in which left and right labyrinths from the same individual are connected by lines. This visualization allows readers to directly assess the magnitude of within-individual variation relative to between-individual variation and to identify cases of marked unilateral deviation. Notably, most left-right pairs show vectors oriented in a similar direction in morphospace, generally from lower to higher PC1-PC2 values. This consistent trend suggests that bilateral differences are not random but are largely associated with variation along the shape axis corresponding to increased semicircular canal thickness.

We also acknowledge the reviewer’s concern regarding the lack of statistical independence when left and right labyrinths are treated as separate observations. Because the primary analytical steps in this study (PCA and qualitative comparisons of morphospace structure) are descriptive and exploratory, they do not rely on parametric assumptions of independence in the same way as inferential hypothesis-testing procedures.

Second, the identification of individuals with atypical or pathological labyrinth morphologies—one of the key aims of this study—required bilateral data. In one “outlier” case (a specimen from the PBC series), the unusual morphology is unilateral. Retaining both sides was therefore necessary to detect such individual; restricting analyses to a single side could have led to the omission of such cases.

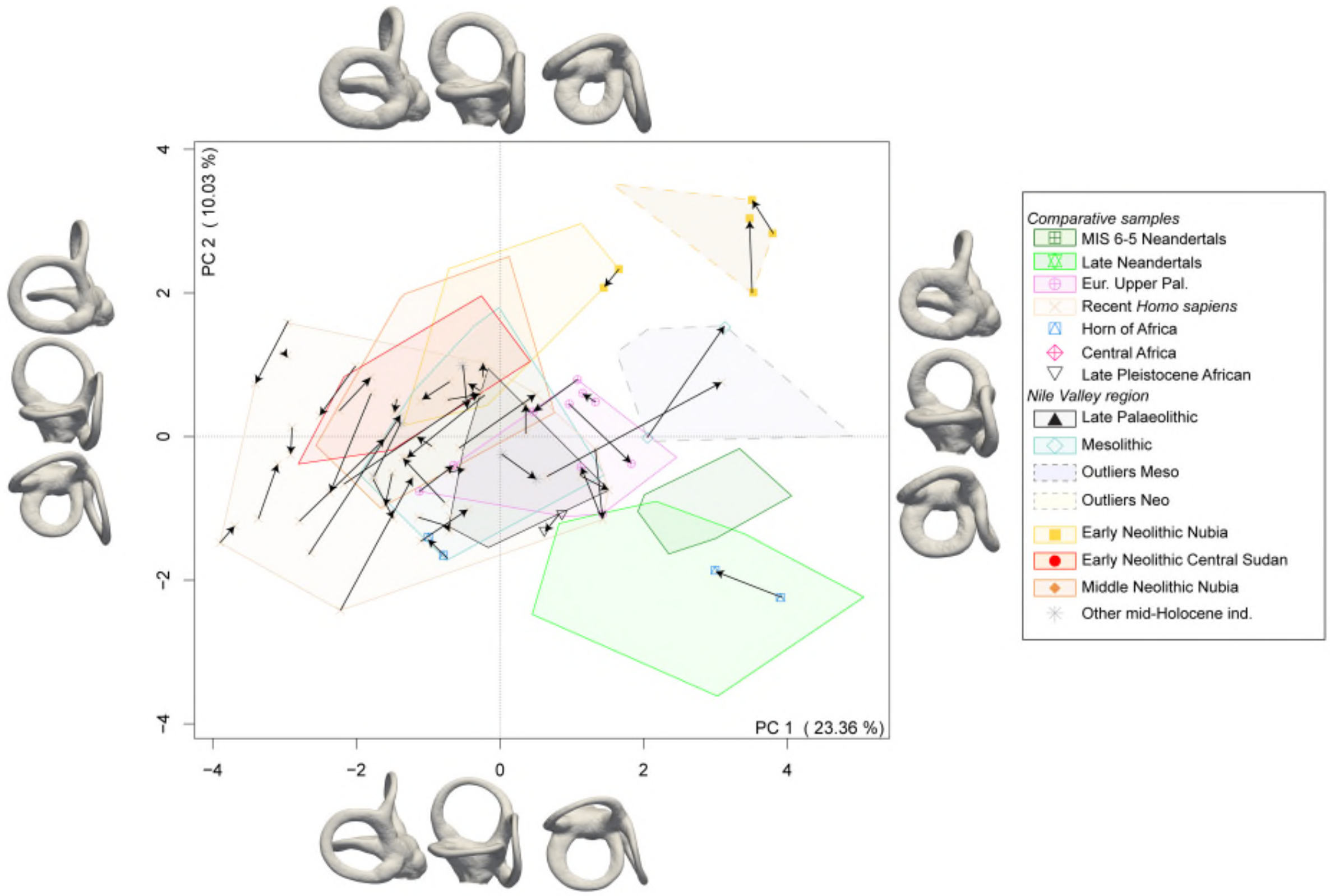

New SI fig highlighting the asymmetry in our study sample. Each pair of individual is connected by a black arrow (L→R)

With respect to the suggestion of averaging left and right sides, we agree that this approach is feasible for the 2D linear measurements. However, averaging 3D landmark configurations is not a straightforward arithmetic operation: it requires explicit symmetry procedures that remove unilateral information and can distort the underlying shape geometry. Such averaging would also mask the very unilateral morphologies that are central to this study. For these reasons, we retained left and right labyrinths as separate observations in the 3D analyses.

We added a mention of these methodological considerations to the main text and included the new supplementary figure illustrating within-individual asymmetry.

4. Study Design, group composition and sample sizes: It is difficult to follow how groups were delimited and what the sample sizes were in each analysis. For example, Table 1 lists 10 sample groups (5 original, 5 comparative), while Figure 1 implies having 12 groups (7 original, 4 comparative, and 1 overlapping), and Figure 2 implies having 13+ groups (6 original, 6 comparative + 2 for 2D analyses, 1 overlapping, plus 2 outlier groups), etc. At a minimum, Table 1 should reflect the groupings most commonly used in the analyses so that sample sizes can be assessed efficiently by the reader (see also comment 3, above, on case duplicates). The Nile Valley groups might be well sampled, but some assessment of this should be presented. The Horn of Africa and Central Africa samples are more problematic. For the Horn of Africa group, the single Gogoshiis Qabe case is spatially and temporally more distant to the others, so some justification of inclusion is necessary. For Central Africa, it is not clear why the samples should be treated as one group when the temporal span is almost as large as

that of the Holocene Nile Valley samples, which comprise 6 groups. Underlying these decisions is both a statistical question in data analysis as well as an evolutionary question on population [dis]continuity, the latter of which forms a large part of the discussion and interpretation of the manuscript.

To facilitate the identification of the sample composition, we homogenized the group identification through both figures and Table 1.

Firstly, regarding the Nile Valley groups, this sample includes the largest number of individuals in the study and spans multiple sites, regions, and chronological phases. The reviewer refers to the need for an "assessment" of sample size, which can encompass different methodological considerations. One aspect concerns the potential effects of group size imbalance in between-group PCA. Following the recommendations of Courtenay (2023), we formally evaluated the risk of spurious separation due to unequal group sizes; the resulting balance index of 0.985 indicates very good balance among groups. In addition, as already described in the manuscript, we followed Cardini & Polly (2020) by computing cross-validated bgPCA. The cross-validated results closely matched those obtained in the original analyses, indicating that the observed patterns are not driven by sample size imbalance. Beyond these methodological tests, sample size adequacy was also evaluated descriptively by considering the number of individuals relative to other regions in the dataset and their distribution across multiple sites and time periods. Taken together, these considerations indicate that the Nile Valley groups are adequately sampled for the exploratory and comparative analyses conducted here, within the constraints of available material.

Regarding the inclusion of "spatially and temporally distant" individuals in the Horn of Africa and Central Africa samples: we believe these inclusions are essential to the study's objectives. Their geographic and chronological distribution provides a unique opportunity to evaluate morphological trends over broader temporal and spatial scales. In particular, including the Horn of Africa specimen (Gogoshiis Qabe) and temporally dispersed Central African individuals allows us to anchor the observed morphological variation within a deeper chronological context and a broader regional framework, which is critical for interpreting population structure and [dis]continuity across northeastern and central Africa.

Importantly, in all analyses involving these individuals, we do not treat these assemblages as statistical "groups". Instead, we use methods such as PCA, which allow us to explore patterns of homogeneity or heterogeneity within these assemblages. In these PCAs, we also specifically avoided using convex hulls for these Horn of Africa and Central African assemblages, to avoid misleading the reader. The sole exception is the disparity analysis (Fig. 3, now Fig. 4), which was specifically designed to test whether these "groups" are morphologically homogeneous. This approach minimizes bias from the wide temporal and spatial coverage, while allowing us to address a central aim of the study: evaluating whether populations are morphologically cohesive or structured across space and time.

5. Discussion, PCA results: In describing PCA results, avoid terminology that suggests the analysis is associated to, or is itself, a discriminant or classificatory analysis or that there are clear boundaries (e.g. line 133: "distinguishes," line 134: "separation," line 138: "differences," Supplementary Note S1: "clear distinction", etc.). First, it is clear that there is substantial overlap between the different groups and, to a lesser degree, between taxa, so it would be more appropriate to refer to general patterns in PCA space. Even so, the Discussion might still be difficult to follow with reference to the figures. To better appreciate the trends, one possible form of displaying the PCA result is to delineate the convex hulls and only use the symbol for the mean of each group in the main text's Fig. 2, referring to Supplementary Data S2 for further details. Secondly, terminology that suggests discrimination between

groups in PCA results is particularly confusing because bgPCA and PERMANOVA are also employed. Thirdly, since much of the discussion relies on patterns and inferences from the PCA, identifying whether the sample sizes are sufficient to do so should be explored (e.g. getMeaningfulPCs() function in Morpho; see Bookstein 2014, Measuring and reasoning: numerical inference in the sciences. Cambridge University Press).

We thank the reviewer for these suggestions. In response, we have rephrased or removed terminology in the manuscript and supplementary material that could imply classification or group discrimination (e.g., “distinguishes,” “separation,” “clear distinction”). Figure 2 (now Fig. 3) has been updated to show group convex hulls with symbols representing group means, as suggested.

Following the reviewer’s recommendation, we applied the *getMeaningfulPCs()* function from the Morpho package (following Bookstein, 2014 suggestion) to evaluate whether the PCs in our high-dimensional DSM dataset (2,800 variables, n=198) are statistically meaningful. As expected in high-dimensional morphometrics, only PC1 exceeds the eigenvalue “noise floor”. This is a well-documented consequence of high-dimensional datasets, where the large number of variables elevates the “noise floor,” making secondary axes less likely to pass strict statistical thresholds.

However, we have chosen to retain the discussion of PC2 for two key reasons:

- Biological coherence: As noted and extensively explained in the text, the shape changes associated with PC2 are not random noise; they correspond to specific, biologically coherent morphological traits that are clearly observable in the specimens themselves. Specifically, negative PC2 values are associated with wider lateral and posterior semicircular canals and a more angular posterior canal, while positive values reflect rounder and straighter posterior canals and narrower lateral canals (see Main Text Fig. 3 and Results section).

- Descriptive value: We utilize PCA here primarily as an ordination technique to explore the morphospace structure. While PC2 may not pass the strict threshold for latent variable inference under Bookstein’s criteria, it remains the most effective vector for visualizing this specific secondary axis of variation which, as we discuss, captures trends related to geography, chronocultural context, and taxonomic differentiation (e.g., Neandertal vs. *H. sapiens* outliers). Removing PC2 would obscure these biologically relevant patterns, making its descriptive value critical for interpreting the dataset.

- Consistency across analyses: The same morphological variation patterns captured by PC2 in the DSM dataset are evident in the landmark-based and 2D analyses (see Main Text Fig. 3, Results section, and SI Note S1). However, for these datasets, *getMeaningfulPCs()* identifies the first 2–4 PCs as meaningful, reinforcing that PC2 in the DSM approach reflects true, robust morphological variation rather than statistical artifact.

We have added a sentence to the Methods section explicitly clarifying that while PC1 is the dominant statistical signal, PC2 is interpreted heuristically based on its biological content.

6. Discussion, differential preservation of population history: The argument that the bony labyrinth is more conserved than teeth (lines 213-226) appears to be based on relative measures of disparity, but it is not clear from this Discussion whether the different dental and bony labyrinth datasets can be properly compared. Particularly since this is reiterated as a conclusion of the paper, more work is

necessary to substantiate the claim. For example, were the same individuals and populations sampled in previous dental studies also sampled in this study? Were the analytical methods similar?

We thank the reviewer for this comment and agree that additional clarification was needed to properly contextualize this comparison. The comparison between bony labyrinth and dental morphology was made with previously published analyses of enamel–dentine junction (EDJ) morphology from the Nile Valley (Martin et al., 2025), which sampled individuals from the same archaeological sites included in the present study (Jebel Sahaba, El-Barga, Kadruka, Sphinx, Fox Hill, and Ghaba). Importantly, a subset of individuals is common to both datasets. Both structures were analyzed using similar approaches (namely DSM and landmark-based analyses), ensuring comparable results.

To clarify this point, we now include a new **Supplementary Figure (Fig. S12**) presenting comparative analyses, clearly identifying individuals shared between the dental and bony labyrinth datasets. This allows a direct, individual-level comparison of morphological variation across the two anatomical systems.

In addition, to further support the observation that bony labyrinth morphology exhibits reduced inter-population variation relative to EDJ morphology, we now provide an additional statistical comparison of classification accuracy between both datasets (described in **Supplementary Note S3**). This analysis confirms that, while both structures capture similar population-level patterns, the magnitude of morphological differentiation is lower in the bony labyrinth.

We have revised the Discussion accordingly to clarify that this conclusion is based on directly comparable samples and complementary analytical approaches.

7. Discussion, congenital pathologies and climate: this section (lines 290-373) is a substantial portion of the discussion and, while indeed stimulating, requires more context. For example, some indication of the archaeological background and taphonomic state of each sample should be included (e.g. as supplementary information) in order to rule out the possibility that some of these features are due to taphonomic effects (particularly for the assessment of EVA). The taphonomic state is also important for understanding possible variation in calculating the HMH for segmentation and the resulting values derived for the subsequent 2D, 3D-GMM, and DSM analyses (see also comment 10, below). The sociocultural interpretations of the archaeological record are also important because endogamy is assumed here to be the result of changing climate conditions (line 347) but could also be associated with behavioral (social, cultural, economic) practices, or both. The general impression is that the section on pathologies could be another paper altogether and better when more fully explored.

We agree that additional contextual and taphonomic information was needed for each sample. To address this, we created a new Supplementary Table (S5) summarizing, for all original African samples, their overall preservation, sediment/matrix infilling, diagenetic alterations, and the clarity of the anatomical structures examined. To more directly address the reviewer's concern regarding the assessment of pathological features in relation to taphonomy, we also added a new Supplementary Figure (S19) illustrating the range of preservation states observed and how these affect the visibility of relevant structures.

In addition, we revised the Methods section to more clearly describe the recording procedure, explicitly stating that taphonomic state was evaluated prior to scoring. When a pathological feature could not be observed or measured with sufficient confidence due to preservation issues, the individual

was recorded as “non-observable.” This situation occurred only rarely in our sample and affected primarily the measurement of the vestibular aqueduct width at the operculum level.

Regarding the sociocultural interpretations raised by the reviewer, we fully acknowledge that behavioral, social, or cultural practices can structure mating patterns and potentially lead to endogamy. However, based on the data presented in this study, we do not consider such behaviors to be the sole nor primary explanation for the observed distribution of congenital pathologies in the samples analyzed. The conditions we report occur in individuals separated by more than 30 ka (e.g., Nazlet Khater 2 and Mesolithic Nile Valley groups), which limits the plausibility of a single sustained socio-behavioral mechanism underlying these patterns. Nonetheless, for the Early Holocene/Mesolithic context specifically, archaeological and cultural evidence does suggest particular social behaviors among forager populations, including high levels of interconnection and complex post-marital residence networks (e.g., Brukner Havelková et al., 2023; Osypiński et al. 2022; Salvatori & Usai, 2019). This point was already hinted at in the main text when referring to populations that were “interconnected at the regional level during the early Holocene.” To improve clarity, we have now rephrased this section of the discussion to articulate more explicitly the potential role—and the limitations—of sociocultural factors in shaping the observed biological patterns.

8. Methods, 2D linear comparative data: the original data presented in Table S1 appears to be reported with values to about the 13-14th decimal. This should be consistent in order to avoid subtle differences in the analyses that are simply due to rounding. More importantly, since the previously published measurements are unlikely to be to the 13-14th decimal, the original data should be rounded to the lowest common decimal point in order to avoid any inadvertent false interpretation due to rounding. For transparency, all specimens, including those derived from published data, should be compiled in Table S1. In addition, since the study is likely using PCA on both the variance-covariance matrix (in the case of 3D-GMM and DSM data) as well as the correlation matrix (in the case of 2D data), this should be confirmed and stated.

In response to the reviewer’s comment, all original 2D linear measurements were rounded to the first decimal to ensure consistency across the dataset and to match the precision of previously published measurements. All PCA computations and subsequent analyses were then recalculated using this homogenized dataset. Although this adjustment had a negligible effect on the results, all relevant figures, tables, and analyses in the manuscript were updated accordingly.

Regarding the inclusion of raw data for all individuals in Supplementary Table S1, due to restrictions related to museum data ownership and ongoing analyses by our team and collaborating researchers, we are currently unable to release the raw measurements for certain specimens. These data can nonetheless be obtained upon reasonable request from the corresponding authors. We have clarified this in the Data Availability statement.

Finally, we clarified in the Methods section whether the PCAs computed in this study were computed on the covariance or correlation matrixes.

9. Methods, landmark post-processing: landmark data pre-processing is described simply as “Procrustes superimposition.” Since the shapes package provides the option of both GPA and OPA, specify for a non-specialist audience that GPA was applied. More importantly, specify whether the semilandmarks were slid or resampled in any way and justify why or why not given the different approaches in the literature and in the cited works. Note that this affects how landmarks are referred

to in line 461 as “pre-defined” points, which would only be true in the sense of landmark correspondence, i.e. pre-defining the number of landmarks defining a homologous region.

We clarified the use of GPA in the Methods section. We also expanded the description of semilandmark processing.

In our revised text, we clarify that semilandmarks were not slid, but were instead resampled at equal arc-length spacing along the B-spline curves with fixed start and end points. Because all specimens preserve continuous and complete canal and cochlear morphologies, and because no thin-plate spline deformation grids were used in subsequent analyses, sliding was not required to reduce warping artefacts. Gunz et al. (2012) note that for uninterrupted curves, differences between slid and unslid equidistant points are typically negligible. We therefore opted for resampling rather than sliding, which ensured consistent point ordering and curve representation without affecting biological interpretation.

Finally, in response to the reviewer’s note on the phrasing “pre-defined points,” we now clarify that “pre-defined” refers to the pre-defined number and ordering of points along homologous curves, rather than implying anatomical homology of each semilandmark in the strict sense. We revised the text accordingly to avoid ambiguity.

10. Methods, intraobserver measurement error test: the measurement error employed is insufficient. In general, a Procrustes ANOVA would be preferable to understand whether the variation observed between repeated measures exceeds the variation between groups under investigation. In addition, the interobserver PCA presented here is helpful only to get an idea about the possible impact of measurement error, but a better representation would be one that takes the PCA results of the full dataset and then projects the repeated measures onto that space. Still, note that this would only present a portion of total variation (e.g. along two axes in a 2D PCA plot). Related to the comment above, it might make sense to limit the error test to the Type 1-3 landmarks rather than at the inclusion of Type 4-6 landmarks (sensu Weber & Bookstein 2011), depending on how the Type 4-6 (semi)landmarks were processed. In addition, specify whether a measurement error was undertaken for calculating the HMH and undertaking the segmentation procedure. This is a difficult and extremely time-consuming task but is important because it directly affects all derived data values. Images of the segmented labyrinths in the supplement suggest that there is substantial variation in the degree of sediment found inside (i.e. taphonomy) and the degree of manual segmentation. If no error test was conducted to test segmentation variability, then it is important to state that variation is assumed to be negligible and, at minimum, some qualitative assessment would be necessary to support such assumption.

To address the reviewer’s concern about the intra-observer error test conducted on the landmark digitization, we provide, along with the PCA already included in the SI material, a statistical comparison of the inter-individual vs. inter-replicate distances. Given the general concerns raised in various previous publications, especially in reliably digitization landmarks and semi-landmark curves at the cochlea level, we did chose to keep the full landmark configuration for this analyses. This way, the strength and reliability of the method chosen in this study is further confirmed.

Finally, considering the implementation of a segmentation reliability test, N.M. segmented twice again a selection of individuals chose from various taphonomic profiles. We submitted these new sets of segmentation to the deformation based (DSM) analysis, the most sensitive to potential segmentation artifacts. The results, presented in a new Supplementary Information Fig. (S14), reveal no significant segmentation error.

Minor points:

11. Throughout the manuscript, are the terms “hunter-gatherers,” “foragers,” and “hunter-fisher-gatherers” interchangeable? If so, simplify to “foragers” or specify the different designations.

Yes, they all refer to the same groups of individuals. We simplified to “foragers” all along the text.

12. Throughout the manuscript, are surface registration and DSM used interchangeably? Note that some methods referred to as surface registration in the literature differ from DSM (e.g. Roeding et al.’s implementation of Schlaeger’s: https://doi.org/10.1002/ajpa.24642), so it is important to clarify.

Yes, they refer to the same approach. We removed “surface registration” and replace it with “DSM” throughout the text.

13. Line 89: first clause is vague (i.e. what does “central role” and “dynamics” refer to?) Clarify or remove.

We rephrased this part as suggested by the reviewer.

14. Line 92: word choice; consider changing “exploitable” to neutral term or more descriptive word (e.g. endogenous)

Done.

15. Line 92: a citation is needed here or further explanation to which of the samples included in the study were sampled for ancient molecules, alongside the success rate of endogenous molecules

As noted in a previous response, most of the samples included in this study were not tested for ancient DNA. Among the few individuals that were sampled for molecular analyses, all returned negative results, except for the published Shum Laka data (Lipson et al., 2020). To the best of our knowledge, other tests conducted on these specimens have not been published, and we do not have access to their success rates.

To provide context, we have added a reference in the main text to Sirak et al. (2022), which offers a recent synthesis of all available aDNA data for the region and clearly highlights the lack of ancient molecular data for the periods covered in this study.

16. Lines 95-96: this sentence is confusing since the first clause implies that the Middle Nile Valley region is an exception to the scarcity of aDNA described in the previous paragraph, yet the last clause says that no molecular data is available. Rephrase for clarity.

We rephrase this paragraph to clarify this part.

17. Line 95: clarify what Middle Nile Valley refers to and/or indicate in Fig. 1

We added more details on the geographical definition of the Middle Nile region here.

18. Lines 105-107: rephrase for clarity: as written, the gap appears to refer to dental studies; in addition, provide brief description of bony labyrinth for the wide readership of this journal

We rephrased this section.

19. Lines 112-113: clarify what "…studied into much detail" refers to or rephrase

Done.

20. Lines 115: change "propose using" to "use"

Done.

21. Lines 115-116: disambiguate "population complexity" with additional details, citation(s), or by rephrasing

We added citations referring to this population complexity, but also a new paragraph detailing the precise regional context. See also answer to Reviewer #3 comments on this latter topic.

22. Line 116: disambiguate "reference point" or rephrase

We rephrase this sentence in the text.

23. Lines 117-122: edit for conciseness; details of fossils will be in Table 1, so there is no need to name them here, particularly for a general science audience

We edited this section, mentioning only the broad geographical and chronological contexts of the samples included in the study, as suggested by the reviewer.

24. Figure 1C: specify what the bars and dashes indicate (i.e. date ranges reported in Table 1).

We update the caption of Figure 1.

25. Figure 2 description: spell out SCC in the first instance for ease of reading without reference to main text

Done.

26. Figure 2 legend: spell out words when there is space to do so; specify here and throughout manuscript what "MIS" refers to (clarify to a non-specialist readership)

We updated this figure, as well as all derived figure in the SI.

27. Figure 1A and 1B: consider moving the B inset down to the lower left corner in order to show that

you've also used Levantine samples (i.e. Qafzeh/Skhul). Add these and the pre-Neanderthals to the map in 1A.

As suggested by reviewer #3, we removed panel B from Figure 1 to make it a stand-alone figure (new Fig. 2). We added the Levantine and pre-Neandertal samples to both the map and in 1A and timeline in 1B (former 1C).

28. Figure 2A, 2C: it is better to indicate surface and wireframe deformations as number of standard deviations from the mean than at "extremes" along the axes, particularly when the axes are not labeled at their terminus

We present the surface and wireframe deformations as the maximum and minimum values along each principal component axis, and these axes are explicitly labeled in each figure. This approach is standard practice in geometric morphometrics, as it provides a direct and intuitive visualization of the main shape differences. Converting the deformations into standard deviations would not add interpretative value and could reduce readability. For clarity and consistency with previous studies in the field, we have therefore retained the original presentation.

29. Figure 2A, 2C: given the distribution of the data, it would be more interesting to present the surface and wireframe deformations at the "extremes" of the quadrant corners (e.g. 2A: the "extreme corner" of quadrant comprising positive PC1 and negative PC2 to show the shape of the quadrant where the majority of Neanderthals plot; the corner of positive PC1 and positive PC2 where the majority of outliers plot)

While presenting deformations at quadrant corners is an interesting alternative, we opted not to implement this change because it would have added complexity without substantially enhancing the key patterns we aim to highlight. The current depiction along the PC axes clearly communicates the directions of maximum shape variation (also see answer to Reviewer 3 comment #4) and allows the reader to directly compare morphologies at the extremes of the principal components, which is the most relevant information for the purposes of our study. Introducing quadrant-corner extremes could potentially confuse interpretation and reduce the overall readability of the figure.

30. Figure 2B: identify in figure description what the biplot is; move ASC%R label within the biplot to avoid confusion

Done.

31. Figure 2 and Table 1: avoid confusion by using the same labels for the samples

We homogenized the names of the samples.

32. Results & Discussion: avoid using the term "fall" for describing the positioning of cases in PCA space. Use "plot" or other descriptive terminology with respect to eigenvalues/eigenvectors.

We replaced every instance of "fall" within the manuscript with more adapted terminologies/phrasing.

33. Lines 162-163: it is unclear how the disparity values for Horn of Africa and Central Africa samples are comparable to the recent South African and EUP samples. Check if this is correct or clarify.

This was incorrect. We modified this sentence.

34. Line 242: this is the only instance where the term “archaic” is used and, without further context, vague. The sentence could be rephrased and simplified or further contextualized.

We rephrased this sentence to avoid the use of “archaic”.

35. Line 262: disambiguate “opposite affinities.” Do you simply mean that they plot on both negative and positive parts of PCs? It would be better to describe their affinity to other individuals (e.g. ProcD, MahalanobisD, MST, etc.) or groups.

We have clarified that the term “affinities” refers to the relative positions of individuals in PCA morphospace and the associated morphological differences, rather than to distance-based measures.

36. Lines 418-420: explain that these are parameter range values, with specific values dependent on specimen

Done.

37. Lines 424-425: clarify how Avizo and Biomedisa were used in conjunction, as well as what “manual corrections” refers to

We developed this part to clarify the joint use of Avizo and Biomedisa, as the “manual corrections”.

38. Lines 426-427: provide justification for smoothing and padding value

Done.

39. Line 430: change “plan” to “plane”

Done.

40. Lines 430-431: the works cited by Spoor et al. do not use Avizo for defining the transverse and sagittal planes, so be specific about how this was done (e.g. defining the transverse plane relative to the lateral canal?). Also clarify the interpolation step of this function.

We clarified the reorientation process in Avizo. The “*Resample Transformed Image*” function in Avizo was applied using the ‘standard’ interpolation option, which corresponds to trilinear interpolation in the software. This method calculates voxel values in the resampled image as weighted averages of the neighboring voxels, providing a smooth transformation without introducing significant artifacts. We have clarified this in the Methods section to specify the interpolation approach used.

41. Supplementary Data S2: ensure consistency of the group names used in the main text and other

parts of the manuscript. Acronyms used here can be deciphered in most instances but might not be obvious to non-specialists. For example, it might not be clear that HHEID in the 2D PCA plots refers to the Pre-Neanderthals in other parts of the manuscript.

We thank the reviewer for highlighting this issue. We corrected and homogenized the group names in all Supplementary Data S2 .HTML files.

42. Line 437: clarify that "largest dataset possible" refers to maximizing comparative samples from Africa, the Levant, and western Eurasia (e.g. eastern Eurasia is not represented)

Done.

43. Line 441: specify what each study "inspired" (and see major comment 9, above)

We clarified this in the Methods section.

44. Line 461: specify that "pre-defined" refers to the number of landmarks.

Done.

45. Line 464: justify the selection of 30k triangles in the decimation procedure

We included references to the original protocols.

46. Supplementary Note S1: it should be referenced in the main text

Supplementary Note S1 is already referenced multiple times both in the Results and Discussion sections.

47. Supplementary Note S1: Homo sapiens should be italicized consistently (it is not prior to referring to Qafzeh and Skhūl)

We thank the reviewer for point this out. This has been modified.

48. Figure 4: specify in the figure description what the specific pathologies are for C-E.

Done.

49. Lines 504-505: specify how the transverse/axial views were standardized across samples

In this section, we used "transverse/axial" in reference to the transverse views produced for the 2D measurements analysis (i.e., following Spoor's orientations). The phrasing of "transverse/axial" was selected to bridge the terminologies used in respectively in paleoanthropological and medical literature. We clarified this point in the Methods section.

Reviewer #2 (Remarks to the Author):

I co-reviewed this manuscript with one of the reviewers who provided the listed reports. This is part of the Nature Communications initiative to facilitate training in peer review and to provide appropriate recognition for Early Career Researchers who co-review manuscripts.

We thank the reviewer for their careful inspection and comments of this manuscript. See our answer to Reviewer #1 comments.

Reviewer #3 (Remarks to the Author):

The manuscript "Bony labyrinth morphology reveals deep population history and isolation in prehistoric northeastern Africa" is a very interesting study that tackles an important question about population history in northeastern Africa through the analysis of bony labyrinth morphology. The dataset is excellent, the methodological approach is solid, and the results are not only a great contribution to the anthropology and archaeology of northeastern Africa, but also potentially valuable for regions where ancient DNA is unavailable. The paper is generally well organized and clear, and the results have strong potential to advance our understanding of the morphological evolution of human populations during the Late Pleistocene and Holocene. Before being considered for publication, the manuscript would benefit from several clarifications and changes before it can reach its full shape.

Main comments

1. Context and framing: The introduction could do more to situate the study within existing literature from the region. Previous work using dental morphology in the Nile Valley (e.g., Irish 2005; Irish & Usai 2021; Martin et al. 2024, 2025) has already established key patterns of continuity and replacement. It would help readers if the authors briefly summarize what those studies showed and explain how the present work expands on them. Similarly, a short paragraph outlining the main archaeological expectations for each region would make the objectives and hypotheses much clearer.

We thank the reviewer for this suggestion, and we agree with them about the clarity and contextualization this would provide. We therefore included in the introduction the suggested elements and paragraphs.

2. Geographic and authorship imbalance: All authors are affiliated with European or North American institutions, despite the study's focus on African materials. This is increasingly recognized as an issue in our field. It would be appropriate to acknowledge the role of local collaborators and curators more explicitly, and to provide transparency about data access and permits—also mentioning if African researchers were involved in field or curatorial work.

We thank the reviewer for drawing attention to this important issue, and we fully agree with their assessment. To improve transparency and to appropriately acknowledge the contributions of local collaborators, we have added a detailed summary in the Supplementary Material (Note S2). This note outlines the history and status of each collection, specifies the institutions and curators responsible for their stewardship, and clarifies the involvement of local archaeologists, curators, and field teams in the original excavations and subsequent curation. We believe this addition better reflects the collaborative

nature of the work and provides clear documentation of data access, permissions, and the contributions of African colleagues and institutions.

3. Sampling and representativeness: The sample from the Nile Valley is strong and well distributed chronologically, but the comparative material from the Horn of Africa and Central Africa is very small (n = 7–10). This limits what can be inferred about those regions. Contrary to what other reviews might say, I think it is a good idea to include those samples despite their small number; however, the authors should explicitly acknowledge this limitation and frame those observations as preliminary or exploratory.

We thank the reviewer for their comment and we strongly agree with them about the interest in including these assemblages in the study. To better acknowledge their small sample sizes, we nuanced the sections on these results both in the Discussion and Conclusion of the manuscript, clearly stating that these are exploratory.

4. Comparative framework/Neanderthal sample: Including Neanderthals in the same PCA and DSM spaces as Homo sapiens greatly inflates interspecific variance, which dominates the first principal component and makes it harder to discern the subtler population-level differences among H. sapiens. In the current analysis, PC1 is almost entirely driven by features distinguishing Neanderthals (smaller and lower posterior semicircular canal, narrower anterior canal, larger lateral canal), which overshadows the within-human variation the paper aims to interpret. This design choice compresses H. sapiens diversity and partly masks contrasts that are central to the study—such as those between Late Pleistocene/Early Holocene foragers and Neolithic producers in the Nile Valley. Because the study's main question concerns population structure within Africa, the Neanderthal sample should be excluded from the main statistical analyses. European Upper Paleolithic H. sapiens specimens provide a sufficient comparative outgroup without distorting the variance structure. This change would also improve the interpretability of downstream analyses (bgPCA, PERMANOVA, and disparity metrics), all of which are computed from PCA axes currently dominated by archaic–modern separation. Re-running these on H. sapiens only would make the distances and group classifications more meaningful and the discussion of the results more deeply connected to the aims of the study. The same applies to the allometric regressions, where results should be confirmed in the H. sapiens-only dataset to ensure that any size effects are not driven by interspecific differences. Importantly, the "outlier" Holocene individuals identified as morphologically "Neanderthal-like" because of shortened or thickened canals should remain outliers even in a H. sapiens-only analysis. Demonstrating this explicitly would strengthen the argument that their unusual morphology is truly exceptional within H. sapiens, rather than a by-product of including Neanderthals in the same morphospace. A sentence in the Discussion could clarify this: "Because our research question concerns intra-H. sapiens variation, we focus on analyses restricted to modern humans; ordinations including Neanderthals are presented in the Supplementary Information for reference." The figures would also benefit from showing both versions: the PCA including all taxa, and a second set with H. sapiens only, using the same axis limits for direct comparison.

We thank the reviewer for this thoughtful and detailed comment. We fully agree in principle that including Neandertals in a primarily *Homo sapiens*-focused analysis can, in some contexts, inflate inter-specific variance and potentially rotate principal component axes in a way that compresses within-species variation. For this reason, we conducted a series of checks to verify whether this effect occurred in our dataset and that were missing from our original submission.

First, we re-ran the PCAs excluding Neandertal specimens entirely. These analyses produced nearly identical morphospaces to those including the Neandertals, with PC1-2 still overwhelmingly driven by the same set of features (see graph below, and new SI Fig. S6)—especially the distinctive canal morphology of the Holocene individuals we identified as outliers. The only exception being in the 2D measurement analyses were PC1 and 2 got reversed. Still, in all, the axis structure is not determined by the Neandertals but by the highly unusual morphology of these Holocene individuals themselves. This confirms that their "Neandertal-like" morphologies are not an artefact of including Neandertals in the morphospace but represent genuinely exceptional values within *H. sapiens* variation.

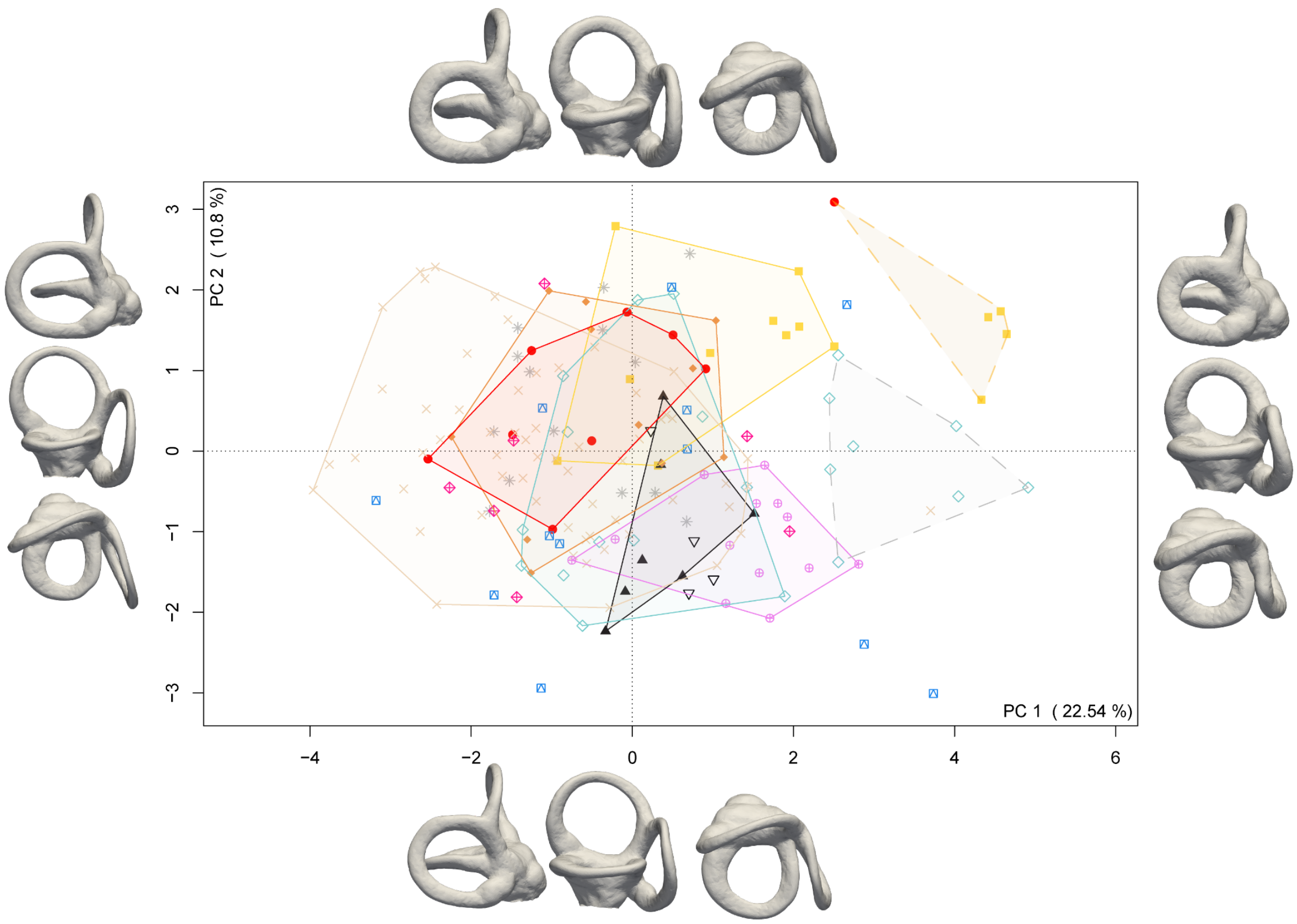

PCA computed on the DSM data of the H. sapiens *only sample*

Because the results with and without Neandertals are so similar, excluding the Neandertals would not change the interpretation of the PCA, and in our view would reduce rather than increase clarity: including Neandertals provides a valuable external reference that allows the reader to more clearly situate—and therefore better appreciate—the magnitude of deviation represented by these outliers. Their inclusion also helps counterbalance the overwhelming weight of the non-outlier *H. sapiens* sample relative to the very small number of outlier individuals, thereby improving the readability and interpretability of the morphospace.

Second, to address the potential influence of Neandertal data on downstream analyses (bgPCA, PERMANOVA), we emphasize that, as stated in the Methods, all such analyses were conducted exclusively on the non-outlier Nile Valley sample, with Neandertal specimens and outlier

individuals entirely excluded. This design prevents interspecific morphology or extreme outliers from influencing the variance structure of the tests. Similarly, we confirmed that the disparity metrics and allometric regressions are not driven by interspecific differences: re-running them with Neandertals removed yielded the same results. For the disparity metrics, we replaced the original plot with the new *H. sapiens*-only analysis in Fig. 3 (now Fig. 4) and specified in the Methods that this metric was computed from a PCA recalculated on the *H. sapiens* sample alone. For the allometric regression, the newly computed values have been added to Supplementary Table S1 alongside the whole-sample results.

Finally, regarding the suggestion to include a second set of PCA plots based on *H. sapiens* only, we understand the reviewer's interest. Because the morphospaces are nearly identical and the inclusion of Neandertals helps contextualize the exceptional morphology of the outliers, we retained the PCAs in their original form in the main text. However, we included the *H. sapiens*-centered analyses in the Supplementary material (SI Fig. S6) along with the additional allometric regressions (SI Table S1), referencing them in the main text, to illustrate this point to the reader.

Overall, these additional checks confirm that Neandertal inclusion does not distort or bias the analyses, and we therefore retained the original comparative framework to maximize interpretability and morphological context for the reader.

5. Intra-observer error: The authors present a PCA but it would be more helpful to see a statistic testing of those differences are significant or not for each method used in the study (landmarks, measurements, deformation-based).

We thank the reviewer for this comment. As detailed in our response to Reviewer #1, comment 10, we have added a new analysis quantifying intra-observer error for the landmark-based protocol, which is the most sensitive to operator placement.

In addition, as requested by Reviewer #1 also, we performed a segmentation reliability test using the deformation-based (DSM) approach. Because each segmentation iteration resulted in the preparation of a new surface mesh, this analysis simultaneously tested both segmentation and mesh preparation variability. The results showed no significant differences between repeated segmentations, confirming the high reproducibility of this approach.

We did not perform additional intra-observer error tests for the remaining deformation-based and 2D measurement-based approaches, as their reproducibility under comparable, standardized protocols has been extensively documented in previous studies cited in the manuscript. In these approaches, operator input is either minimized or constrained to well-defined anatomical measurements, and intra-observer variability has been shown to be negligible relative to biological variation.

6. Presentation of results: The text mentions that all analytical approaches yielded "similar results" but it never specifies what those common patterns are. A short synthesis at the beginning of the Results section—summarizing the general patterns of continuity, discontinuity, and outliers observed across the three methods—would help orient readers before the detailed descriptions of the results obtained through each method.

In accordance with the reviewer's comment, we have added a short synthesizing paragraph at the beginning of the Results section. This introductory synthesis summarizes the major patterns of continuity, discontinuity, and the set of recurrent outliers consistently recovered across the three analytical approaches (DSM, 2D, and LM). Because the 2D and landmark analyses yielded the same

overarching patterns as the DSM approach, and their detailed descriptions are provided in the Supplementary Information, this new paragraph clarifies this concordance while orienting the reader before entering the detailed DSM-based results.

In addition, the figures could be made clearer and more self-explanatory:

o Figure 1: Please create a new figure for the variables displayed in Figure 1B. At the moment they are too small and mixed with information on geographic and chronological information of the samples.

We thank the reviewer for this suggestion and agree with them. We dissociated Fig. 1B from Fig. 1A and 1C to make it a standalone figure.

o Figure 2: add concise labels or inset diagrams summarizing what PC1 and PC2 represent biologically (e.g., variation in posterior canal height, anterior canal width, cochlear coiling). Consider labeling or highlighting the 13 "outlier" individuals in the PCA plots and marking Nile Valley time periods with different symbols or ellipse outlines for clarity. As it is now, since there are many groups, the interpretation is a bit confusing.

Given the large number of groups, analyses and information represented in this figure, we felt that adding further labels or inset diagrams would make it more difficult to read, as the figure is already visually dense. Nevertheless, to better illustrate the biological meaning of the principal components, we have added a new Supplementary Figure showing deformation heat maps, which more clearly illustrate the main morphological variations captured by the PCA axes.

Finally, the 13 outlier individuals are already labelled in the PCA plots. To further improve clarity, and as suggested by Reviewer 1, we revised Figure 2 to enhance its readability by simplifying the representation of groups in the main panels.

o Figure 3: include confidence intervals and sample sizes for the disparity metrics, and note directly in the caption that small n values for the Horn and Central Africa make these estimates exploratory.

We thank the reviewer for this comment. As noted in the caption and described in the Methods, the disparity metrics in Figure 3 (now Fig. 4) are based on 20,000 bootstrap replicates. The density plots generated with the *dispRity* package therefore display the entire bootstrap distribution for each group, which directly represents the associated uncertainty. For this reason, adding separate confidence interval bars would be redundant, as the distribution already contains the confidence intervals (which correspond to the quantiles of the bootstrap samples). Also, as mentioned in a previous answer to the reviewer, we revised the manuscript to clearly state that the results presented for the Horn and Central Africa should be considered as exploratory, given the small sample size.

o Figure 4: indicate whether the inner-ear pathologies (EVA, CNC stenosis, fistulas) were measured or scored as present/absent, and show the diagnostic thresholds (e.g., ≤2 mm for EVA at the operculum, ≤1.8 mm for CNC) so the reader can understand the criteria.

We updated the figure following the reviewer's suggestion.

7. Novel contribution: At several points the discussion reads as confirmation of patterns already

described in the dental record. The paper would be stronger if it emphasized what new information the results of the bony labyrinth data adds—perhaps a deeper temporal signal, a different sensitivity to substructure, or evidence of evolutionary constraint compared to more plastic traits.

One of the explicit goals of the study was to test whether bony labyrinth morphology carries sufficient resolution to track micro-evolutionary population history at the same scale as dental morphology. Because comparable dental data exist only for the Nile Valley so far, this region provides the only context in which a direct, population and individual-level comparison of the two systems is possible (see also answer to Reviewer #1's comment n°6). Demonstrating consistency between labyrinth and dental patterns in this limited geographic subset is therefore a central result of the study: it validates the use of the bony labyrinth as an independent proxy for micro-evolutionary structure, and shows that this bony feature can document population relationships with great details and in agreement with what have previously been inferred from dental traits. This demonstration is crucial, in our view, to proceed with certainty in the interpretation of the broader regional context of the study.

We clarified this objective in the Introduction and Discussion and emphasize that the agreement in the Nile Valley is not meant as a repetition of known results, but as critical evidence for the reliability and temporal sensitivity of labyrinth morphology at this micro-evolutionary scale.

8. Pathological individuals: The section on individuals with "Neanderthal-like" labyrinth morphology is fascinating but needs more caution. It is not clear how "pathological" was defined or how consistent these diagnoses are across individuals. Some of these differences could reflect taphonomic deformation or natural variation rather than congenital syndromes. Please clarify the diagnostic criteria and consider alternative explanations, including de novo mutations or random developmental variation.

The term "pathological" in the manuscript refers to individual with at least one of the inner-ear specific pathologies described and investigated in the study (i.e., fistula, EVA, or CNC stenosis). As the same threshold and diagnostic criteria were used for each of these pathological traits throughout the study, these are very consistent. To clarify this point in the main text, we included a sentence referring to the section mentioning the recording of these traits at the first mention of these pathological aspects. In this regard, while a great majority of "Neandertal-like" individuals do present pathological evidence, the use of the term "pathological" in the text does not *solely* refers to these outlier individuals.

Regarding potential taphonomic deformation or preservation state influence on the diagnoses, we agree with the reviewer that this might be a major concern in recording pathological evidences. As detailed in our answers to reviewers #1 and #2 comments, we were extremely careful to this matter while recording these traits. We clarified this point in the method section and in the Supplementary Material.

Finally, regarding alternative explanations, we do not consider "natural variation" a plausible source for the patterns observed. As shown in Fig. 5, these individuals fall well outside the morphological range of more than 100 other *Homo sapiens* from very diverse geographic and chronological contexts. Moreover, the outliers exhibit a recurrent combination of semicircular canal, vestibular, and cochlear variations—features that appear with an unusually high frequency, and in several cases bilaterally. This level of internal consistency, combined with the presence of clinically documented congenital inner-ear anomaly in almost all of these individuals, is not compatible with random developmental noise or isolated *de novo* mutations, which would be expected to produce idiosyncratic, not systematically repeated, patterns. Nevertheless, as detailed in the answer to the following comment of the reviewer, these interpretations are now tempered in the manuscript by the limitations we explicitly acknowledge

9. Interpretation of evolutionary processes: Some claims about isolation, endogamy, and fixation of mutations are currently too strong given the available evidence. These should be presented as possible scenarios rather than established facts. A short limitations paragraph at the end of the discussion would also strengthen the credibility of the paper.

We agree that some parts of this section may seem too assertive and should be framed more cautiously. In response, we have revised the relevant paragraphs of the Discussion to present these processes as possible scenarios rather than established outcomes. Specifically, we rephrased some parts with nuanced language and we added a new "limitations" paragraph acknowledging uncertainties in inferring population isolation from anatomical data alone

Minor points

• In the introduction, adjust the phrasing "we propose using the Nile Valley as a reference" to "we use the Nile Valley as a reference."

Done.

• Also in the final paragraph of the introduction, explicitly state that the dataset includes individuals with pathological features and justify why they were included.

We thank the reviewer for this suggestion. However, we chose not to mention pathological individuals in the final paragraph of the Introduction because the presence of these individuals is not an *a priori* inclusion criterion but rather an outcome of the study. The dataset was assembled to broadly sample *Homo sapiens* variability, and the identification of individuals with inner-ear pathologies emerged from the analyses themselves (Results, Fig. 5, Table 2, Supplementary Material). Including this detail in the Introduction would therefore preempt the results and is conceptually inconsistent with the structure of the manuscript.

• Indicate whether fistulas and other inner-ear conditions were measured or simply recorded as present/absent.

We made this point clearer in the methods section, but also in Figure 5, following a previous comment from the reviewer.

• Figures should specify the percentage of variance explained by each axis and use more clear symbols for region or chronology.

We thank the reviewer for this suggestion. We would like to clarify that all PCA plots already indicate the percentage of variance explained by each axis directly in the axis labels (e.g., "PC1 (23.36%)" in Fig. 3A). As for the readability of the figure symbols, we followed the recommendation of Reviewer #1 (comment n°5).

• A few minor typos (e.g., "to to") and some small inconsistencies should be corrected.

We thank the reviewer for this. We corrected all typos we could find in the manuscript.

• Supplementary data 1 and 3: when I download them, they are PDFs, but as a table with multiple columns it should be presented as xls or csv.

We submitted these files as .xls documents. There might have been an issue in the up- or downloading of these.

Overall assessment: This is a strong and potentially influential paper. The dataset is unique, and the integration of morphometric and pathological evidence is original. With some reframing of the introduction, clearer statements of limitations, a small redesign of the comparative framework regarding Neanderthal samples, and a more cautious interpretation of the pathological cases, the manuscript could make a significant contribution to the field. I recommend major revision.

Reviewer #4 (Remarks to the Author):

This paper is a very interesting study on morphological approaches to investigate population history and variability in northeast Africa. It specifically, and convincingly, demonstrates the usefulness of bony labyrinth morphology to complementing aDNA and other morphometric analyses (dental morphology) and it thereby opens new possibilities where the two former approaches are not fully available. It involves 148 human individuals from a wide range of geographical locations, including Nile Valley, Horn of Africa, European, South and central African specimens, from late Pleistocene to late Holocene sites, covering a time span between 44 to 3 ka, i.e. from both forager and food-producing societies. The article is very thoroughly researched and substantiated on the basis of past and current research results. The data and results are comprehensive and well-presented.

The results would support the authors' conclusions regarding, for example, the persistence of forager-related population groups well after the transition to Neolithic lifeways and the cohabitation of “two biologically distinct populations”, at least in some areas of the Eastern Sahara margins. Moreover, the authors suggest that there was a high degree of “biological discontinuity at the Neolithic transition in the region”, i.e. “the arrival of food-producing practices in the Nile Valley through migrations starting 8000 years ago.”

While this interpretation generally requires more discussion, the result would prima facie support the idea of non-African origins of food-producing techniques, i.e. a knowledge import of food-producing live-ways from western Asia via human migration, parallel to the direct import of domesticates of sheep, goats and cattle, as well as cultivated plants around 8000 years ago. There are a number of relevant studies which are not cited, but which may be useful in the context, e.g.:

Vermeersch, P.M., Linseele, V., Marinova, E., Van Neer, W., Moyersons, J. and Rethemeyer, J. 2015. Early and Middle Holocene human occupation of the Egyptian Eastern Desert: Sodmein Cave. African Archaeological Review 32: 465– 503.

Xia X, Qu K, Wang Y, Sinding MS, Wang F, Hanif Q, Ahmed Z, Lenstra JA, Han J, Lei C, Chen N. Global dispersal and adaptive evolution of domestic cattle: a genomic perspective. Stress Biol. 2023 Apr 18;3(1):8. doi: 10.1007/s44154-023-00085-2

However, this reviewer sees two interrelated principal problems with the interpretation of the analysis results that the authors may want to consider before such wide-ranging conclusions are proposed. One is that there is a wide gap in the regional representation of the 148 individuals included, and that there is no material from the Lower Nile Valley and adjacent regions. The sample only includes one specimen from Egypt (Nazlet Kater 2), which is obviously not representative for this part of Northeast Africa, and there are no Lower Nile Valley specimens from the Holocene period at all. While this reviewer appreciates the difficulties with working on human remains from modern excavations in Egypt, there are plenty of relevant specimens available for modern analysis in overseas museums and collections. These should be included in a study of such a broad research scope given that the northern Nile Valley, Nile Delta and Red Sea coast may well have been the key region of potential contact for early human groups using food-producing techniques with local African populations in the first place. What if those individuals who were of a distinctly non-African descent had no knowledge of food production? Or what if local Africans brought food-production to Africa themselves?

We thank the reviewer for this important comment. We fully agree that the Lower Nile Valley is central to discussions of early population contacts and the spread of food production, particularly if a Levantine origin for these practices is considered. We have therefore included the suggested references in the main text to emphasize this contextual element. However, the absence of individuals from this region in our dataset reflects the current state of the archaeological record rather than a deliberate sampling choice. Early- and mid-Holocene skeletal assemblages from Egypt are extremely scarce. To our knowledge, no other Late Pleistocene to Early Holocene assemblages from the Lower Nile Valley were available for study. Similarly, for the Middle Holocene, only a very few assemblages exist in the region, and we were unable to access them. The only potential exception is the remains from El-Badari. While access to these remains would be possible, their inclusion is problematic due to the age of the excavations, the lack of recent and secure dating, and the fact that their cultural horizon marks the beginning of a major period of cultural complexification in the region. Including them would have added considerable chronological and interpretive complexity to an already dense and complex study.

Other, more recent samples may exist in museums, but their socio-cultural context would similarly introduce additional complexity, limiting meaningful interpretation. Nazlet Khater 2 is therefore included not as a representative sample for the Lower Nile Valley, but because it is one of the few relevant specimens available and allows us to test its affinities with later populations from the region.

Importantly, our interpretations are explicitly restricted to the areas for which data exist (i.e., the Middle Nile Valley), and they align with previous dental and archaeological evidence for the this period in this region. We do not wish to extend these conclusions to the Lower Nile Valley, nor to make claims regarding the biological origins of food production in Egypt. To ensure this distinction is clear to readers, we have revised the manuscript to specify “Middle Nile Valley” when discussing the documented morphological discontinuity at the Neolithic transition.

We appreciate the reviewer’s emphasis on this region and agree that any future early/mid-Holocene material from the Lower Nile Valley will be essential for refining broader reconstructions of population history in northeastern Africa.

The authors of this study do emphasize the at times high degree of population variability in one and the same region or site (e.g. Shaqadud) and on the same chronological trajectory as well as "rare and localized instances of potential admixture or continuity between the last foragers and the earliest food-producing groups". In particular, those two individuals at Shaqadud, having been buried close to each other within a close temporal relationship but very different ancestry are interpreted as a possible result of the "cohabitation of two distinct biological populations". This reviewer would be more cautious to speak of populations when individuals are involved.

We agree with the reviewer that "population" may be a too strong phrasing for these two individuals. We rephrased this point in the manuscript.

The other, related, issue is hence the interpretation of a limited number of individuals from a variety of geographically dispersed sites in the context of such significant socio-cultural processes as the Neolithic transition in Northeast Africa, which probably took place over more than a millennium. In view of the high degree of mobility of early Holocene groups inhabiting this region, there is also the possibility of local African forager groups in Northern Egypt expanding their scope of interest to areas in the Southern Levant and thereby adopting food-producing techniques themselves. Human migration, especially of the disorganised variety, is always a possibility when it comes to the transfer of knowledge, but it is rarely a one-way road when human populations had not adopted a sedentary life-style in the first place.

We thank the reviewer for this insightful comment. We agree that our sample is limited in number and geographically dispersed, and that the Neolithic transition likely involved complex, multi-directional interactions over more than a millennium. Our study focuses on biological evidence, documenting morphological discontinuities in the Middle Nile Valley, and does not attempt to reconstruct the full socio-cultural processes of food-production adoption.

We acknowledge that local forager groups may have independently adopted food-producing techniques through contact with neighboring regions, and that knowledge transfer could have been multi-directional. The persistence of forager-like morphological traits alongside early food-producer traits in our dataset (e.g., Wadi Shaw and Shaqadud individuals) already captures this complexity. Our interpretations remain restricted to the available dataset, highlighting population-level biological patterns rather than assuming unidirectional migration or cultural diffusion. We have clarified this point in the manuscript to ensure readers understand that our findings pertain to the Middle Nile Valley and adjacent areas, and should not be overgeneralized to the broader Nile Valley or beyond (see also answer to the previous reviewer's comment).

There are indeed numerous avenues to interpret the research results achieved in this interesting study and it would be helpful if these were sufficiently considered in the text, given the paper's title and key observations. On the other hand, given the lack of material from the Lower Nile Valley, it may perhaps be prudent to reduce the interpretative scope of the paper.

We agree with the reviewer that there are indeed numerous ways to interpret the results of this study from social, cultural, and archaeological perspectives. This breadth of potential interpretation is precisely why we have focused our discussion on the biological aspects, which are directly supported by the data. Our goal is to provide a solid, evidence-based foundation that can then serve as a springboard for future integrative studies. We hope that our findings will stimulate re-evaluation,

broader discussions, and collaborative research across disciplines—including archaeology, paleoanthropology, archaeozoology, and archaeobotany—ultimately contributing to a more comprehensive understanding of these key periods in northeastern Africa's past.

REVIEWER COMMENTS

Reviewer #1 (Remarks to the Author):

This revision has resulted in a much improved manuscript, with additional analyses that help to support the robusticity of the results. In particular, one of the central aims—testing the differential population history signal of EDJ and bony labyrinth morphology in the Middle Nile Valley populations—has been well expanded. Additional points arising from revisions, as well as points left unaddressed, are outlined below.

Major points:

1. Study Design, justification: The introduction is much improved, providing three key aims for the study in the penultimate paragraph. The second aim is unclear with regards to what "elsewhere" refers to. Is it referring to Horn of Africa, Central Africa, and South Africa, i.e. sub-Saharan Africa? Is the sampling justification then related to the third aim of assessing whether phenotypic patterns correspond to climate change and the hypothesized "ghost Green Saharans?" The third aim gives the impression that there will be an explicit analysis of samples from humid versus arid phases, but this is missing from the study design. If this is retained as a central aim, some solutions include adding paleoclimate markers to the timeline in Figure 1B, the designation of humid- versus arid- derived samples in Table 1, and additional/modified corresponding exploratory and statistical analyses. Otherwise, this would instead be part of the Discussion rather than a central aim.

We thank the reviewer for this helpful comment. We have clarified the second aim by specifying that "elsewhere" refers to the Horn of Africa and Central Africa mentioned in the preceding sentence.

Regarding the third aim, we do not formally test the impact of environmental conditions (e.g., arid versus humid phases) on labyrinth morphology. As we aimed at stating in the Introduction, our objective is to identify patterns of biological continuity and discontinuity and to discuss them in light of broader paleoclimatic changes. Climate therefore provides an interpretative framework rather than a variable subjected to explicit statistical testing. We have modified the wording of the third aim in the Introduction so that climatic dynamics are presented explicitly as an interpretative framework rather than as a tested explanatory variable, this will avoid any misunderstanding regarding the study design.

We agree that a formal comparison between "arid" and "humid" samples would be valuable. However, current paleoclimatic reconstructions lack the spatial and chronological resolution needed to securely attribute all individual sites or specimens to specific climatic conditions. Assigning such categories would therefore risk overinterpretation. Instead, we situate our results within broader regional trends of climatic evolution and shifting connectivity versus isolation.

2. Study Design, comparative samples: the revisions have adequately explored the effects of excluding the Neanderthal sample. Similarly, it would be beneficial to test the effect of removing the recent (Late Holocene) South African sample or, at minimum, resampling that group to test the effects of disproportionate sample size (e.g. results from Table S1, Homo sapiens sample results in Table S4, results of Figure S7, S13, etc.). Regarding the calculation of momenta, the point that is still unaddressed is whether reducing/excluding the South African sample will have an effect. The authors, in their rebuttal, have a clear hypothesis that "…such effects are not expected to meaningfully alter the resulting momenta or their interpretation…." Now it is time to test that hypothesis to further show that the results are robust.

To test the effect of the large sample size of the South African sample, we recomputed the analyzes presented in the manuscript after removing the data from these recent individuals (i.e., in a similar fashion to what we did previously with the Neandertal specimens). This revealed no differences in the subsequent PCA (e.g., in the DSM analysis new **SI Fig. S8A**), allometry assessment (OLS; **SI. Table S1**), nor in the identification of the outlier individuals (new **SI Fig. S8B**).

As for the impact of the inclusion of these South African individuals in the calculation of momenta, we recomputed the deformation parameters after removing the recent SA individuals from the original atlas. We then submitted the obtained momenta to PCA (new **SI Fig. S19**) and compared it to the previously computed analysis on the original dataset without the South African sample. Here also, no differences were found between each dataset. A Mantel test comparing the Euclidean inter-individual distance matrices derived from the two PCAs revealed an almost perfect correlation ($r = 0.990$, $p = 0.0001$; 9,999 permutations), indicating that the overall morphometric structure is nearly identical between the two computational approaches. We included this information in the Methods section.

3. Study Design, use of bilateral labyrinths: the added Figure S19 is very useful for visualizing variation within individuals. It is still unclear whether each labyrinth is being treated as one case in the other analyses e.g. OLS, PERMANOVA, etc. For example, it is unclear whether sample size has an effect on those results and to what degree this is affected by the inclusion of individual cases represented twice within each group. As an example, let's say a group comprises 5 individuals. In this sample, each individual can be counted twice (left AND right ears each) such that N=10. Alternatively, they could be counted only once (left OR right ears OR a mix thereof) such that N=5. The methodological decision is likely trivial with large sample sizes (e.g. recent South Africa) but could be consequential for small sample sizes (e.g. archaeological samples). One solution could be to resample the left and right data to test the effect on statistical results and/or to specifically account for asymmetry. In addition, a rarefaction analysis could be applied to downsample groups with larger sample sizes and/or duplicates (right AND left) of the same individuals. At the heart of the matter is whether asymmetry differs between groups and whether these differences could be variable spatiotemporally and associated to differential behavior: see, e.g. work by Ward et al. 2022 (AJPA: https://doi.org/10.1002/ajpa.24465) on directional and fluctuating asymmetry across populations with diverse subsistence strategies. With regard to the interpretation of Fig. S19 (lines 627-629), it's unclear how the variation reflects differences in canal thickness rather than shape. The arrow directionality (i.e. left-to-right ears) along the morphospace appears rather stochastic rather than unidirectional, i.e. in Fig. S19, the canal thickness seems to be larger along the positive ends of PC1 and PC2, but the arrow directionality is not uniformly toward that plot quadrant.

In all multivariate analyses (including PCA, OLS, and PERMANOVA), each labyrinth was treated as a single observation, as previously stated in our rebuttal. We have now clarified this explicitly in the main text.

We agree that, in principle, counting left and right labyrinths separately could inflate sample size and potentially bias results, particularly in small archaeological datasets. In the present study, however, this issue has only a negligible impact. First, as shown by additional tests conducted on the large recent South African sample (see response to Comment #2), variation in sample size does not meaningfully affect the PCA structure or OLS results (**Table S1, Fig. S8**), even when the entire sample of 60 labyrinths is removed. Second, for the PERMANOVA analyses, the dataset was restricted to Middle Nile Valley individuals after exclusion of the "Neandertal-like" outliers, as described in the main text. Within this subset, only a single individual (EBK_N_T125; **Supplementary Data S1**) is represented by both left and right labyrinths. All other bilaterally represented individuals either belong

to the excluded outlier group or to comparative samples that were not included in PERMANOVA computation. Therefore, duplication of individuals does not materially affect these statistical outcomes.

We acknowledge that both directional and fluctuating asymmetry may vary across populations and behavioural contexts, as highlighted by Ward et al. We also agree that rarefaction or left/right resampling approaches can be informative in systematically paired datasets. In our case, however, bilateral representation in the archaeological material is extremely limited, and most such individuals are isolated within their respective chronocultural groups (**Supplementary Data S1**). This scarcity precludes any robust spatiotemporal assessment of asymmetry patterns or meaningful intergroup comparison. As noted in our previous rebuttal, the inclusion of bilateral specimens was primarily motivated by the need to document unilateral pathological cases that could have been overlooked if only one side had been analyzed.

Finally, regarding **Fig. S19** (now **Fig. S21**), we acknowledge that arrow directionality is not strictly uniform. The purpose of this figure is therefore primarily to illustrate the magnitude of within-individual variation rather than to demonstrate a consistent directional asymmetry pattern. However, many L-R displacements broadly align along a similar lower-left to upper-right quadrants axis of the PCA, that can be associated with larger canals as mentioned by the reviewer. We have revised the text to describe this trend more cautiously, referring to minor but general trend between sides, rather than attributing it to a uniform asymmetry pattern.

4. Methods, landmark pre- and post-processing: The authors should clarify the steps associated with landmark processing. The added content in lines 544-551 is confusing because it appears that the B-spline was applied in the shapes package, which I don't think is the case (and is certainly not reflected in the R code included). Briefly clarify whether the equidistant algorithm was applied in Avizo, for example, or include the proper R package in the main text and R code in the supplementary information.

We thank the reviewer for pointing this issue. The resampling step was carried in R, using the B-spline coordinates and the *resampleCurve()* function implemented in the *Morpho* package. We clarified this step in the main text and Supplementary Code.

5. Methods, intraobserver measurement error test: the new analyses reported in Fig. S17 are good additions, but the authors should note still that a better representation would be one that takes the PCA results of the full dataset and then projects the repeated measures onto that space. The objective for this kind of visualization is that it informs whether variation in measurement error is greater than variation between individuals along specified axes of variation (i.e. whether non-replicates plot within variation of replicates of a single individual). For the Euclidean distance reported in Fig. S17, justify why the first 5 PCs (and not more or less) were used. Lastly, note additional ways of accounting for measurement error that would not necessarily be limited to certain PCs (see again, e.g. Ward et al. 2022).

We replaced the original **Fig. S17** with a new version following the reviewer's suggestion, in which repeated measurements are projected onto the PCA computed from the full dataset. This visualization confirms the low intra-observer error previously described.

In the initial analysis, Euclidean distances were calculated using the first five PCs because this test was performed on a restricted subset of 16 individuals (8 pairs of measurements), in which the first five components each accounted for more than 1% of the total variance and together captured the large majority of variation. In the revised analysis, the PCA is based on the full sample (almost 200

individuals), resulting in variance being distributed across a greater number of components. Applying the same >1% variance threshold in this larger dataset corresponds to the first 18 PCs, which together account for 88.9% of total variance. This standard approach ensures methodological consistency while excluding higher-order components likely reflecting minor or noisy variation. The results remain unchanged, confirming that intra-observer error is negligible relative to inter-individual variation.

Minor points

6. Include “Ref.” prior to stand-alone citations (e.g. Line 516, line 518)

Done.

7. Figure S19: the black convex hull (Nile Valley Late Paleolithic) is difficult to follow with the black arrows: change the color to enhance visualization

Done.

8. Double check the correspondence of the enumerated references in the main text and the bibliography list since several do not match up.

We thank the reviewer for identifying this. We revised the references throughout.

9. The sample group names are still difficult to follow, particularly between the main text and the supplementary information. For example, “Middle Nile Valley” has now been adopted in most parts of the main text but not so in the figures of the main text, nor in several parts of the supplementary files. I recommend establishing names (and possibly abbreviations) in Table 1 and using that as a template to check consistency on all other references to the groups throughout the main text and supplements.

We checked all figures, text and tables in the main text and SI. We revised where necessary to ensure consistency in the names of the groups

Reviewer #2 (Remarks to the Author):

I co-reviewed this manuscript with one of the reviewers who provided the listed reports. This is part of the Nature Communications initiative to facilitate training in peer review and to provide appropriate recognition for Early Career Researchers who co-review manuscripts.

Reviewer #3 (Remarks to the Author):

I appreciate the substantial effort the authors have made in revising the manuscript. Several of the points raised in my previous review have been addressed in a serious and thoughtful manner. In particular, the improved framing of the study within the existing dental and archaeological literature, the clearer description of methodological procedures, and the more cautious and precise language

used when discussing PCA patterns have all significantly strengthened the manuscript. Overall, these changes have improved both the clarity and the rigor of the work.

That said, there are still a few aspects of the revised version that I find less convincing and that, in my view, would benefit from further refinement:

1. The interpretation of individuals described as having “Neandertal-like” bony labyrinth morphologies, together with the discussion of associated pathologies, remains stronger than the available evidence fully supports, despite the added acknowledgment of limitations. I would encourage the authors to further separate the descriptive results from the explanatory scenarios proposed (e.g., isolation, endogamy, fixation of mutations), and to frame these scenarios more explicitly as alternative hypotheses rather than as preferred or implied explanations.

We thank the reviewer for this important comment. We have revised the relevant section to more clearly separate descriptive results from explanatory interpretations We have also softened the discussion of associated pathologies and reframed demographic scenarios (e.g., isolation, endogamy, founder effects) explicitly as alternative, non-exclusive hypotheses. The text now emphasizes that the anatomical evidence alone does not allow us to discriminate among these possibilities.

2. While I understand the authors’ rationale for retaining and interpreting PC2 in the high-dimensional analyses, its prominent role in the Discussion would benefit from a clearer distinction between statistically supported structure and heuristic or exploratory visualization. Making this distinction more explicit would help avoid over-weighting patterns that are primarily exploratory in nature.

We thank the reviewer for this important clarification. We have revised the Results section to make explicit that our interpretation does not rely on PC2 of the DSM analysis alone. While PC2 in the DSM framework does not reach statistical significance, the corresponding axes in the landmark-based and 2D analyses are statistically supported, and all three analytical approaches display consistent patterns of differentiation among the groups under study.

We now explicitly state that PC1 captures the primary axis of differentiation across methods, whereas PC2 provides secondary resolution within Homo sapiens. Our interpretation is grounded in the convergence of statistically supported results across independent morphometric approaches (including PERMANOVA), rather than on exploratory visualization of a single PCA axis. We believe this clarification better distinguishes statistically supported structure from heuristic representation while preserving the biological signal consistently observed across analyses.

3. The additional supplementary material on curatorial histories and data access is a welcome improvement. However, I believe the manuscript would be further strengthened by including a brief reflective statement in the main text that acknowledges broader structural issues related to data access, representation, and authorship in African research contexts.

We thank the reviewer for this thoughtful comment. We updated the Ethics statement section to include such reflective statement.

4. An additional point that became clearer to me after reading the revised manuscript alongside the other reviewers’ reports concerns the geographic scope of the conclusions. Although the authors appropriately note sampling limitations, some parts of the main text and conclusions still read as broadly representative of “northeastern Africa,” despite substantial gaps in regional coverage—most

notably the Lower Nile Valley and Holocene Egypt. I would recommend tightening the language throughout the main text so that the scale of inference more consistently reflects the actual spatial and temporal distribution of the samples, rather than relying primarily on caveats placed in the Supplementary Information.

We have revised the main text to tighten the language regarding “northeastern Africa” and avoid any misunderstanding to the reader. In particular, we replaced several occurrences of “northeastern Africa” with more precise wording such as “adjacent regions and the Middle Nile Valley”.

5. Finally, some interpretive sections of the Discussion still extend beyond what is explicitly demonstrated in the Results, particularly when linking morphological patterns to climatic or demographic processes outside the Nile Valley. While these broader interpretations are intellectually stimulating, the manuscript would benefit from more frequent signposting back to the specific results on which each inference is based, in order to maintain a clear distinction between empirical findings and broader synthesis.

We revised the Discussion throughout to more explicitly refer to the relevant analytical results (e.g., PCA clustering patterns, disparity metrics, and PERMANOVA tests) when proposing demographic or evolutionary interpretations. We also clarified the distinction between empirical observations and broader synthesis by introducing more cautious phrasing where interpretations could extend beyond the immediate dataset.

Minor points:

• Line 93: “The Middle Nile Valley (i.e., the area extending from the First to the Sixth cataract of the Nile, in southern Egypt and Sudan) is one of the few regions where large skeletal assemblages exist.” One of the few regions in Africa? Please clarify.

We clarified this section.

• Line 105: Please briefly explain why “aDNA remains very limited.”

We rephrased this sentence to make this point clearer.

• Lines 136–138: “to interpret patterns observed elsewhere” is too vague; please clarify the intended meaning.

This has been rephrased and clarified.

• Line 155: I suggest revising to “focus on describing the results of the DSM analyses, with more detailed information on the 2D and landmark-based analyses…” rather than “focus on the DSM analyses, with details on the 2D and landmark-based analyses…”.

Done. We thank the reviewer for this suggestion.

• Line 435: It is not clear what is meant by “demographic conditions.”

We replaced ‘conditions’ by ‘scenarios’.

• Lines 464–466: Please specify how many individuals had paired bony labyrinths analyzed.

Done.

• Line 500: I suggest revising to “Given the differential state of preservation…”.

Done.

• Lines 523–531: Please add citations supporting the claim of “the largest comparative dataset possible of previously published inner ear measurements.”

We added the references of the corresponding dataset mentioned here.

• Lines 651–652 and 658–659: The same information appears to be repeated; please consolidate.

We thank the reviewer for identifying this, we deleted the repetition of this information.

I would like to emphasize that my remaining concerns do not call for additional analyses or new data. Rather, they relate to further refinement of the interpretive framework and tone, particularly where exploratory patterns are linked to specific evolutionary or demographic processes. These comments are offered in a constructive spirit: I consider the dataset and analytical work to be of high quality, and I believe that a more explicitly cautious and clearly signposted interpretive stance would ultimately strengthen both the impact and the credibility of the paper.

Reviewer #4 (Remarks to the Author):

This paper considers morphological approaches to investigating population history and variability in northeast Africa based on bony labyrinth morphology, thereby complementing aDNA and other morphometric analyses (dental morphology). Bony labyrinth morphology not only offers new possibilities of investigation in situations where the two other approaches are not fully available, but also—where the data allows—demonstrates the enormous value of using all three morphological systems in combination.

The study involves 148 human individuals mainly from the Middle Nile Valley and a small number of individuals from a wide range of other geographical locations, including the Lower Nile Valley, Horn of Africa, Europe, South and central Africa. The chronological scope covers a wide time span between 44 to 3 ka, i.e. from the late Pleistocene to late Holocene periods, thus involving both forager and food-producing groups. The results show a significant degree of morphological discontinuity between some forager and food-producing groups as well as some evidence for continuity and admixture in other groups in this region.

The article is very thoroughly researched and substantiated by past and current research results. The data and results are comprehensive, well-presented and have great significance for understanding potential population dynamics in the region of the Middle Nile Valley. The wide chronological scope

may have implications for understanding the possible contribution of human migration and ancestral variability of social groups in the process of the Neolithic transition in this part of northeastern Africa.

REVIEWER COMMENTS

Reviewer #1 (Remarks to the Author):

The revisions have sufficiently addressed the methodological and theoretical concerns from previous versions of the manuscript, adding necessary analyses and framing limitations of interpretation from the results provided. The dataset, in particular, will be an important contribution to the field. Some editorial and copy-editing changes might still be necessary.

Reviewer #2 (Remarks to the Author):

I co-reviewed this manuscript with one of the reviewers who provided the listed reports. This is part of the Nature Communications initiative to facilitate training in peer review and to provide appropriate recognition for Early Career Researchers who co-review manuscripts.

Reviewer #3 (Remarks to the Author):

I would like to acknowledge the effort invested by the authors in revising this manuscript. The current version is clearly improved in terms of clarity, methodological detail, and overall framing. Overall, the manuscript is now much more robust. The remaining points mainly concern clarifying a few methodological aspects and tightening the link between the results and their interpretation. Addressing them would further strengthen the paper and make the argument even more solid.

Major points

1. Identification of fistulas and distinction from segmentation artifacts: I would appreciate some clarification on how fistulas were distinguished from potential segmentation artifacts. Given the variability in preservation and scan resolution, it would be helpful to specify the criteria used to identify true anatomical features versus artefactual discontinuities (e.g., consistency across slices, bilateral occurrence, comparison with anatomical landmarks, or validation by multiple observers). Since these features play an important role in the interpretation, a more explicit explanation would strengthen this part of the manuscript.

We thank the reviewer for this comment. As noted in our previous response and clarified in the revised manuscript, fistulas were identified directly on the re-oriented image stacks (transverse and sagittal views), rather than on the segmented 3D volumes. This approach avoids potential biases related to segmentation artefacts.

To distinguish true anatomical features from potential taphonomic alterations, several criteria were systematically applied. First, suspected fistulas were required to be consistently visible across consecutive slices and in both anatomical planes. Second, particular attention was paid to edge morphology, with genuine features expected to present smooth and well-defined boundaries rather than irregular or diffuse discontinuities.

In addition, the preservation state of each specimen was carefully assessed prior to scoring (see Methods and Supplementary Fig. S22), and features were only recorded when preservation and image

quality allowed reliable interpretation. This conservative approach minimises the risk of misidentifying taphonomic or imaging artefacts as anatomical features.

We clarified this in the Methods section.

2. Diagnostic criteria for inner ear pathologies: It would be useful to clarify how inner ear pathologies (e.g., enlarged vestibular aqueduct, cochlear nerve canal stenosis, semicircular canal hypoplasia) were identified. In particular, please indicate whether these diagnoses are based on relative comparisons within the sample or established clinical criteria, and how comparability was ensured across specimens.

As mentioned in the Methods section, the identification of the inner ear pathologies (EVA, CNC, and fistuals) was carried out using established clinical criteria and standard diagnostic thresholds (for EVA and CNC). These identifications are therefore based on absolute criteria rather than relative comparisons within the sample.

For semicircular canal hypoplasia, assessment was based on comparison within the *Homo sapiens* sample, following protocols used in similar work (e.g., Conde-Valverde et al., 2024). As a result, interpretations related to this trait are formulated cautiously in the Results and Discussion sections.

Comparability across specimens was ensured by applying a consistent protocol to all individuals, including standardized image re-orientation and measurement procedures, and by having all observations performed by a single analyst (N.M.). In addition, the preservation state of each specimen was systematically evaluated prior to scoring, and features were recorded as “non-observable” when taphonomic alteration prevented reliable assessment (see Methods and Supplementary Fig. S22).

3. Scan resolution and comparability: Given the relatively wide range of voxel sizes used in the µCT scans, a brief discussion of how differences in resolution might affect the identification of small anatomical features—especially pathological traits—would be helpful.

We thank the reviewer for this helpful comment. The voxel sizes of the µCT scans used in this study range from 17 to 127 µm. While this represents some variability, it remains within (and in many cases below) the range commonly reported in the literature on bony labyrinth morphology (e.g., Gunz et al., 2012; Urciuoli et al., 2025; Beaudet et al., 2019; Spoor & Zonneveld, 1998).

Regarding the characterization of morphological features (e.g., in the DSM analyses), previous work has shown that variation in scan resolution within this range does not significantly affect the results, even when comparing substantially coarser resolutions (e.g., 90 vs. 300 µm; Urciuoli et al., 2025).

With respect to the identification of pathological traits, the criteria applied here are derived from established clinical protocols, as detailed in our response to comment #1 and in the Methods section. These protocols are typically based on medical CT data with coarser resolution than the µCT scans used in the present study. Therefore, the level of variation in voxel size in our dataset is unlikely to affect the identification of the pathological features considered here.

To clarify this point, we have added a brief discussion in the Methods section.

4. Segmentation and measurement procedures: Please clarify who performed the segmentations and who carried out the measurements. If more than one operator was involved, it would be useful to

indicate whether inter-observer consistency was assessed, or whether all procedures were conducted by a single analyst.

All segmentations and measurements (e.g., 2D measurements, landmark digitization, 3D models pre-processing, recording of the pathologies, etc.) were performed by the first author. As suggested by the reviewer, this ensures consistency, with repeatability tests (see Methods) that further confirm robustness. We clarified this in the Methods section, clearly mentioning that all protocols were applied by the same analyst.

5. Interpretation of pathological patterns: The discussion of inner ear pathologies has improved, but I would still encourage keeping a clear distinction between descriptive findings and broader demographic interpretations. Where multiple explanations are possible (e.g., isolation, endogamy, developmental processes), it would be good to continue presenting them as alternative, equally plausible scenarios.

We thank the reviewer for this suggestion. We have revised the Discussion to further clarify the distinction between descriptive results and interpretative scenarios. In particular, we now consistently present all demographic explanations (e.g., reduced connectivity, endogamy, drift, population structure) as alternative and non-exclusive possibilities, and avoid prioritizing any single interpretation. We also reinforce that inner ear anomalies can arise through multiple genetic and developmental pathways that cannot be distinguished in the present dataset, ensuring that the pathological evidence is interpreted strictly within its descriptive limits.

Minor Comments

Line 136: Please revise to: “is also detectable when studying the bony labyrinth.”

Done.

Line 139: Please revise to: “to interpret patterns of biological affinities in these regions.”

Done.

Line 164: Please revise to: “This mainly relates to a combination of characteristic morphological traits observed in Neandertals.”

Done.

Line 421: Please clarify what is meant by “cases” (e.g., individuals, specimens, or pathological instances).

We clarified that this section refers to “individuals displaying unusual bony labyrinth morphologies and/or congenital pathological changes”.

Terminology: Terms such as “affinities,” “similarities,” and “continuity” are used throughout the manuscript and sometimes seem interchangeable. It may help to ensure consistent usage or briefly clarify how these terms are being used in this context.

Throughout the text we used *similarity to* refer to observed morphological resemblance, *affinity* to patterns of resemblance that may suggest biological relatedness, and *continuity* to the persistence of morphological patterns through time within a region. We clarified this in the Introduction and ensured consistency throughout the text.
